# Efficacy and Safety of Once-Daily Insulin Degludec/Insulin Aspart versus Insulin Glargine (U100) for 52 Weeks in Insulin-Naïve Patients with Type 2 Diabetes: A Randomized Controlled Trial

**DOI:** 10.1371/journal.pone.0163350

**Published:** 2016-10-19

**Authors:** Ajay Kumar, Edward Franek, Jonathan Wise, Marcus Niemeyer, Henriette Mersebach, Rafael Simó

**Affiliations:** 1 Diabetes Care & Research Centre, Near Overbridge, Kankarbagh, Patna, Bihar, India; 2 Medical Research Center, Polish Academy of Sciences and Central Clinical Hospital MSWiA, Warsaw, Poland; 3 Tulane Medical School, Department of Endocrinology, New Orleans, LA, United States of America; 4 Market Access and Public Affairs, Novo Nordisk Pharma GmbH, Mainz, Germany; 5 Clinical Development & Research–Diabetes & Obesity, Novo Nordisk Inc, Princeton, NJ, United States of America; 6 Diabetes and Metabolism Research Unit, Autonomous University of Barcelona, Vall d’Hebron Institute de Recerca, and CIBERDEM, Barcelona, Spain; Medizinische Universitat Graz, AUSTRIA

## Abstract

**Purpose:**

The efficacy and safety of insulin degludec/insulin aspart (IDegAsp) once daily (OD) compared with insulin glargine U100 (IGlar) OD over 52 weeks in insulin-naïve adults with type 2 diabetes mellitus (T2DM) was investigated.

**Methods:**

In this open-label, parallel-group treat-to-target trial, participants were randomized (1:1) to receive IDegAsp OD (breakfast, n = 266) or IGlar OD (as per label, n = 264). Participants then entered a 26-week extension phase (IDegAsp OD, n = 192; IGlar OD, n = 221). The primary endpoint was change from baseline to Week 26 in HbA_1c_.

**Results:**

After 26 and 52 weeks, mean HbA_1c_ decreased to similar levels in both groups. After 52 weeks, the mean estimated treatment difference was –0.08% (–0.26, 0.09 95%CI), confirming the non-inferiority of IDegAsp OD versus IGlar OD evaluated at Week 26. After 52 weeks, there was a similar reduction in mean fasting plasma glucose in both treatment groups. The rate of confirmed hypoglycemic episodes was 86% higher (*p* < 0.0001) whereas the rate of nocturnal hypoglycemia was 75% lower (*p* < 0.0001) for IDegAsp versus IGlar.

**Conclusion:**

Nocturnal-confirmed hypoglycemia was higher with IGlar whereas overall and diurnal hypoglycemia were higher with IDegAsp dosed at breakfast. These results highlight the importance of administration of IDegAsp with the main meal of the day, tailored to the individual patient’s needs.

**Trial Registration:**

ClinicalTrials.gov: NCT01045707 [core]) and NCT01169766 [ext]

## Introduction

Type 2 diabetes mellitus (T2DM) is characterized by increasing insulin resistance and an inexorable decline in β-cell function, with patients with T2DM usually requiring treatment intensification to achieve and maintain glycemic control [1,2,3,4]. The loss of mealtime glucose control is an early feature of disease progression in T2DM and control of postprandial hyperglycemia needs to be addressed. Some reluctance to initiate or intensify insulin therapy has been noted among patients and physicians because of fear of hypoglycemia and weight gain, and perceived problems of dependency on the medication and complexity of titration and injection regimens [5,6,7]. Combination therapies in the form of basal plus, basal-bolus or premix strategies were traditionally considered following successful titration with basal insulin only [8,9]. However, systematic reviews and meta-analyses of trials comparing basal insulin versus premix concluded that, despite the higher risk of hypoglycemia, glycemic control was greater with premix insulin [10,11,12]. This was also shown for biphasic insulin. Although regimens based on injections of premixed biphasic insulin can provide prandial coverage for several meals, they may also be associated with an increased rate of nocturnal hypoglycemia [13] as the interaction between the soluble and protaminated insulin components produces a prolonged and uneven peak glucose-lowering effect compared with rapid-acting insulins [14]. Therefore, insulin combinations comprising a long-acting basal and a distinct rapid-acting prandial insulin in a single pen, suitable for once-daily (OD) or twice-daily (BID) administration, may be suitable insulin initiation and intensification approaches.

Insulin degludec/insulin aspart (IDegAsp) is the first soluble combination insulin consisting of 70% long-acting basal insulin degludec (IDeg) and 30% rapid-acting prandial insulin aspart (IAsp) available as a single injection. Owing to a novel protraction mechanism, IDeg concentrations remain constant and demonstrate a glucose-lowering effect over 42 h [15]. As a result, the day-to-day and within-subject variability of the glucose-lowering effect were lower with IDeg compared with insulin glargine (U100; [IGlar]) [16]. Furthermore, IDeg in combination with oral antidiabetic drugs provided long-term glycemic control similar to IGlar with a lower risk for nocturnal hypoglycemia in insulin-naïve patients with T2DM [17,18]. It is hypothesized that these benefits will translate into positive treatment outcomes with IDegAsp, as both components of IDegAsp have been shown to coexist independently in solution as IDeg di-hexamers and IAsp monomers [19], allowing, for the first time, the co-formulation of a basal and a bolus insulin without altering their individual pharmacodynamic properties, and providing a potential treatment option for use of a “basal-plus” regimen in one single injection.

This study investigated the efficacy and safety of IDegAsp administered OD in the morning, regardless of meal-time considerations, compared with IGlar OD in insulin-naïve T2DM participants treated with metformin and ≥ 1 other oral antidiabetic drugs (OADs).

## Participants and Methods

### Trial population

Adults (≥ 18 years old) with T2DM (confirmed ≥ 6 months prior to enrollment), glycosylated hemoglobin (HbA_1c_) 59–97 mmol/mol (7.5%–11.0%), and body mass index ≤ 40 kg/m^2^, were included from 76 centers in eight countries (**Table 1**). Patients were insulin-naïve and inadequately controlled with metformin (including fixed-combination products; 1500 mg or maximum tolerated dose of ≥ 1000 mg daily) and at least one other OAD for ≥ 3 months prior to randomization. Participants were excluded if they were taking glucagon-like peptide-1 (GLP-1) receptor agonists and/or thiazolidinediones in the 3 months prior to trial initiation.

**Table 1:** Participating countries*.

Country	Number of participants (%)	Percentage of total study population (%)
Austria	22 (15)	4.2 (3.6)
India	72 (58)	13.6 (14.0)
Poland	64 (60)	12.1 (14.5)
Russian Federation	79 (54)	14.9 (13.1)
South Korea	31 (26)	5.9 (6.3)
Spain	67 (53)	12.7 (12.8)
Turkey	39 (28)	7.4 (6.8)
United States	155 (119)	29.3 (28.8)
**Total**	**529 (413)**	**100 (100)**

*Full analysis set (FAS: core and extension phase).

### Trial design

The study was a 26-week core trial and a 1-week washout period followed by a 26-week extension in a randomized, open-label, two-arm, parallel-group, treat-to-target trial. A 1-week washout and follow up occurred at Weeks 26 and 53, when treatment was switched to the intermediate-acting insulin neutral protamine Hagedorn (NPH) 100 IU/mL BID (morning and evening) to ensure IDegAsp was washed out prior to measuring insulin antibodies.

The core and extension phases of the trial took place in eight countries: Austria, India, Republic of Korea, Poland, Russia, Spain, Turkey, and the United States. The study was carried out in accordance with the Declaration of Helsinki and its amendments (World Medical Association, http://www.wma.net) and Good Clinical Practice (International Conference on Harmonisation, http://www.ich.org). The trial protocol was approved by the appropriate local institutional review boards and ethics committees prior to initiation. Participants provided informed written consent. The study protocol and a full list of Institutional Review Board members have been uploaded as separate documents (**S1 Text**).

At trial initiation, participants were randomized (1:1) using an interactive voice/web response system to IDegAsp OD or IGlar OD, both in combination with metformin. Except for metformin, any previous OAD treatments were discontinued at randomization. Participants who completed the 26-week core study were eligible to continue in a 26-week extension phase. During the core study, IDegAsp was administered OD with breakfast (morning meal) and the time of administration of IDegAsp could not be changed, according to protocol. In the extension study, a protocol amendment permitted IDegAsp OD to be taken either with breakfast or with the largest meal of the day. IGlar was administered OD at the same time every day throughout both periods, according to label [20].

Self-measured plasma glucose (SMPG) values were collected for three consecutive days prior to a visit and included measurements before breakfast. Dose adjustment of basal insulin was based on the participant’s before-breakfast value, even if the participant did not eat breakfast. The titration algorithm is shown in **Table 2**. After 26 weeks, participants switched to NPH 100 IU/mL BID to measure changes in insulin-specific antibodies during the 1-week follow-up period, after which IDegAsp or IGlar treatments were resumed for participants entering the extension phase, preferably at the same dose level as Week 26.

**Table 2:** Titration algorithm for IDegAsp or IGlar.

Pre-breakfast plasma glucose*		Adjustment
mmol/L	mg/dL	U
< 3.1^†^	< 56^†^	–4 (If dose > 45 U, reduce by 10%)
3.1–3.8^†^	56–69^†^	–2 (If dose > 45 U, reduce by 5%)
3.9–4.9	70–89	0
5.0–6.9	90–125	+2
7.0–7.9	126–143	+4
8.0–8.9	144–161	+6
≥ 9.0	≥ 162	+8

*Mean of three consecutive days’ measurements for dose increases; lowest value for dose decreases.

^†^Unless there is an obvious explanation for the low value, such as a missed meal.

IDegIAsp, insulin degludec/insulin aspart; IGlar, insulin glargine (U100).

### Study endpoints–core-study phase

The primary endpoint was change from baseline to Week 26 in HbA_1c_. Secondary endpoints included the change from baseline to Week 26 in fasting plasma glucose (FPG); prandial plasma glucose (PPG) increment 90 min after start of breakfast as measured by SMPG; changes in 9-point SMPG profiles, insulin dose and body weight; the number of participants who achieved an HbA_1c_ level < 53 mmol/mol (< 7.0%) without hypoglycemic episodes; and the number of overall confirmed and nocturnal confirmed hypoglycemic episodes. Confirmed hypoglycemic episodes included those requiring assistance from another person (severe hypoglycemia) and with a confirmed plasma glucose value < 3.1 mmol/L (56 mg/dL). Although American Diabetes Association (ADA) guidelines define confirmed hypoglycemia by blood-glucose levels of ≤ 3.9 mmol/L (70 mg/dL), the cut-off in this analysis was set at a lower, more sensitive level to reflect the onset of physiologic hypoglycemia symptoms at < 2.8 mmol/L (50 mg/dL). Confirmed hypoglycemic episodes were defined as nocturnal if the time of onset was at 00.01–05.59 h (inclusive). Safety assessments included the reporting of adverse events (AEs), changes in vital signs, insulin antibodies and fundoscopy.

### Study endpoints–extension phase

The primary endpoint in the extension phase of the study was the long-term safety and tolerability of IDegAsp versus IGlar, as assessed by reported AEs, hypoglycemic episodes, and clinical evaluations including changes in insulin-specific antibodies. Changes from baseline to Week 52 in body weight and insulin dose were assessed as primary endpoints. Secondary endpoints included the change from baseline to Week 52 in HbA_1c_, FPG and 9-point SMPG profile.

### Statistical analysis

The primary objective of the core phase was to confirm non-inferiority of IDegAsp compared with IGlar with respect to the change from baseline in HbA_1c_ at 26 weeks using a pre-specified non-inferiority limit of 0.4% for the between-treatment difference. Based on a one-sided *t*-test, a significance level of 2.5%, and assuming a standard deviation of 1.3% for HbA_1c_, a minimum sample size of 446 was calculated to provide ≥ 90% power to demonstrate the non-inferiority objective. Assay sensitivity in the non-inferiority analysis was validated by the treat-to-target design, and supported by weekly visits or phone contact to continue dose adjustment to achieve and maintain glycemic targets.

The primary endpoint was analyzed using analysis of covariance (ANCOVA), with treatment, antidiabetic therapy at screening, sex and region as categorical covariates, and age and baseline HbA_1c_ as continuous covariates. Missing values were imputed using the last observation carried forward (LOCF). Change from baseline in FPG, body weight, 9-point SMPG and prandial increment were also analyzed with ANCOVA using the same categorical covariates as for the primary endpoint, with age and baseline for the endpoint parameter as continuous covariates. Responder analysis was based on a logistic regression model with treatment, antidiabetic therapy at screening, sex and region as categorical covariates, and age and baseline HbA_1c_ as continuous covariates. The number of hypoglycemic episodes was analyzed using a negative binomial regression model with a log-link function and the logarithm to the time period, in which a hypoglycemic episode was considered treatment emergent, as offset. The model included treatment, antidiabetic therapy at screening, sex and region as categorical covariates and age as continuous covariate. The incidence of treatment-emergent AEs was summarized using descriptive statistics. The same endpoints were evaluated at 26 weeks (core phase) and 52 weeks (extension phase), using identical statistical models for both time points.

### Participant analysis sets

For the core-study phase, the statistical analyses of all efficacy endpoints including confirmed hypoglycemia and nocturnal-confirmed hypoglycemia episodes were based on the full analysis set (FAS; all randomized participants). The non-inferiority analysis of HbA_1c_ at 26 weeks was repeated using the per-protocol set. Safety endpoints were analyzed descriptively using the safety analysis set (SAS; all participants receiving at least one dose of IDegAsp or IGlar). For the extension-treatment phase, safety data were evaluated for the SAS (all participants exposed to treatment after randomization, including those who did not enter the extension phase). Efficacy data for the extension phase were evaluated using the FAS including all randomized participants from the core phase, and applying LOCF for participants who did not enter the extension phase. The extension trial set included participants who completed the core phase and were exposed to treatment during the extension phase. No participant changed treatment during the study.

## Results

### Participant characteristics

**Fig 1:**
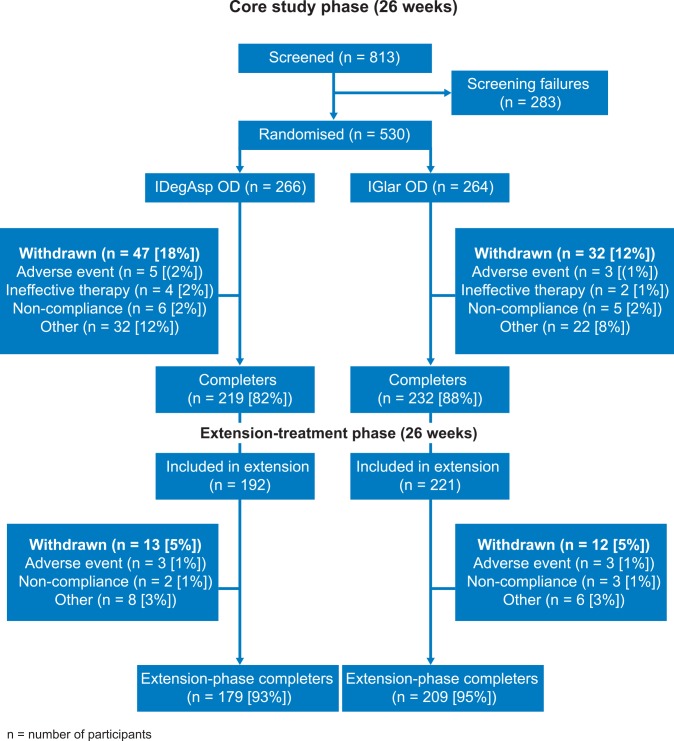
Study flow diagram; core and extension study phases. IDegIAsp, insulin degludec/insulin aspart; IGlar, insulin glargine (U100); OD, once daily; n, number of participants.

Of 813 participants screened for the 26-week core phase, 530 were randomized to receive IDegAsp (n = 266) or IGlar (n = 264); 413 (192 and 221, respectively) entered the extension phase, of whom 179 (93%) in the IDegAsp group and 209 (95%) in the IGlar group completed the study (**Fig 1**). In the core phase, 47 participants (18%) withdrew from the IDegAsp group and 32 (12%) participants withdrew from the IGlar group. Most participants withdrew due to other reasons and only 1–2% (5 *vs* 3 and 4 *vs* 2 participants treated with IDegAsp *vs* IGlar) withdrew due to AEs or ineffective therapy, respectively. In the extension phase, 13 and 12 participants (5%), respectively, withdrew from the study (three in each treatment group due to AEs). A larger proportion of participants in the IGlar group entered the extension phase (84% *vs* 72%). Demographic and baseline characteristics were similar with marginal differences between treatment arms in both treatment phases with the exception of a higher duration of diabetes for IGlar compared with IDegAsp (**Table 3**). The number of participants recruited from each participating country is listed in **Table 1**.

**Table 3:** Participant demographics and baseline characteristics.

	Core-study phase		Extension-treatment phase	
Characteristic	IDegAsp OD	IGlar OD	IDegAsp OD	IGlar OD
**Full analysis set (FAS)^†^, n**	266	263	-	-
**Extension trial set (ETS), n**	-	-	192	221
**Sex, women/men, %**	53.0/47.0	48.3/51.7	52.6/47.4	46.6/53.4
**Race, %**				
**White**	72.9	71.9	70.8	71.0
**Black**	7.9	4.9	9.9	5.0
**Asian**	18.8	21.7	18.8	23.0
**Other**	0.4	1.5	0.5	1.0
**Ethnicity: Hispanic or Latin American, %**	19.9	23.2	19.3	22.2
**Age, years**	57.4 (±9.0)	56.4 (±9.2)	57.4 (±9.2)	56.5 (±8.6)
**Weight, kg**	85.0 (±17.9)	85.1 (±18.6)	85.2 (±18.0)	84.8 (±18.7)
**BMI, kg/m^2^**	30.9 (±5.1)	30.5 (±5.1)	30.9 (±4.9)	30.4 (±5.2)
**Duration of diabetes, years**	8.7 (±6.1)	9.6 (±6.1)	8.7 (±6.2)	9.6 (±6.1)
**Oral antidiabetic regimen at screening, %**				
**Metformin ± SU or glinide + DPP4-I ± AGI**	10.5	17.5	-	-
**Metformin + SU or glinide**	89.5	82.5	-	-
HbA_1c_, mmol/mol*	74	74	74	74
**HbA_1c_, %**	8.9 (±1.0)	8.9 (±0.9)	8.9 (±1.0)	8.9 (±1.0)
**FPG, mmol/L [mg/dL]**	10.1 (±2.9) [182.0 (±52.3)]	10.4 (±2.8) [187.4 (±50.5)]	10.2 (±2.7) [183.8 (±48.7)]	10.2 (±2.8) [183.8 (±50.5)]

Values are mean (±SD) unless otherwise stated.

*Calculated, not measured.

^†^One participant randomized to IDegAsp received the wrong trial drug after a dispensation error by the pharmacist. The participant was later withdrawn from the trial.

AGI, alphaglucosidase inhibitor; BMI, body mass index; DPP4-I, dipeptidyl peptidase-4 inhibitor; FPG, fasting plasma glucose; HbA_1c_, glycosylated hemoglobin; IDegIAsp, insulin degludec/insulin aspart; IGlar, insulin glargine (U100); OD, once daily; SU, sulfonylurea.

### Glycemic control

**Fig 2:**
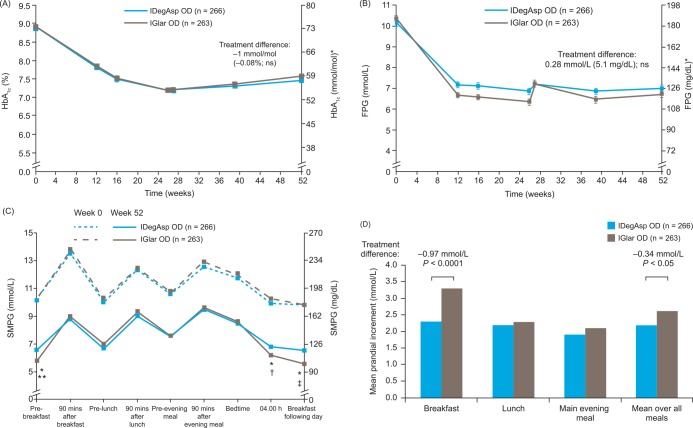
**(A) Mean HbA**_**1c**_ **over time to 52 weeks, (B) mean fasting plasma glucose (FPG) over time to 52 weeks, (C) mean self-measured blood glucose profile at baseline, Week 0 and Week 52, and (D) mean prandial increment at breakfast, lunch, main evening meal and overall at 52 weeks.** (A) and (B): Data are mean ± SEM in the full analysis set. Missing data were imputed using last observation carried forward. *Calculated, not measured. Treatment differences are derived from a least square means-based model. (C) and (D): Full analysis set. Missing data were imputed using last observation carried forward. Prandial increment is the difference between SMPG values after meal (90 min) and before meal. Comparisons: estimates adjusted for multiple covariates. *Indicate statistically significant difference at Week 52 before breakfast (p < 0.05), and at 04.00 h and at breakfast the following day (p < 0.0001). **Before breakfast: estimated treatment difference (ETD) 0.81 mmol/L; 95% CI 0.46, 1.17. ^†^At 4.00 h: ETD 0.53 mmol/L; 95% CI 0.10, 0.95. ^‡^Before breakfast the following morning: ETD 0.89 mmol/L; 95% CI 0.56, 1.23. IDegIAsp, insulin degludec/insulin aspart; HbA_1c_, glycosylated hemoglobin; IGlar, insulin glargine (U100); OD, once daily; SEM, standard error of the mean; SMPG, self-measured plasma glucose.

For both groups, mean HbA_1c_ levels decreased during the core phase and increased marginally during the extension phase (**Fig 2A**). After 26 weeks, the observed mean HbA_1c_ was 55 mmol/mol (7.2%) for IDegAsp and IGlar, corresponding to mean changes from baseline of –1.65% and –1.72%, respectively (FAS). The magnitude of the decreases in HbA_1c_ from baseline indicated that the trial had the assay sensitivity necessary to make a non-inferiority-based comparison. Mean change from baseline to Week 26 in HbA_1c_ in the FAS, IDegAsp was non-inferior to IGlar; estimated treatment difference (ETD) (IDegAsp-IGlar) 0.03% (95% confidence interval [CI] –0.14, 0.20). The results were similar for the per-protocol set. After 52 weeks, the observed mean HbA_1c_ was 59 mmol/mol (7.5%) and 60 mmol/mol (7.6%) for IDegAsp and IGlar, respectively (FAS), representing mean changes from baseline to end of the extension phase of –1.39% and –1.34%, respectively. The mean ETD (IDegAsp–IGlar) at 52 weeks was –0.08% (95% CI –0.26, 0.09), as observed in the core phase at Week 26.

The proportion of patients achieving an HbA_1c_ target < 53 mmol/mol (< 7.0%) was similar with IDegAsp and IGlar at Week 26 (45.9% and 45.6%, respectively) and at Week 52 (33.1% and 29.7%, respectively). The estimated treatment odds ratio was 0.95 (95% CI 0.66, 1.35) after 26 weeks and 1.13 (95% CI 0.77, 1.66) after 52 weeks.

Over the 52 weeks, the trend for mean FPG was similar with IDegAsp and IGlar (FAS, **Fig 2B**). Slight differences between treatment arms were observed throughout the 26-week core study phase; however, after 52 weeks of treatment, no significant difference in FPG was reported for IDegAsp and IGlar. The decrease from baseline in FPG was numerically less for IDegAsp than for IGlar both after 26 weeks (estimated mean treatment difference –3.51 mmol/L and –4.02 mmol/L, respectively) and 52 weeks (–3.50 mmol/L and –3.77 mmol/L). The ETD was 0.51 mmol/L (95% CI 0.09, 0.93) at Week 26 and 0.28 mmol/L (95% CI –0.14, 0.69) at Week 52.

Overall, 9-point SMPG profiles were similar between the two treatment groups at baseline (Week 0) and decreased during treatment (**Fig 2C**). During the 52-week period, both treatments showed similar mean 9-point SMPG profiles throughout the meals; although, significantly lower pre-breakfast SMPG values were observed with IGlar versus IDegAsp (*p* < 0.0001) at 4.00 h (*p* < 0.05) and before breakfast the following day (*p* < 0.0001) (**Fig 2C**). In contrast, at Week 52, plasma glucose levels were more stable over a 24-h period with IDegAsp versus IGlar (**Fig 2C**).

At 52 weeks, PPG increments were significantly lower with IDegAsp than with IGlar at breakfast, whereas there was no difference in PPG increments at lunch or the main evening meal. After 26 weeks, ETD (IDegAsp-IGlar) was –1.40 mmol/L (95% CI –1.92, –0.88; *p* < 0.001) at breakfast and –0.58 mmol/L (95% CI –0.90, –0.26; *p* < 0.001) at all meals. After 52 weeks, the estimated PPG mean increment across all meals was 2.37 mmol/L with IDegAsp and 2.71 mmol/L with IGlar; ETD (IDegAsp-IGlar) was –0.34 (95% CI –0.64, –0.04). PPG increment data after 52 weeks are shown in **Fig 2D**.

### Insulin dose

In both groups, the increase in insulin dose was steepest during the first few weeks of the trial. The mean total daily insulin doses were greater with IDegAsp versus IGlar at Week 26 (66 U [0.75 U/kg] *vs* 59 U [0.67 U/kg]) and at Week 52 (70 U [0.78 U/kg] *vs* 62 U [0.70 U/kg]). By the end of the trial, the majority of the IDegAsp injections were given at breakfast (59% compared with 14% at lunch and 8% in the evening), with 19% of injection time data points unknown or missing.

### Body weight

The mean increase from baseline to Week 52 in body weight was significantly greater with IDegAsp versus IGlar (4.4 kg *vs* 2.8 kg, respectively; ETD [IDegAsp−IGlar] 1.60 kg; 95% CI 0.84, 2.36; *p* < 0.0001; FAS).

### Hypoglycemic episodes

**Fig 3:**
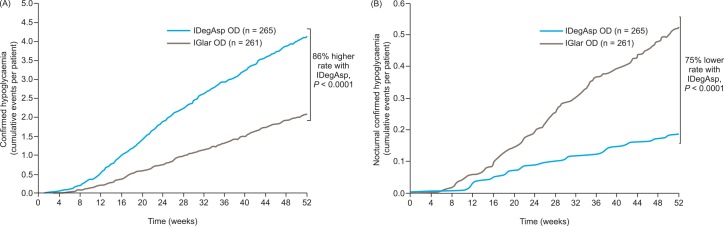
**(A) Confirmed and (B) nocturnal confirmed hypoglycemic events over 52 weeks.** Safety analysis set. Comparisons: estimates adjusted for multiple covariates. IDegIAsp, insulin degludec/insulin aspart; IGlar, insulin glargine (U100); OD, once daily.

Confirmed hypoglycemic episodes were reported in 57.7% (n = 153) and 52.1% (n = 136) of participants on IDegAsp and IGlar, respectively, over the whole treatment period. The rate of overall confirmed hypoglycemic episodes (episodes per 100 participant years of exposure [PYE]) was significantly higher with IDegAsp than IGlar (treatment ratio 1.86; 95% CI 1.42, 2.44; *p* < 0.0001; **Fig 3A**). Nocturnal confirmed hypoglycemic episodes were reported in 7.5% (n = 20) and 20.3% (n = 53) of participants on IDegAsp and IGlar, respectively. The rate of nocturnal hypoglycemic episodes was significantly lower with IDegAsp versus IGlar (treatment ratio 0.25; 95% CI 0.14, 0.47; *p* < 0.0001; **Fig 3B**). IGlar was associated with a lower rate of hypoglycemic episodes than IDegAsp after 26 weeks of treatment. The estimated rate ratio for confirmed hypoglycemia was 2.17 (95% CI 1.59, 2.94).

**Fig 4:**
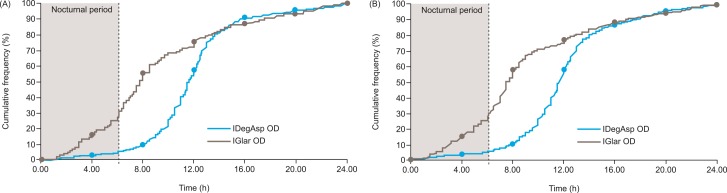
Cumulative frequency of hypoglycemia. * (A) Core phase and (B) extension phase. *Safety analysis set.IDegIAsp, insulin degludec/insulin aspart; IGlar, insulin glargine (U100); OD, once daily.

The cumulative frequency of hypoglycemia over time (**Fig 4**) shows that the majority of confirmed hypoglycemic episodes reported with IDegAsp occurred during the day (from 8.00 to 12.00) and could be attributed to the dosing at breakfast, whereas only 5% of episodes occurred at night time. In contrast, 25–30% of confirmed hypoglycemic episodes reported with IGlar occurred at night.

Overall, the rate of severe hypoglycemia was low for both IDegAsp and IGlar (0.01 events per PYE for both). Two participants (0.8%) on IDegAsp reported three severe hypoglycemic episodes and two participants (0.8%) on IGlar reported two such episodes.

### Adverse events

The percentage of participants reporting ≥ 1 AE was similar for IDegAsp (64.2%, n = 170) and IGlar (61.7%, n = 161); the majority mild-to-moderate in severity. The most commonly reported AE in both groups was nasopharyngitis. The most frequently reported serious AEs (SAEs) were hypoglycemia related. SAEs were reported in 11.3% (n = 30) with IDegAsp and 5.7% (n = 15) with IGlar. Seven SAEs were reported by six (2.3%) participants on IDegAsp considered possibly related to the investigational product (two cases of hypoglycemic unconsciousness [0.8%]; and one [0.4%] each of hyperglycemia, polymyalgia rheumatica, hemorrhagic stroke and thrombophlebitis; and one death [0.4%]). Three SAEs were reported by two (0.8%) participants (one [0.4%] each for hypoglycemic unconsciousness, hypoglycemia and respiratory distress) on IGlar.

## Discussion

The current study confirms that IDegAsp administered OD before breakfast is non-inferior to IGlar OD in terms of reducing HbA_1c_ levels from baseline to 26 and 52 weeks in insulin-naïve T2DM patients inadequately controlled on metformin. Patients on IDegAsp had a significantly higher risk of overall confirmed hypoglycemia compared with those on IGlar that may be associated with the effect of the bolus insulin in IDegAsp and the timing of administration. In contrast, patients on IDegAsp had a significantly lower risk of nocturnal-confirmed hypoglycemia compared with those on IGlar; likely the result of the flat, stable and ultra-long action profile of the basal insulin component of IDegAsp [15,16].

In the present study, participants were instructed to titrate their insulin dose according to their mean pre-breakfast glucose level. As dosing with IDegAsp provides 70% basal and 30% prandial insulin as opposed to a conventional basal insulin such as IGlar, this titration algorithm led to larger dose increments for IDegAsp compared with IGlar. The significantly greater increase in mean body weight at 52 weeks associated with IDegAsp may therefore be attributed to this effect of the protocol as participants on IDegAsp received a higher mean total insulin dose compared with those on IGlar. However, it has to be noted that in this treat-to-target trial, participants receiving IDegAsp were able to reduce their FPG levels to a similar extent as those treated with IGlar, while receiving only 70% of the basal insulin dose.

The latter interpretation is supported by findings from another 26-week, open-label, treat-to-target comparative study of IDegAsp OD versus IGlar in insulin-naïve patients, where the design allowed participants to identify their largest meal of the day when dosing IDegAsp OD and vary the time daily if their eating habits altered during the trial [21]. Dosing IDegAsp with the largest meal of the day provided superior glycemic control versus IGlar with similar FPG after 26 weeks and significantly more patients attained an HbA_1c_ target of < 7.0% (< 53 mmol/mol) without hypoglycemia in the last 12-week period [21]. Similar effects were reported in a study comparing IDeg and IGlar, where IDeg demonstrated significant improvements in FPG and rates of hypoglycemia [17,18], suggesting that the glucose-lowering effects of IDeg are preserved in IDegAsp.

In the current study, both treatments were well tolerated. The low rate of nocturnal hypoglycemia with IDegAsp reflects the flat and stable pharmacodynamic profile and ultra-long half-life of the basal IDeg component [15]. The half-life of IDeg has been reported to be approximately 25 h; twice as long as that of IGlar [22]. IDegAsp dosed with the evening main meal reduces post-dinner glucose excursions and appears to provide more stable nocturnal glucose levels than IGlar [23], which is supported by the findings of the current study. Based on the 9-point SMPG profiles, a more stable glycemic control throughout the day was observed with IDegAsp compared with IGlar.

Traditionally the use of basal-bolus regimens has been viewed as a means of treatment intensification in patients uncontrolled on basal insulin. However, IDegAsp demonstrated distinct prandial and basal glucose-lowering effects compared with biphasic insulin aspart 30 (BIAsp 30) in an OD and BID schedule when modelled at steady state [24]. In trials comparing IDegAsp BID with BIAsp 30 highlighted the improved FPG-lowering ability of IDegAsp at comparable total insulin doses [25,26] and significantly lower risk of overall confirmed and nocturnal hypoglycemia [25,27]. Furthermore, when compared with a basal-bolus regimen (IDeg OD + IAsp), IDegAsp BID demonstrated comparable glycemic control and risk of overall/nocturnal hypoglycemia with significantly less weight gain and insulin dose requirement [28].

The study design and the pharmacodynamic profile of IDegAsp probably had a strong impact on the results; specifically on the rate of overall confirmed hypoglycemia, insulin dose, FPG and prandial glucose control as well as on body weight in the IDegAsp group. The core-study protocol mandated IDegAsp to be administered at breakfast, which in some cultures is not the largest meal of the day [29]. The dietary routines of participants likely differed between participating countries (**Table 1**), which was not accounted for in the protocol. In fact, 33% of the study population originated in countries where breakfast was not the main meal of the day (Spain, India, Republic of Korea, Turkey), thus the effects of insulin treatment on glycemic control with regards to day-time hypoglycemia may vary. The bolus component in IDegAsp led to improvements in the prandial glucose increment after breakfast and the higher number of hypoglycemia episodes reported from 8.00 h to 12.00 h with IDegAsp highlight the importance of tailoring the timing of administration of IDegAsp to the individual patient’s needs, and hence the most appropriate (main) meal.

In conclusion, IDegAsp OD provides predictable, efficacious fasting and prandial glycemic control in insulin-naïve patients with T2DM in a single injection, while significantly reducing the risk of nocturnal-confirmed hypoglycemia compared with IGlar. It is important to note that although IDegAsp led to a decrease in HbA_1c_ identical to IGlar, the risk of overall hypoglycemia was significantly higher with IDegAsp OD compared with IGlar, further highlighting the importance of individualizing treatment for each patient and identifying the appropriate (largest) meal of the day with which to administer IDegAsp.

## Supporting Information

S1 Table Consort checklist. (DOC)

S1 Text Study protocol. (PDF)

## References

[1] American Diabetes Association. Standards of medical care in diabetes—2013. Diabetes Care 2013 1;36 Suppl 1:S11–S66. 10.2337/dc13-S011 23264422 PMC3537269

[2] Butler PC, Meier JJ, Butler AE, Bhushan A. The replication of β cells in normal physiology, in disease and for therapy. Nat Clin Pract Endocrinol Metab. 2007 11;3(11):758–768. 10.1038/ncpendmet0647 17955017

[3] Inzucchi SE, Bergenstal RM, Buse JB, Diamant M, Ferrannini E, Nauck M, et al; American Diabetes Association (ADA); European Association for the Study of Diabetes (EASD). Management of hyperglycemia in type 2 diabetes: a patient-centered approach: position statement of the American Diabetes Association (ADA) and the European Association for the Study of Diabetes (EASD). Diabetes Care. 2012 6;35(6):1364–1379. 10.2337/dc12-0413 22517736 PMC3357214

[4] Inzucchi SE, Bergenstal RM, Buse JB, Diamant M, Ferrannini E, Nauck M, et al; Management of hyperglycemia in type 2 diabetes, 2015: a patient-centered approach: update to a position statement of the American Diabetes Association and the European Association for the Study of Diabetes. Diabetes Care. 2015 1;38(1):140–149. 10.2337/dc14-2441 25538310

[5] Peyrot M, Rubin RR, Lauritzen T, Skovlund SE, Snoek FJ, Matthews DR, et al Resistance to insulin therapy among patients and providers: results of the cross-national Diabetes Attitudes, Wishes, and Needs (DAWN) study. Diabetes Care. 2005 11;28(11):2673–2679. 16249538 10.2337/diacare.28.11.2673

[6] Brod M, Alolga SL, Meneghini L. Barriers to initiating insulin in type 2 diabetes patients: development of a new patient education tool to address myths, misconceptions and clinical realities. Patient. 2014;7(4):437–450. 10.1007/s40271-014-0068-x 24958464 PMC4240906

[7] Leiter L, Yale J, Chiasson J, Harris S, Kleinstiver P, Sauriol L. Assessment of the impact of fear of hypoglycemic episodes on glycemic and hypoglycemia management. Can J Diabetes. 2005;29(3):186–192.

[8] Raccah D, Bretzel RG, Owens D, Riddle M. When basal insulin therapy in type 2 diabetes mellitus is not enough—what next? Diabetes Metab Res Rev. 2007 5;23(4):257–264. 10.1002/dmrr.733 17315242

[9] Raccah D. Options for the intensification of insulin therapy when basal insulin is not enough in type 2 diabetes mellitus. Diabetes Obes Metab. 2008 7;10(Suppl. 2):76–82. 10.1111/j.1463-1326.2008.00846.x 18577159

[10] Giugliano D, Maiorino MI, Bellastella G, Chiodini P, Esposito K. Treatment regimens with insulin analogues and haemoglobin A1c target of <7% in type 2 diabetes: a systematic review. Diabetes Res Clin Pract. 2011 4;92(1):1–10. 10.1016/j.diabres.2010.08.006 20822821

[11] Lasserson DS, Glasziou P, Perera R, Holman RR, Farmer AJ. Optimal insulin regimens in type 2 diabetes mellitus: Systematic review and meta-analyses. Diabetologia. 2009 10;52(10):1990–2000. 10.1007/s00125-009-1468-7 19644668

[12] Pontiroli AE, Miele L, Morabito A. Metabolic control and risk of hypoglycaemia during the first year of intensive insulin treatment in type 2 diabetes: Systematic review and meta-analysis. Diabetes Obes Metab. 2012 5;14(5):433–446. 10.1111/j.1463-1326.2011.01543.x 22142056

[13] Khunti K, Caputo S, Damci T, Dzida GJ, Ji Q, Kaiser M et al; SOLVE Study Group. The safety and efficacy of adding once-daily insulin detemir to oral hypoglycaemic agents in patients with type 2 diabetes in a clinical practice setting in 10 countries. Diabetes Obes Metab. 2012 12;14(12):1129–1136. 10.1111/j.1463-1326.2012.01665.x 22830956

[14] Evans M, Schumm-Draeger PM, Vora J, King AB. A review of modern insulin analogue pharmacokinetic and pharmacodynamic profiles in type 2 diabetes: improvements and limitations. Diabetes Obes Metab. 2011 8;13(8):677–684. 10.1111/j.1463-1326.2011.01395.x 21410860 PMC3380549

[15] Heise T, Nosek L, Bøttcher SG, Hastrup H, Haahr H. Ultra-long-acting insulin degludec has a flat and stable glucose-lowering effect in type 2 diabetes. Diabetes Obes Metab. 2012 10;14(10):944–950. 10.1111/j.1463-1326.2012.01638.x 22726241

[16] Heise T, Hermanski L, Nosek L, Feldman A, Rasmussen S, Haahr H. Insulin degludec: four times lower pharmacodynamic variability than insulin glargine under steady-state conditions in type 1 diabetes. Diabetes Obes Metab. 2012 9;14(9):859–864. 10.1111/j.1463-1326.2012.01627.x 22594461

[17] Zinman B, Philis-Tsimikas A, Cariou B, Handelsman Y, Rodbard HW, Johansen T, et al Insulin degludec versus insulin glargine in insulin-naive patients with type 2 diabetes: a 1-year, randomized, treat-to-target trial (BEGIN Once Long). Diabetes Care. 2012 12;35(12):2464–2471. 10.2337/dc12-1205 23043166 PMC3507614

[18] Rodbard HW, Cariou B, Zinman B, Handelsman Y, Philis-Tsimikas A, Skjøth TV, et al Comparison of insulin degludec with insulin glargine in insulin-naive subjects with type 2 diabetes: a 2-year randomized, treat-to-target trial. Diabet Med. 2013 11;30(11):1298–304. 10.1111/dme.12303 23952326 PMC4208679

[19] Havelund S. “Insulin degludec (ideg) and insulin aspart (iasp) can be coformulated such that the formation of ideg multi-hexamers and iasp monomers is retained upon subcutaneous injection.” American Diabetes Association, 21–25 June 2013, Chicago, USA. 2013.

[20] Lantus®. Insuman Rapid (100 IU/ml) (sanofi-aventis). http://www.ema.europa.eu/docs/en_GB/document_library/EPAR_-_Product_Information/human/000284/WC500036082.pdf

[21] Onishi Y, Ono Y, Rabøl R, Endahl L, Nakamura S. Superior glycaemic control with once-daily insulin degludec/insulin aspart versus insulin glargine in Japanese adults with type 2 diabetes inadequately controlled with oral drugs: a randomized, controlled phase 3 trial. Diabetes Obes Metab. 2013 9;15(9):826–832. 10.1111/dom.12097 23557077

[22] Heise T, Hovelmann U, Nosek L, Bøttcher S, Granhall C, Haahr H, et al Insulin degludec: two-fold longer half-life and a more consistent pharmacokinetic profile than insulin glargine. Endocrine Abstracts 2011;28:P188.

[23] Liebl A, Davidson J, Mersebach H, Dykiel P, Tack CJ, Heise T. A novel insulin combination of insulin degludec and insulin aspart achieves a more stable overnight glucose profile than insulin glargine: results from continuous glucose monitoring in a proof-of-concept trial. J Diabetes Sci Technol. 2013 9;7(5):1328–1336. 24124961 10.1177/193229681300700524 PMC3876378

[24] Heise T, Nosek L, Roepstorff C, Chenji S, Klein O, Haahr H. Distinct prandial and basal glucose-lowering effects of insulin degludec/insulin aspart (idegasp) at steady state in subjects with type 1 diabetes mellitus. Diabetes Ther. 2014 6;5(1):255–265. 10.1007/s13300-014-0070-2 24888255 PMC4065302

[25] Fulcher GR, Christiansen JS, Bantwal G, Polaszewska-Muszynska M, Mersebach H, Andersen TH, et al; BOOST: Intensify Premix I Investigators. Comparison of insulin degludec/insulin aspart and biphasic insulin aspart 30 in uncontrolled, insulin-treated type 2 diabetes: a phase 3a, randomized, treat-to-target trial. Diabetes Care. 2014 8;37(8):2084–2090. 10.2337/dc13-2908 24812432

[26] Kaneko S, Chow F, Choi DS, Taneda S, Hirao K, Park Y et al; BOOST: Intensify All Trial Investigators. Insulin degludec/insulin aspart versus biphasic insulin aspart 30 in Asian patients with type 2 diabetes inadequately controlled on basal or pre-/self-mixed insulin: a 26-week, randomized, treat-to-target trial. Diabetes Res Clin Pract. 2015 1;107(1):139–147. 10.1016/j.diabres.2014.09.026 25498130

[27] Vaag A, Christiansen JS, Niskanen L et al Lower rates of overall, nocturnal and severe hypoglycaemia during maintenance treatment with IDegAsp vs biphasic insulin aspart 30 in patients with type 2 diabetes mellitus: a meta-analysis. Diabetologia. 2013;56:S83.

[28] Rodbard HW, Cariou B, Pieber TR, Endahl LA, Zacho J, Cooper JG. Treatment intensification with an insulin degludec (IDeg)/insulin aspart (IAsp) co-formulation twice daily compared with basal IDeg and prandial IAsp in type 2 diabetes: a randomized, controlled phase III trial. Diabetes Obes Metab. 2016 3;18(3):274–280. 10.1111/dom.12609 26592732 PMC5066701

[29] Garaulet M, Gómez-Abellán P, Alburquerque-Béjar JJ, Lee YC, Ordovás JM, Scheer FA. Timing of food intake predicts weight loss effectiveness. Int J Obes. 2013 4;37(4):604–611. 10.1038/ijo.2012.229 PMC3756673 23357955

