# Supplementary material for: Efficacy and Safety of Once-Daily Insulin Degludec/Insulin Aspart versus Insulin Glargine (U100) for 52 Weeks in Insulin-Naïve Patients with Type 2 Diabetes: A Randomized Controlled Trial
Source: PLoS One. 2016 Oct 19;11(10):e0163350. doi: 10.1371/journal.pone.0163350 (PMC5070831; doi:10.1371/journal.pone.0163350)
Supplement: S1 Text — (PDF) [file pone.0163350.s002.pdf]

Clinical Trial Report

Trial ID: NN5401-3726

BOOST™: START 1

An Extension Trial Comparing Safety and Efficacy of NN5401 with Insulin Glargine in Subjects with Type 2 Diabetes

A 26-week, multinational, multi-centre, open-labelled, two-arm, parallel, treat-to-target extension trial comparing safety and efficacy of NN5401 once daily (OD) plus metformin vs. insulin glargine OD plus metformin in subjects with type 2 diabetes

This report contains the results after 52 weeks treatment (26 weeks in the main trial NN5401-3590 and 26 weeks in the present extension trial NN5401-3726)

This confidential document is the property of Novo Nordisk. No unpublished information contained herein may be disclosed without prior written approval from Novo Nordisk. Access to this document must be restricted to relevant parties.

1 Title Page

Title of Trial	A 26-week, multinational, multi-centre, open-labelled, two-arm, parallel, treat-to-target extension trial comparing safety and efficacy of NN5401 once daily (OD) plus metformin vs. insulin glargine OD plus metformin in subjects with type 2 diabetes
Trial ID	NN5401-3726
Development Phase	Phase 3a
Trial Registration ID no.	NCT01169766
IND Number (US only)	IND 73,198
EudraCT Number	2009-015839-33
Japanese Trial Number	Not Applicable
Generic Name	Insulin degludec/insulin aspart (IDegAsp)
Indication	Diabetes mellitus
Investigator(s)	There were 76 principal investigators. One principal investigator was appointed for each trial site. Ajay Kumar, MD was appointed as a signatory investigator: Diabetes Care & Research Centre, GC1B, Near Over Bridge, Kankerbagh, Patna 800020, India.
Trial Site(s)	The trial was conducted at 76 sites in 8 countries: Austria (4 sites), India (7 sites), Republic of Korea (5 sites), Poland (6 sites), Russia (8 sites), Spain (9 sites), Turkey (5 sites), and United States (32 sites). These sites enrolled subjects.
Trial Initiated	26 July 2010
Trial Completed	4 May 2011
Sponsor	Novo Nordisk A/S, Denmark
International Medical Officer	Trine Vang Skjøth, MD
International Trial Manager	Julie Solbjerg Appel, MSc
Local Trial Manager(s)	Judith Muehlhauser (Austria), Avik Kumar Ghosh (India). JungEun Lee (Republic of Korea), Magdalena Jawoszek (Poland), Elena Bogdanova (Russia), Olga María García (Spain), Erce Kalabalikoglu (Turkey), Allison Werst (United States).
Trial Statistician	Pei-Ling Chu, PhD, Novo Nordisk Inc., USA
Trial Medical Writer	Nicole D. Hunt, PhD/ Lisa Bonk, PharmD, MBA
Report Date(s)	20 October 2011

The trial was conducted in accordance with the Declaration of Helsinki[1] and ICH Good Clinical Practice.[2]

2 Synopsis

Please refer to separate document.

3 Table of Contents for the Individual Clinical Study

Page

1 Title Page 2
2 Synopsis 3
3 Table of Contents for the Individual Clinical Study Report 4
4 List of Abbreviations and Definition of Terms 25
5 Ethics 28
5.1 Independent Ethics Committee (IEC) or Institutional Review Board (IRB) 28
5.2 Ethical Conduct of the Trial 28
5.3 Subject Information and Consent 28
6 Investigators and Trial Administrative Structure 29
7 Introduction 33
7.1 Therapeutic Area 33
7.2 Insulin Degludec/Insulin Aspart 33
7.3 Insulin Glargine 34
7.4 Rationale for the Trial 34
8 Trial Objectives 36
9 Investigational Plan 37
9.1 Overall Trial Design and Plan: Description 37
9.2 Discussionof Trial Design, Including the Choice of Control Groups 39
9.3 Selection of Trial Population 40
9.3.1 Inclusion Criteria 40
9.3.2 Exclusion Criteria 41
9.3.3 Removal of Subjects from Therapy and Assessment 44
9.4 Treatments 46
9.4.1 Treatments Administered 46
9.4.2 Identity of Investigational Products 47
9.4.3 Method of Assigning Subjects to Treatment Groups 49
9.4.4 Selection of Doses in the Trial 49
9.4.5 Selection and Timing of Dose for Each Subject 49
9.4.6 Blinding 50
9.4.7 Prior and Concomitant Therapy 51
9.4.8 Treatment Compliance 51
9.5 Efficacy and Safety Variables 52
9.5.1 Efficacy and Safety Measurements Assessed and Flow Chart 52
9.5.1.1 Flow Chart 52
9.5.1.2 Efficacy Assessments 60
9.5.1.3 Safety Assessments 62
9.5.1.4 Assessments of Demographic and other Baseline Characteristics 72
9.5.2 Appropriateness of Measurements 73
9.5.3 Primary Efficacy Variable(s) 73
9.6 Data Quality Assurance 73
9.7 Statistical Methods and Determination of Sample Size 73

9.7.1 General Considerations 73
9.7.2 Endpoints in Relation to the Objectives and Hypotheses 74
9.7.3 Definition of Analysis Sets 75
9.7.4 Statistical Analyses 76
9.7.4.1 Primary Endpoints 76
9.7.4.2 Secondary Endpoints 80
9.7.5 Interim Analyses 83
9.7.6 Sequential Safety Analysis/Safety Monitoring 83
9.7.7 Exploratory Statistical Analyses for Pharmacogenetics and Biomarkers 83
9.7.8 PK and PD Modelling 83
9.7.9 Health Economics and/or Patient Reported Outcomes 83
9.7.10 Determination of Sample Size 83
9.8 Changes in the Conduct of the Study or Planned Analyses 84
9.8.1 Protocol Amendments and General Procedural Deviations 84
9.8.2 Changes to the Statistical Analysis Planned in the Protocol 85
10 Trial Subjects 86
10.1 Disposition of Subjects 86
10.2 Protocol Deviations 89
10.2.1 Important Protocol Deviations 89
10.2.2 Important Deviations at Trial Site Level 89
10.2.2.1 Important Deviations at Trial Site Level- Main Trial 89
10.2.2.2 Important Deviations at Trial Site Level-Main Trial, after Finalisation of CTR.... 89
10.2.2.3 Important Deviations at Trial Site Level- Extension Trial 89
10.2.3 Important Deviations at Subject Level 90
10.2.3.1 Important Deviations at Subject Level- Main Trial 90
10.2.3.2 Important Deviations at Subject Level- Main Trial, after Finalisation of CTR...... 94
10.2.3.3 Important Deviations at Subject Level- Extension Trial 94
11 Efficacy Evaluation 96
11.1 Data Sets Analysed 96
11.2 Demographic and Other Baseline Characteristics 97
11.3 Measurements of Treatment Compliance 105
11.4 Efficacy Results 105
11.4.1 Analysis of Efficacy 105
11.4.1.1 HbA1c 105
11.4.1.2 Responders for HbA1c 109
11.4.1.3 Fasting Plasma Glucose 113
11.4.1.4 9-point Self-measured Plasma Glucose Profiles 114
11.4.1.5 SMPG Values used for Dose Adjustments 117
11.4.2 PK and PD Modelling 119
11.4.3 Statistical/Analytical Issues 119
11.4.3.1 Handling of Dropouts or Missing Data 119
11.4.4 Efficacy Conclusions 120
12 Safety Evaluation 122
12.1 Extent of Exposure 122
12.1.1 Exposure to Trial Products 122

12.1.2 Dosing During the Trial 123
12.1.2.1 Insulin Regimen and Dosing Time 123
12.1.2.2 Insulin Dose 123
12.1.2.3 Insulin Dose Ratio at End of Trial 125
12.1.2.4 Difference between Titration Algorithm Dose and Prescribed Dose 126
12.1.2.5 Difference between Prescribed Dose and Actual Dose 126
12.2 Adverse Events 126
12.2.1 Brief Summary of Adverse Events 126
12.2.2 Analysis of Adverse Events 129
12.2.2.1 All Adverse Events 129
12.2.2.2 Adverse Events by Relationship to Investigational Product (IDegAsp or IGlar) ... 132
12.2.2.3 Adverse Events by Relationship to Insulin Delivery Device 135
12.2.2.4 Adverse Events by Severity 135
12.3 Deaths, Other Serious Adverse Events and Other Significant Adverse Events 135
12.3.1 Deaths 135
12.3.2 Other Serious Adverse Events 137
12.3.2.1 All Serious Adverse Events 137
12.3.2.2 Serious Adverse Events by Relationship 142
12.3.3 Other Significant Adverse Events 145
12.3.3.1 Adverse Events Leading to Withdrawal 145
12.3.3.2 Adverse Events Leading to Dose Reduction in Investigational Product 147
12.3.3.3 Medical Events of Special Interest 147
12.4 Hypoglycaemia 148
12.4.1 Introduction to Hypoglycaemic Episodes 148
12.4.2 Confirmed Hypoglycaemic Episodes 148
12.4.3 Severe Hypoglycaemia 151
12.4.4 Nocturnal Hypoglycaemic Episodes 152
12.4.4.1 Nocturnal Confirmed Hypoglycaemic Episodes 152
12.4.4.2 Nocturnal Severe Hypoglycaemic Episodes 154
12.4.5 Hypoglycaemic Episodes according to the ADA Classification 154
12.5 Clinical Laboratory Evaluation 154
12.5.1 Evaluation of Insulin Antibodies 154
12.5.1.1 Cross-reacting Insulin Antibodies 154
12.5.1.2 Specific Insulin Antibodies 157
12.5.1.3 Total Insulin Antibodies 157
12.5.2 Evaluation of Each Laboratory Parameter 158
12.5.2.1 Laboratory Values Over Time 158
12.5.2.2 Individual Subject Changes 158
12.5.2.3 Individual Clinically Significant Abnormalities 158
12.6 Vital Signs, Physical Findings and Other Observations Related to Safety 159
12.6.1 Vital Signs 159
12.6.2 Physical Examination 160
12.6.3 ECG 160
12.6.4 Fundoscopy / Fundusphotography 160
12.6.5 Body Weight and Body Mass Index 161

12.7 Safety Conclusions 162
13 Discussion and Overall Conclusions 164
14 Tables, Figures and Graph Referred to but not Included in the Text 169
14.1 Demographic Data 169
14.1.1: Subject Disposition - Summary 170
14.1.2: Subject Disposition by Country - Summary 172
14.1.3: Time to Discontinuation (Weeks) - Full Analysis Set 175
14.1.4: Time to Discontinuation (Weeks) - All Non-Completers - Full Analysis Set 176
14.1.5: Subject Disposition by Week - Summary - Full Analysis Set 177
14.1.6: Reasons for Discontinuation from Week 12 and onwards - Full Analysis Set 178
14.1.7: Demographics and Baseline Characteristics - Summary - Full Analysis Set 179
14.1.8: Demographics and Baseline Characteristics - Summary - Extension Trial Set 180
14.1.9: Baseline and Diabetes Characteristics – Descriptive Statistics - Full Analysis Set 181
14.1.10: Baseline and Diabetes Characteristics - Descriptive Statistics - Extension Trial Set 182
14.1.11: Anti-diabetic Treatment Strata at Screening – Full Analysis Set 183
14.1.12: OAD at Screening - Summary - Full Analysis Set 184
14.1.13: Diabetes Complications at Screening - Summary - Full Analysis Set 186
14.1.14: Concomitant Illness at Screening - Summary - Full Analysis Set 187
14.1.15: Concomitant Medication at Screening – Summary - Full Analysis Set 199
14.1.16: Concomitant Medication Started after Screening – Summary - Full Analysis Set 226
14.1.17: Mono and Combination OAD Therapies at Screening - Summary - Full Analysis Set 267
14.2 Efficacy Data 268
14.2.1: Exposure - Descriptive Statistics - Safety Analysis Set 269
14.2.2: Exposure – Descriptive Statistics - Extension Trial Set 270
14.2.3: Exposure by Treatment Week - Summary - Safety Analysis Set 271
14.2.4: Exposure by Treatment Week - Summary - Extension Trial Set 272
14.2.5: Exposure by Sex and Treatment Week - Summary - Safety Analysis Set 273
14.2.6: Exposure by Race and Treatment Week - Summary - Safety Analysis Set 274
14.2.7: Exposure by Ethnicity and Treatment Week - Summary - Safety Analysis Set 278
14.2.8: Exposure by Age Group (Adults and Elderly) and Treatment Week - Summary - Safety Analysis Set 280
14.2.9: Exposure - Empirical Distribution Plot - Safety Analysis Set 281
14.2.10: IDegAsp/Comparator Insulin Dose (Actual) in Units by Treatment Week - Descriptive Statistics - Safety Analysis Set 282
14.2.11: IDegAsp/Comparator Insulin Dose (Actual) in Units by Treatment Week - Descriptive Statistics - Extension Trial Set 286
14.2.12: IDegAsp/Comparator Insulin Dose (Prescribed) in Units by Treatment Week - Descriptive Statistics - Safety Analysis Set 290
14.2.13: IDegAsp/Comparator Insulin Dose (Actual) in Units/kg by Treatment Week - Descriptive Statistics - Safety Analysis Set 294
14.2.14: IDegAsp/Comparator Insulin Dose (Actual) in Units/kg by Treatment Week - Descriptive Statistics - Extension Trial Set 298
14.2.15: Daily IDegAsp/Comparator Insulin Dose (Actual) in Units by Treatment Week - Mean Plot - Safety Analysis Set 302

14.2.16: Daily IDegAsp/Comparator Insulin Dose (Actual) in Units by Treatment Week - Mean Plot - Extension Trial Set 303
14.2.17: Daily IDegAsp/Comparator (Prescribed) in Units by Treatment Week - Mean Plot - Safety Analysis Set 304
14.2.18: Daily IDegAsp/Comparator (Actual) in Units/kg by Treatment Week - Mean Plot - Safety Analysis Set 305
14.2.19: Daily IDegAsp/Comparator (Actual) in Units/kg by Treatment Week - Mean Plot - Extension Trial Set 306
14.2.20: Daily IDegAsp OD Dose (Actual) in Units/kg by Treatment Week - Box Plot - Safety Analysis Set 307
14.2.21: Daily IGlar Dose (Actual) in Units/kg by Treatment Week - Box Plot - Safety Analysis Set 308
14.2.22: Daily IDegAsp OD Dose (Prescribed)in Units by Treatment Week - Box Plot - Safety Analysis Set 309
14.2.23: Daily IGlar Dose (Prescribed) in Units by Treatment Week - Box Plot - Safety Analysis Set 310
14.2.24: Daily IDegAsp/Comparator Insulin Dose (Actual) in Units after 52 Weeks of Treatment - Empirical Distribution Plot - Safety Analysis Set 311
14.2.25: Daily IDegAsp/Comparator Insulin Dose (Prescribed) in Units after 52 Weeks of Treatment - Empirical Distribution Plot - Safety Analysis Set 312
14.2.26: Daily IDegAsp/Comparator Insulin Dose (Actual) in Units/kg after 52 Weeks of Treatment - Empirical Distribution Plot - Safety Analysis Set 313
14.2.27: IDegAsp OD/IGlar OD Dose Ratio (Actual) after 52 Weeks of Treatment - Summary – Safety Analysis Set 313
14.2.28: Prescribed minus Actual Daily IDegAsp/Comparator Insulin Dose in Units by Treatment Week - Descriptive Statistics - Safety Analysis Set 314
14.2.29: Prescribed minus Titration Algorithm Daily IDegAsp/Comparator Insulin Dose in Units by Treatment Week - Descriptive Statistics - Safety Analysis Set 319
14.2.30: Daily IDegAsp OD Dose in Units by Treatment Week - Prescribed, Actual and Titration Algorithm Dose - Mean Plot - Safety Analysis Set 323
14.2.31: Daily IGlar Dose in Units by Treatment Week - Prescribed, Actual and Titration Algorithm Dose – Mean Plot - Safety Analysis Set 324
14.2.32: Injection Time by Treatment Week in the Extension Period - Descriptive Statistics -Extension Trial Set 325
14.2.33: HbA1c (%) by Treatment Week – Descriptive Statistics - Full Analysis Set 329
14.2.34: HbA1c (%) by Treatment Week – Descriptive Statistics - Extension Trial Set 331
14.2.35: HbA1c (%) by Treatment Week – Change from Baseline - Descriptive Statistics - Full Analysis Set 333
14.2.36: HbA1c (%) by Treatment Week – Change from Baseline - Descriptive Statistics - Extension Trial Set 334
14.2.37: HbA1c (%) after 52 Weeks of Treatment - Statistical Analysis - Full Analysis Set... 335
14.2.38: HbA1c (%) after 52 Weeks of Treatment - Statistical Analysis - Extension Trial Set 336
14.2.39: HbA1c (%) after 52 Weeks of Treatment - Statistical Sensitivity Analysis - PP Analysis Set 337
14.2.40: HbA1c (%) after 52 Weeks of Treatment - Statistical Sensitivity Analysis - Simple Model - Full Analysis Set 338
14.2.41: HbA1c (%) after 52 Weeks of Treatment - Statistical Sensitivity Analysis - Repeated Measurement Model - Full Analysis Set 339

14.2.42: HbA1c (%) by Treatment Week – Mean Plot - Full Analysis Set 339
14.2.43: HbA1c (%) by Treatment Week - Mean Plot - Extension Trial Set........................... 340
14.2.44: HbA1c (%) by Treatment Week – Change from Baseline - Mean Plot - Full Analysis Set 341
14.2.45: HbA1c (%) by Treatment Week – Change from Baseline - Mean Plot - Extension Trial Set... 342
14.2.46: HbA1c (%) by Treatment Week – Change from Baseline - Mean Plot - PP Analysis Set 343
14.2.47: HbA1c (%) by Treatment Week – Box Plot - Full Analysis Set................................ 344
14.2.48: HbA1c (%) after 52 Weeks of Treatment – Empirical Distribution Plot - Full Analysis Set 345
14.2.49: HbA1c (%) after 52 Weeks of Treatment – Change from Baseline - Empirical Distribution Plot - Full Analysis Set.. 346
14.2.50: Responder for HbA1c by Treatment Week – Summary - Full Analysis Set............... 347
14.2.51: Responder for HbA1c at End of Trial - Statistical Analysis - Full Analysis Set......... 349
14.2.52: Responder for HbA1c at End of Trial Without Hypoglycaemia – Summary - Full Analysis Set 350
14.2.53: Responder for HbA1c at End of Trial Without Hypoglycaemia - Statistical Analysis - Full Analysis Set... 354
14.2.54: Fasting Plasma Glucose (mmol/L) by Treatment Week – Descriptive Statistics - Full Analysis Set... 355
14.2.55: Fasting Plasma Glucose (mmol/L) by Treatment Week – Change from Baseline - Descriptive Statistics - Full Analysis Set.. 356
14.2.56: Fasting Plasma Glucose (mmol/L) after 52 Weeks of Treatment - Statistical Analysis - Full Analysis Set... 357
14.2.57: Fasting Plasma Glucose (mmol/L) by Treatment Week – Mean Plot - Full Analysis Set 358
14.2.58: Fasting Plasma Glucose (mmol/L) by Treatment Week – Mean Plot - Extension Trial Set... 359
14.2.59: Fasting Plasma Glucose (mmol/L) by Treatment Week – Change from Baseline – Mean Plot - Full Analysis Set 360
14.2.60: Fasting Plasma Glucose (mmol/L) by Treatment Week - Box Plot - Full Analysis Set 361
14.2.61: Fasting Plasma Glucose (mmol/L) after 52 Weeks of Treatment - Empirical Distribution Plot - Full Analysis Set.. 362
14.2.62: Fasting Plasma Glucose (mmol/L) after 52 Weeks of Treatment – Change from Baseline - Empirical Distribution Plot - Full Analysis Set 363
14.2.63: 9-point Self Measured Plasma Glucose Profile (mmol/L) by Treatment Week – Descriptive Statistics - Full Analysis Set.. 364
14.2.64: 9-point Self Measured Plasma Glucose Profile (mmol/L) after 52 Weeks of Treatment - Statistical Analysis - Full Analysis Set 366
14.2.65: 9-point Self Measured Plasma Glucose Profile (mmol/L) by TreatmentWeeks - Mean Plot - IDegAsp OD - Full Analysis Set... 368
14.2.66: 9-point Self Measured Plasma Glucose Profile (mmol/L) by Treatment Weeks - Mean Plot - IGlar OD - Full Analysis Set... 369
14.2.67: 9-point Self Measured Plasma Glucose Profile (mmol/L) at Baseline - Mean Plot - Full Analysis Set... 370

14.2.68: 9-point Self Measured Plasma Glucose Profile (mmol/L) after 12 Weeks - Mean Plot - Full Analysis Set ... 371
14.2.69: 9-point Self Measured Plasma Glucose Profile (mmol/L) after 16 Weeks - Mean Plot - Full Analysis Set ... 372
14.2.70: 9-point Self Measured Plasma Glucose Profile (mmol/L) after 26 Weeks - Mean Plot - Full Analysis Set ... 373
14.2.71: 9-point Self Measured Plasma Glucose Profile (mmol/L) after 39 Weeks - Mean Plot - Full Analysis Set ... 374
14.2.72: 9-point Self Measured Plasma Glucose Profile (mmol/L) after 52 Weeks - Mean Plot - Full Analysis Set ... 375
14.2.73: Mean of 9-point Self Measured Plasma Glucose Profile (mmol/L) by Treatment Week – Descriptive Statistics - Full Analysis Set ... 376
14.2.74: Mean of 9-point Self Measured Plasma Glucose Profile (mmol/L) by Treatment Week – Change from Baseline - Descriptive Statistics - Full Analysis Set ... 377
14.2.75: Mean of 9-point Self Measured Plasma Glucose Profile (mmol/L) after 52 Weeks of Treatment - Statistical Analysis - Full Analysis Set ... 378
14.2.76: Mean of 9-point Self Measured Plasma Glucose Profile (mmol/L) by Treatment Week – Mean Plot - Full Analysis Set ... 379
14.2.77: Mean of 9-point Self Measured Plasma Glucose Profile (mmol/L) by Treatment Week – Change from Baseline – Mean Plot - Full Analysis Set ... 380
14.2.78: Mean of 9-point Self Measured Plasma Glucose Profile (mmol/L) after 52 Weeks of Treatment - Empirical Distribution Plot - Full Analysis Set ... 381
14.2.79: Mean of 9-point Self Measured Plasma Glucose Profile (mmol/L) after 52 Weeks of Treatment – Change from Baseline - Empirical Distribution Plot - Full Analysis Set... 382
14.2.80: Fluctuation in 9-point Self Measured Plasma Glucose Profile (mmol/L) by Treatment Week - Descriptive Statistics - Full Analysis Set ... 383
14.2.81: Fluctuation in 9-point Self Measured Plasma Glucose Profile (mmol/L) after 52 Weeks of Treatment - Statistical Analysis - Full Analysis Set ... 385
14.2.82: Fluctuation in 9-point Self Measured Plasma Glucose Profile (mmol/L) by Treatment Week – Mean Plot - Full Analysis Set ... 386
14.2.83: Fluctuation in 9-point Self Measured Plasma Glucose Profile (mmol/L) by Treatment Week – Change from Baseline – Mean Plot - Full Analysis Set ... 387
14.2.84: Fluctuation in 9-point Self Measured Plasma Glucose Profile (mmol/L) after 52 Weeks of Treatment - Empirical Distribution Plot - Full Analysis Set ... 388
14.2.85: Fluctuation in 9-point Self Measured Plasma Glucose Profile (mmol/L) after 52 Weeks of Treatment – Change from Baseline - Empirical Distribution Plot - Full Analysis Set ... 389
14.2.86: Prandial Increment by Treatment Week - 9-point Self Measured Plasma Glucose Profile (mmol/L) – Descriptive Statistics - Full Analysis Set ... 390
14.2.87: Prandial Increment by Treatment Week – Change from Baseline - 9-point Self Measured Plasma Glucose Profile (mmol/L) - Descriptive Statistics - Full Analysis Set ... 394
14.2.88: Breakfast Increment after 52 Weeks of Treatment - 9-point Self Measured Plasma Glucose Profile (mmol/L) - Statistical Analysis - Full Analysis Set ... 398
14.2.89: Prandial Increment after 52 Weeks of Treatment - 9-point Self Measured Plasma Glucose Profile (mmol/L) - Statistical Analysis - Full Analysis Set ... 399
14.2.90: Breakfast Increment by Treatment Week -9-point Self Measured Plasma Glucose Profile (mmol/L)- Mean Plot - Full Analysis Set ... 400

14.2.91: Breakfast Increment by Treatment Week - Change from Baseline - 9-point Self Measured Plasma Glucose Profile (mmol/L)- Mean Plot - Full Analysis Set 401
14.2.92: Breakfast Increment after 52 Weeks of Treatment - 9-point Self Measured Plasma Glucose Profile (mmol/L) - Empirical Distribution Plot - Full Analysis Set 402
14.2.93: Breakfast Increment after 52 Weeks of Treatment - Change from Baseline - 9-point Self Measured Plasma Glucose Profile (mmol/L) - Empirical Distribution Plot - Full Analysis Set 403
14.2.94: Lunch Increment by Treatment Week - 9-point Self Measured Plasma Glucose Profile (mmol/L) - Mean Plot - Full Analysis Set 404
14.2.95: Lunch Increment by Treatment Week - Change from Baseline - 9-point Self Measured Plasma Glucose Profile (mmol/L) - Mean Plot - Full Analysis Set 405
14.2.96: Lunch Increment after 52 Weeks of Treatment - 9-point Self Measured Plasma Glucose Profile (mmol/L) - Empirical Distribution Plot - Full Analysis Set 406
14.2.97: Lunch Increment after 52 Weeks of Treatment - Change from Baseline - 9-point Self Measured Plasma Glucose Profile (mmol/L) - Empirical Distribution Plot - Full Analysis Set 407
14.2.98: Main Evening Meal Increment by Treatment Week - 9-point Self Measured Plasma Glucose Profile (mmol/L)– Mean Plot - Full Analysis Set 408
14.2.99: Main Evening Meal Increment by Treatment Week - Change from Baseline - 9-point Self Measured Plasma Glucose Profile (mmol/L)– Mean Plot - Full Analysis Set... 409
14.2.100: Main Evening Meal Increment after 52 Weeks of Treatment - 9-point Self Measured Plasma Glucose Profile (mmol/L) - Empirical Distribution Plot - Full Analysis Set 410
14.2.101: Main Evening Meal Increment after 52 Weeks of Treatment - Change from Baseline - 9-point Self Measured Plasma Glucose Profile (mmol/L) - Empirical Distribution Plot - Full Analysis Set... 411
14.2.102: All Meals Increment by Treatment Week - 9-point Self Measured Plasma Glucose Profile (mmol/L)– Mean Plot - Full Analysis Set... 412
14.2.103: All Meals Increment by Treatment Week - Change from Baseline - 9-point Self Measured Plasma Glucose Profile (mmol/L)- Mean Plot - Full Analysis Set 413
14.2.104: All Meals Increment after 52 Weeks of Treatment - 9-point Self Measured Plasma Glucose Profile (mmol/L) - Empirical Distribution Plot -Full Analysis Set.......... 414
14.2.105: All Meals Increment after 52 Weeks of Treatment - Change from Baseline - 9-point Self Measured Plasma Glucose Profile (mmol/L) - Empirical Distribution Plot - Full Analysis Set.. 415
14.2.106: Nocturnal Increment by Treatment Week - 9-point Self Measured Plasma Glucose Profile (mmol/L) – Descriptive Statistics - Full Analysis Set 416
14.2.107: Nocturnal Increment by Treatment Week – Change from Baseline - 9-point Self Measured Plasma Glucose Profile (mmol/L) - Descriptive Statistics - Full Analysis Set 419
14.2.108: Nocturnal Increment after 52 Weeks of Treatment - 9-point Self Measured Plasma Glucose Profile (mmol/L) - Statistical Analysis - Full Analysis Set...................... 422
14.2.109: Nocturnal Increment (Bedtime to 04:00) by Treatment Week – 9-point Self Measured Plasma Glucose Profile (mmol/L) -Mean Plot - Full Analysis Set 423
14.2.110: Nocturnal Increment (Bedtime to 04:00) by Treatment Week - Change from Baseline – 9-point Self Measured Plasma Glucose Profile (mmol/L) - Mean Plot - Full Analysis Set 424

14.2.111: Nocturnal Increment (Bedtime to 04:00) after 52 Weeks of Treatment - 9-point Self Measured Plasma Glucose Profile (mmol/L) - Empirical Distribution Plot - Full Analysis Set 425
14.2.112: Nocturnal Increment (Bedtime to 04:00) after 52 Weeks of Treatment– Change from Baseline - 9-point Self Measured Plasma Glucose Profile (mmol/L) - Empirical Distribution Plot - Full Analysis Set 426
14.2.113: Nocturnal Increment (Bedtime to Breakfast) by Treatment Week – 9-point Self Measured Plasma Glucose Profile (mmol/L) - Mean Plot - Full Analysis Set 427
14.2.114: Nocturnal Increment (Bedtime to Breakfast) by Treatment Week - Change from Baseline - 9-point Self Measured Plasma Glucose Profile (mmol/L) - Mean Plot - Full Analysis Set 428
14.2.115: Nocturnal Increment (Bedtime to Breakfast) after 52 Weeks of Treatment -9-point Self Measured Plasma Glucose Profile (mmol/L) - Empirical Distribution Plot - Full Analysis Set 429
14.2.116: Nocturnal Increment (Bedtime to Breakfast) after 52 Weeks of Treatment – Change from Baseline -9-point Self Measured Plasma Glucose Profile (mmol/L) - Empirical Distribution Plot - Full Analysis Set 430
14.2.117: Nocturnal Increment (04:00 to Breakfast) by Treatment Week – 9-point Self Measured Plasma Glucose Profile (mmol/L) – Mean Plot - Full Analysis Set 431
14.2.118: Nocturnal Increment (04:00 to Breakfast) by Treatment Week - Change from Baseline – 9-point Self Measured Plasma Glucose Profile (mmol/L) – Mean Plot - Full Analysis Set 432
14.2.119: Nocturnal Increment (04:00 to Breakfast) after 52 Weeks of Treatment -9-point Self Measured Plasma Glucose Profile (mmol/L) - Empirical Distribution Plot - Full Analysis Set 433
14.2.120: Nocturnal Increment (04:00 to Breakfast) after 52 Weeks of Treatment – Change from Baseline -9-point Self Measured Plasma Glucose Profile (mmol/L) - Empirical Distribution Plot - Full Analysis Set 434
14.2.121: Self Measured Plasma Glucose for Dose Adjustment (mmol/L) – Descriptive Statistics - Full Analysis Set 435
14.2.122: Self Measured Plasma Glucose for Dose Adjustment after 52 Treatment Weeks - Statistical Analysis - Full Analysis Set 441
14.2.123: Mean Before Breakfast Self Measured Plasma Glucose for Dose Adjustment (mmol/L) by Treatment Week - Mean Plot - Full Analysis Set 442
14.2.124: Mean Before Breakfast Self Measured Plasma Glucose for Dose Adjustment (mmol/L) by Treatment Week - IDegAsp OD - Box Plot - Full Analysis Set 443
14.2.125: Mean Before Breakfast Self Measured Plasma Glucose for Dose Adjustment (mmol/L) by Treatment Week - IGlar OD - Box Plot - Full Analysis Set 444
14.2.126: Within-Subject Variation in Self Measured Plasma Glucose for Dose Adjustment after 52 Treatment Weeks - Statistical Analysis - Full Analysis Set 445
14.2.127: Before Breakfast Titration Target (SMPG<5 mmol/L) by Treatment Week – Summary - Full Analysis Set 446
14.2.128: Time to Before Breakfast Titration Target is Met for the First Time (Treatment Week) - Kaplan-Meier Plot - Full Analysis Set 451
14.2.129: Time to Titration Target is Met for the First Time (Treatment Week) – Descriptive Statistics - Full Analysis Set 452
14.2.130: Time to Titration Target is Met for the First Time (Treatment Week) - Statistical Analysis - Full Analysis Set 453
14.3 Safety Data 454

14.3.1 Displays of Adverse Events....................454
14.3.1.1: Adverse Events - Treatment Emergent - Summary - Safety Analysis Set 455
14.3.1.2: Serious Adverse Events – Treatment Emergent - Summary - Safety Analysis Set....................456
14.3.1.3: Serious Adverse Events – Treatment Emergent - Summary - Extension Trial Set....................457
14.3.1.4: Adverse Events by System Organ Class and Preferred Term - Most Frequent [>=5%]- Treatment Emergent - Summary - Safety Analysis Set....................458
14.3.1.5: Adverse Events by System Organ Class and Preferred Term - Most Frequent [>=1%]- Treatment Emergent - Summary - Safety Analysis Set....................459
14.3.1.6: Serious Adverse Events by System Organ Class and Preferred Term - Most Frequent [>=5%]- Treatment Emergent - Summary - Safety Analysis Set....................462
14.3.1.7: Serious Adverse Events by System Organ Class and Preferred Term - Most Frequent [>=1%]- Treatment Emergent - Summary - Safety Analysis Set....................462
14.3.1.8: Adverse Events by System Organ Class and Preferred Term - Treatment Emergent - Summary - Safety Analysis Set....................463
14.3.1.9: Serious Adverse Events by System Organ Class and Preferred Term - Treatment Emergent - Summary - Safety Analysis Set 478
14.3.1.10: Serious Adverse Events by System Organ Class and Preferred Term - Treatment Emergent - Summary - Extension Trial Set....................482
14.3.1.11: Adverse Events Possibly or Probably Related to Investigational Medicinal Product by System Organ Class and Preferred Term - Treatment Emergent - Safety Analysis Set....................485
14.3.1.12: Adverse Events Related to Device by System Organ Class and Preferred Term - Treatment Emergent - Summary - Safety Analysis Set....................488
14.3.1.13: Serious Adverse Events Possibly or Probably Related to Investigational Medicinal Product by System Organ Class and Preferred Term - Treatment Emergent - Summary - Safety Analysis Set....................489
14.3.1.14: Serious Adverse Events Related to Device by System Organ Class and Preferred Term - Treatment Emergent - Summary - Safety Analysis Set....................490
14.3.1.15: Severe Adverse Events by System Organ Class and Preferred Term - Treatment Emergent - Summary - Safety Analysis Set 491
14.3.1.16: Severe Adverse Events Possibly or Probably Related to Investigational Medicinal Product by System Organ Class and Preferred Term - Treatment Emergent - Summary - Safety Analysis Set....................494
14.3.1.17: Severe Adverse Events Related to Device by System Organ Class and Preferred Term - Treatment Emergent - Summary - Safety Analysis Set....................495
14.3.1.18: Serious Severe Adverse Events by System Organ Class and Preferred Term - Treatment Emergent - Summary - Safety Analysis Set 496
14.3.1.19: Moderate Adverse Events by System Organ Class and Preferred Term - Treatment Emergent - Summary - Safety Analysis Set 499
14.3.1.20: Mild Adverse Events by System Organ Class and Preferred Term - Treatment Emergent - Summary - Safety Analysis Set 507
14.3.1.21: Adverse Events - Treatment Emergent - Mean Cumulative Function - Safety Analysis Set....................519
14.3.1.22: Serious Adverse Events - Treatment Emergent - Mean Cumulative Function - Safety Analysis Set 520
14.3.1.23: Adverse Events Leading To Dose Reduction - Treatment Emergent - Summary - Safety Analysis Set....................521

14.3.1.24: Neoplasms by System Organ Class and Preferred Term - Treatment Emergent - Summary - Safety Analysis Set 522
14.3.1.25: Allergic Reaction (Immunogenecity) by System Organ Class and Preferred Term - Treatment Emergent - Summary - Safety Analysis Set..... 523
14.3.1.26: Adverse Events Leading to Dose Reduction by System Organ Class and Preferred Term - Treatment Emergent - Summary - Safety Analysis Set..... 524
14.3.1.27: Adjudicated Cardiovascular Events - Summary - Safety Analysis Set 525
14.3.1.28: Injection Site Reaction by System Organ Class and Preferred Term - Treatment Emergent - Summary - Safety Analysis Set 526
14.3.1.29: Injection Site Reactions - Mean Cumulative Function - Safety Analysis Set 527
14.3.1.30: Medication Errors - Treatment Emergent - Summary - Safety Analysis Set..... 528
14.3.1.31: Hypoglycaemic Episodes by Classification - Treatment Emergent - Summary - Safety Analysis Set..... 530
14.3.1.32: Hypoglycaemic Episodes by Classification - Treatment Emergent - Summary - Extension Trial Set 531
14.3.1.33: Nocturnal Hypoglycaemic Episodes by Classification - Treatment Emergent - Summary - Safety Analysis Set 532
14.3.1.34: Nocturnal Hypoglycaemic Episodes by Classification - Treatment Emergent - Summary - Extension Trial Set..... 533
14.3.1.35: Hypoglycaemic Episodes by Classification and Time - Treatment Emergent - Summary - Safety Analysis Set..... 534
14.3.1.36: Hypoglycaemic Episodes by Classification and Time - Treatment Emergent - Summary - Extension Trial Set 538
14.3.1.37: Nocturnal Hypoglycaemic Episodes by Classification and Time - Treatment Emergent - Summary - Safety Analysis Set..... 546
14.3.1.38: Hypoglycaemic Episodes - Treatment Emergent - Statistical Analysis - Full Analysis Set..... 550
14.3.1.39: Hypoglycaemic Episodes - Treatment Emergent - Statistical Analysis - Extension Trial Set..... 551
14.3.1.40: Nocturnal Confirmed Hypoglycaemic Episodes – Treatment Emergent – Statistical Analysis - Full Analysis Set 552
14.3.1.41: Nocturnal Confirmed Hypoglycaemic Episodes – Treatment Emergent – Statistical Analysis - Extension Trial Set..... 553
14.3.1.42: Confirmed Hypoglycaemic Episodes – Treatment Emergent - Empirical Distribution Plot - Safety Analysis Set 554
14.3.1.43: Nocturnal Confirmed Hypoglycaemic Episodes – Treatment Emergent - Empirical Distribution Plot - Safety Analysis Set..... 555
14.3.1.44: Confirmed Hypoglycaemic Episodes – Treatment Emergent - Mean Cumulative Function - Safety Analysis Set 556
14.3.1.45: Confirmed Hypoglycaemic Episodes – Treatment Emergent - Mean Cumulative Function - Extension Trial Set..... 557
14.3.1.46: Nocturnal Confirmed Hypoglycaemic Episodes – Treatment Emergent - Mean Cumulative Function - Safety Analysis Set 558
14.3.1.47: Nocturnal Confirmed Hypoglycaemic Episodes – Treatment Emergent - Mean Cumulative Function - Extension Trial Set..... 559
14.3.1.48: Confirmed Hypoglycaemic Episodes – Treatment Emergent - Cumulative Frequency over Day - Safety Analysis Set..... 560
14.3.2 Listings of Deaths, Other Serious and Significant Adverse Events 561

14.3.2.1: Treatment Emergent Serious Adverse Events - Safety Analysis Set 561
14.3.2.2: Treatment Emergent Adverse Events with Fatal Outcome - Safety Analysis Set 574
14.3.2.3: Treatment Emergent Adverse Events Leading to Dose Reduction of IDegAsp/IGlar Insulin Dose - Safety Analysis Set 576
14.3.2.4: Adverse Events Leading to Withdrawal - Full Analysis Set 578
14.3.3 Narratives of Deaths, Other Serious and Certain Other Significant Adverse Events 583
14.3.3 Narratives of Deaths, Other Serious and Certain Other Significant Adverse Events 583
Abbreviation Statement 584
14.3.4 Abnormal Laboratory Value Listing (each patient) 667
14.3.4.1: Biochemistry Laboratory Test Results Outside Reference Range - Safety Analysis Set 667
14.3.4.2: Haematology Laboratory Test Results Outside Reference Range - Safety Analysis Set 700
14.3.4.3: Lipids Laboratory Test Results Outside Reference Range - Safety Analysis Set 736
14.3.4.4: Urinalysis With Positive Test Results - Safety Analysis Set 798
14.3.4.5: Cardiovascular Risk Markers Test Results Outside Reference Range - Safety Analysis Set 811
14.3.5 Laboratory Displays 853
14.3.5.1: Biochemistry by Treatment Week - Descriptive Statistics - Safety Analysis Set 854
14.3.5.2: Biochemistry by Treatment Week - Descriptive Statistics - Extension Trial Set 863
14.3.5.3: Biochemistry by Treatment Week - Change from Baseline - Descriptive Statistics - Safety Analysis Set 865
14.3.5.4: Biochemistry by Treatment Week - Change from Baseline - Descriptive Statistics - Extension Trial Set 874
14.3.5.5: Biochemistry by Treatment Week - Summary - Safety Analysis Set 876
14.3.5.6: Biochemistry by Treatment Week - Summary - Extension Trial Set 881
14.3.5.7: Biochemistry - Shift Table - Baseline to Week 52 - Safety Analysis Set 882
14.3.5.8: Biochemistry - Shift Table - Baseline to Week 52 - Extension Trial Set 885
14.3.5.9: Biochemistry - Alanine amino. Serum - Box Plot - Safety Analysis Set 886
14.3.5.10: Biochemistry - Alanine amino. Serum after 52 Weeks of Treatment - Empirical Distribution Plot - Safety Analysis Set 887
14.3.5.11: Biochemistry - Albumin Serum - Box Plot - Safety Analysis Set 888
14.3.5.12: Biochemistry - Albumin Serum after 52 Weeks of Treatment - Empirical Distribution Plot - Safety Analysis Set 889
14.3.5.13: Biochemistry - Total Bilirubin - Box Plot - Safety Analysis Set 890
14.3.5.14: Biochemistry - Total Bilirubin after 52 Weeks of Treatment – Empirical Distribution Plot - Safety Analysis Set 891
14.3.5.15: Biochemistry - AST - Box Plot - Safety Analysis Set 892
14.3.5.16: Biochemistry - AST after 52 Weeks of Treatment - Empirical Distribution Plot - Safety Analysis Set 893
14.3.5.17: Biochemistry - Creatinine - Box Plot - Safety Analysis Set 894

14.3.5.18: Biochemistry - Creatinine after 52 Weeks of Treatment - Empirical Distribution Plot - Safety Analysis Set 895
14.3.5.19: Biochemistry - Alkaline phosphatase - Box Plot - Safety Analysis Set 896
14.3.5.20: Biochemistry - Alkaline phosphatase after 52 Weeks of Treatment - Empirical Distribution Plot - Safety Analysis Set........ 897
14.3.5.21: Biochemistry - Total Protein - Box Plot - Safety Analysis Set........ 898
14.3.5.22: Biochemistry - Total Protein after 52 Weeks of Treatment - Empirical Distribution Plot - Safety Analysis Set 899
14.3.5.23: Biochemistry - Sodium - Box Plot - Safety Analysis Set 900
14.3.5.24: Biochemistry - Sodium after 52 Weeks of Treatment - Empirical Distribution Plot - Safety Analysis Set 901
14.3.5.25: Biochemistry - Potassium - Box Plot - Safety Analysis Set 902
14.3.5.26: Biochemistry - Potassium after 52 Weeks of Treatment - Empirical Distribution Plot - Safety Analysis Set 903
14.3.5.27: Haematology by Treatment Week - Descriptive Statistics - Safety Analysis Set........ 904
14.3.5.28: Haematology by Treatment Week - Change from Baseline - Descriptive Statistics - Safety Analysis Set........ 914
14.3.5.29: Haematology by Treatment Week - Summary - Safety Analysis Set........ 924
14.3.5.30: Haematology - Shift Table - Baseline to Week 52 - Safety Analysis Set........ 929
14.3.5.31: Haematology - Neutrophils - Box Plot - Safety Analysis Set........ 932
14.3.5.32: Haematology - Neutrophils after 52 Weeks of Treatment - Empirical Distribution Plot - Safety Analysis Set 933
14.3.5.33: Haematology - Lymphocytes - Box Plot - Safety Analysis Set........ 934
14.3.5.34: Haematology - Lymphocytes after 52 Weeks of Treatment - Empirical Distribution Plot - Safety Analysis Set 935
14.3.5.35: Haematology - Monocytes - Box Plot - Safety Analysis Set........ 936
14.3.5.36: Haematology - Monocytes after 52 Weeks of Treatment - Empirical Distribution Plot - Safety Analysis Set 937
14.3.5.37: Haematology - Eosinophils - Box Plot - Safety Analysis Set........ 938
14.3.5.38: Haematology - Eosinophils after 52 Weeks of Treatment - Empirical Distribution Plot - Safety Analysis Set 939
14.3.5.39: Haematology - Basophils - Box Plot - Safety Analysis Set........ 940
14.3.5.40: Haematology - Basophils after 52 Weeks of Treatment - Empirical Distribution Plot - Safety Analysis Set 941
14.3.5.41: Haematology - Erythrocytes - Box Plot - Safety Analysis Set 942
14.3.5.42: Haematology - Erythrocytes after 52 Weeks of Treatment - Empirical Distribution Plot - Safety Analysis Set 943
14.3.5.43: Haematology - Haematocrit - Box Plot - Safety Analysis Set........ 944
14.3.5.44: Haematology - Haematocrit after 52 Weeks of Treatment - Empirical Distribution Plot - Safety Analysis Set 945
14.3.5.45: Haematology - Haemoglobin - Box Plot - Safety Analysis Set........ 946
14.3.5.46: Haematology - Haemoglobin after 52 Weeks of Treatment - Empirical Distribution Plot - Safety Analysis Set 947
14.3.5.47: Haematology - Leukocytes - Box Plot - Safety Analysis Set 948
14.3.5.48: Haematology - Leukocytes after 52 Weeks of Treatment - Empirical Distribution Plot - Safety Analysis Set 949

14.3.5.49: Haematology - Thrombocytes - Box Plot - Safety Analysis Set 950
14.3.5.50: Haematology - Thrombocytes after 52 Weeks of Treatment - Empirical Distribution Plot - Safety Analysis Set 951
14.3.5.51: Lipids by Treatment Week - Descriptive Statistics - Safety Analysis Set.......... 952
14.3.5.52: Lipids by Treatment Week - Change from Baseline - Descriptive Statistics - Safety Analysis Set 956
14.3.5.53: Lipids after 52 Weeks of Treatment - Statistical Analysis - Extension Trial Set........................ 960
14.3.5.54: Lipids after 52 Weeks of Treatment - Statistical Analysis - Full Analysis Set ... 962
14.3.5.55: Lipids by Treatment Week - Summary - Safety Analysis Set........................ 964
14.3.5.56: Lipids - Shift Table - Baseline to Week 52 - Safety Analysis Set........................ 966
14.3.5.57: Lipid - HDL Cholesterol - Box Plot - Safety Analysis Set........................ 967
14.3.5.58: Lipid - HDL Cholesterol after 52 Weeks of Treatment - Empirical Distribution Plot - Safety Analysis Set 968
14.3.5.59: Lipid - LDL Cholesterol - Box Plot - Safety Analysis Set 969
14.3.5.60: Lipid - LDL Cholesterol after 52 Weeks of Treatment - Empirical Distribution Plot - Safety Analysis Set 970
14.3.5.61: Lipid - Total Cholesterol Serum - Box Plot - Safety Analysis Set 971
14.3.5.62: Lipid - Total Cholesterol Serum after 52 Weeks of Treatment - Empirical Distribution Plot - Safety Analysis Set 972
14.3.5.63: Lipid - Triglycerides Serum - Box Plot - Safety Analysis Set........................ 973
14.3.5.64: Lipid - Triglycerides Serum after 52 Weeks of Treatment - Empirical Distribution Plot - Safety Analysis Set 974
14.3.5.65: Urinary Albumin-to-Creatinine Ratio by Treatment Week - Descriptive Statistics - Safety Analysis Set........................ 975
14.3.5.66: Urinary Albumin-to-Creatinine Ratio by Treatment Week - Change from Baseline - Descriptive Statistics - Safety Analysis Set 976
14.3.5.67: Urinary Albumin-to-Creatinine Ratio by Treatment Week - Summary - Safety AnalysisSet 977
14.3.5.68: Urinary Albumin-to-Creatinine Ratio - Shift Table - Baseline to Week 52 - Safety Analysis Set 978
14.3.5.69: Urinary Albumin-to-Creatinine Ratio - Box Plot - Safety Analysis Set............. 979
14.3.5.70: Urinary Albumin-to-Creatinine Ratio after 52 Weeks of Treatment – Empirical Distribution Plot - Safety Analysis Set........................ 980
14.3.5.71: Urine by Sticks - Summary - Safety Analysis Set........................ 981
14.3.5.72: Urine by Sticks - Shift Table - Baseline to Week 52 - Safety Analysis Set 983
14.3.5.73: Cardiovascular Risk Markers by Treatment Week - Descriptive Statistics - Safety Analysis Set 984
14.3.5.74: Cardiovascular Risk Markers by Treatment Week - Change from Baseline - Descriptive Statistics - Safety Analysis Set 986
14.3.5.75: Cardiovascular Risk Markers by Treatment Week - Summary - Safety Analysis Set........................ 988
14.3.5.76: Cardiovascular Risk Markers after 52 Weeks of Treatment – Statistical Analysis - Extension Trial Set........................ 989
14.3.5.77: Cardiovascular Risk Markers after 52 Weeks of Treatment – Statistical Analysis - Full Analysis Set........................ 990
14.3.5.78: Cardiovascular Risk Markers - Shift Table - Baseline to Week 52 - Safety Analysis Set........................ 991

14.3.5.79: Cardiovascular Risk Marker - C-reactive protein (mg/L) - Box Plot - Safety Analysis Set....... 992
14.3.5.80: Cardiovascular Risk Marker - C-reactive protein (mg/L) after 52 Weeks of Treatment - Empirical Distribution Plot - Safety Analysis Set....... 993
14.3.5.81: Cardiovascular Risk Marker -BNP (pmol/L) - Box Plot - Safety Analysis Set.... 994
14.3.5.82: Cardiovascular Risk Marker - BNP (pmol/L) after 52 Weeks of Treatment - Empirical Distribution Plot - Safety Analysis Set....... 995
14.3.6 Other Safety Observations Displays....... 996
14.3.6.1: Physical Examination by Treatment Week - Summary - Safety Analysis Set....... 997
14.3.6.2: Physical Examination - Shift Table - Baseline to Week 52 - Safety Analysis Set....... 1004
14.3.6.3: Vital Signs by Treatment Week - Descriptive Statistics - Safety Analysis Set....... 1006
14.3.6.4: Vital Signs by Treatment Week - Change from Baseline - Descriptive Statistics - Safety Analysis Set....... 1009
14.3.6.5: Diastolic Blood Pressure - Mean Plot - Safety Analysis Set....... 1012
14.3.6.6: Diastolic Blood Pressure - Change from Baseline - Mean Plot - Safety Analysis Set....... 1013
14.3.6.7: Diastolic Blood Pressure after 52 Weeks of Treatment - Empirical Distribution Plot - Safety Analysis Set....... 1014
14.3.6.8: Systolic Blood Pressure - Mean Plot - Safety Analysis Set....... 1015
14.3.6.9: Systolic Blood Pressure - Change from Baseline - Mean Plot - Safety Analysis Set....... 1016
14.3.6.10: Systolic Blood Pressure after 52 Weeks of Treatment - Empirical Distribution Plot - Safety Analysis Set....... 1017
14.3.6.11: Pulse - Mean Plot - Safety Analysis Set....... 1018
14.3.6.12: Pulse - Change from Baseline - Mean Plot - Safety Analysis Set....... 1019
14.3.6.13: Pulse after 52 Weeks of Treatment - Empirical Distribution Plot - Safety Analysis Set....... 1020
14.3.6.14: ECG by Treatment Week - Summary - Safety Analysis Set....... 1021
14.3.6.15: ECG - Shift Table - Baseline to Week 52 - Safety Analysis Set....... 1022
14.3.6.16: Funduscopy/Fundusphotography by Treatment Week - Summary - Safety Analysis Set....... 1023
14.3.6.17: Funduscopy / Fundusphotography - Shift Table - Baseline to Week 52 - Safety Analysis Set....... 1025
14.3.6.18: Body Weight and BMI by Treatment Week - Descriptive Statistics - Safety Analysis Set....... 1026
14.3.6.19: Body Weight and BMI by Treatment Week - Change from Baseline - Descriptive Statistics - Safety Analysis Set....... 1030
14.3.6.20: Body Weight after 52 Weeks of Treatment - Statistical Analysis - Full Analysis Set....... 1032
14.3.6.21: Body Weight - Mean Plot - Safety Analysis Set....... 1033
14.3.6.22: Body Weight – Change from Baseline - Mean Plot - Safety Analysis Set....... 1034
14.3.6.23: Body Weight by Treatment week - Box Plot - Safety Analysis Set....... 1035
14.3.6.24: Body Weight after 52 Weeks of Treatment - Empirical Distribution Plot - Safety Analysis Set....... 1036
14.3.6.25: Body Weight after 52 Weeks of Treatment - Change from Baseline - Empirical Distribution Plot - Safety Analysis Set....... 1037

14.3.6.26: Insulin Degludec Specific Antibodies (% B/T) by Treatment Week - Descriptive Statistics - Safety Analysis Set 1038
14.3.6.27: Insulin Glargine Specific Antibodies (% B/T) by Treatment Week - Descriptive Statistics - Safety Analysis Set 1039
14.3.6.28: Insulin Aspart Specific Antibodies (% B/T) by Treatment Week - Descriptive Statistics - Safety Analysis Set 1040
14.3.6.29: Cross-reacting Antibodies to Human Insulin (% B/T) by Treatment Week - Descriptive Statistics - Safety Analysis Set 1041
14.3.6.30: Total Antibodies (% B/T) by Treatment Week - Descriptive Statistics - Safety Analysis Set 1042
14.3.6.31: IDegAsp / IGlar Specific Antibodies (% B/T) - Mean Plot - Safety Analysis Set........ 1043
14.3.6.32: IDegAsp / IGlar Specific Antibodies (% B/T) - Mean Plot - Extension Trial Set........ 1044
14.3.6.33: IDegAsp / IGlar Specific Antibodies (% B/T) - Mean Plot - Observed Values - Safety Analysis Set 1045
14.3.6.34: IDegAsp / IGlar Specific Antibodies (% B/T) - Mean Plot - Observed Values - Extension Trial Set........ 1046
14.3.6.35: Insulin Aspart Specific Antibodies (% B/T) - Mean Plot - Safety Analysis Set........ 1047
14.3.6.36: Insulin Aspart Specific Antibodies (% B/T) - Mean Plot - Extension Trial Set........ 1048
14.3.6.37: Insulin Aspart Specific Antibodies (% B/T) - Mean Plot - Observed Values - Safety Analysis Set 1049
14.3.6.38: Insulin Aspart Specific Antibodies (% B/T) - Mean Plot - Observed Values - Extension Trial Set........ 1050
14.3.6.39: Cross-reacting Antibodies to Human Insulin (% B/T) - Mean Plot - Safety Analysis Set........ 1051
14.3.6.40: Cross-reacting Antibodies to Human Insulin (% B/T) - Mean Plot - Extension Trial Set........ 1052
14.3.6.41: Cross-reacting Antibodies to Human Insulin (% B/T) - Mean Plot - Observed Values - Safety Analysis Set 1053
14.3.6.42: Cross-reacting Antibodies to Human Insulin (% B/T) - Mean Plot - Observed Values - Extension Trial Set........ 1054
14.3.6.43: Total Antibodies (% B/T) - Mean Plot - Safety Analysis Set........ 1055
14.3.6.44: Total Antibodies (% B/T) - Mean Plot - Extension Trial Set 1056
14.3.6.45: Total Antibodies (% B/T) - Mean Plot - Observed Values - Safety Analysis Set........ 1057
14.3.6.46: Total Antibodies (% B/T) - Mean Plot - Observed Values - Extension Trial Set........ 1058
14.3.6.47: Specific Antibodies (% B/T) against HbA1c (%) after 52 Weeks of Treatment - Scatter Plot - Safety Analysis Set........ 1059
14.3.6.48: Specific Antibodies (% B/T) against HbA1c (%) after 52 Weeks of Treatment - Scatter Plot – Extension Trial Set........ 1060
14.3.6.49: Specific Antibodies (% B/T) against Change from Baseline in HbA1c (%) after 52 Weeks of Treatment - Scatter Plot - Safety Analysis Set........ 1061
14.3.6.50: Specific Antibodies (% B/T) against Change from Baseline in HbA1c (%) after 52 Weeks of Treatment - Scatter Plot - Extension Trial Set........ 1062

14.3.6.51: Cross-reacting Antibodies to Human Insulin (% B/T) against HbA1c (%) after 52 Weeks of Treatment - Scatter Plot - Safety Analysis Set 1063
14.3.6.52: Cross-reacting Antibodies to Human Insulin (% B/T) against HbA1c (%) after 52 Weeks of Treatment - Scatter Plot - Extension Trial Set 1064
14.3.6.53: Cross-reacting Antibodies to Human Insulin (% B/T) against Change from Baseline in HbA1c (%) after 52 Weeks of Treatment - Scatter Plot - Safety Analysis Set 1065
14.3.6.54: Cross-reacting Antibodies to Human Insulin (% B/T) against Change from Baseline in HbA1c (%) after 52 Weeks of Treatment - Scatter Plot - Extension Trial Set 1066
14.3.6.55: Total Antibodies (% B/T) against HbA1c (%) after 52 Weeks of Treatment - Scatter Plot - Safety Analysis Set 1067
14.3.6.56: Total Antibodies (% B/T) against HbA1c (%) after 52 Weeks of Treatment - Scatter Plot - Extension Trial Set 1068
14.3.6.57: Total Antibodies (% B/T) against Change from Baseline in HbA1c (%) after 52 Weeks of Treatment - Scatter Plot - Safety Analysis Set 1069
14.3.6.58: Total Antibodies (% B/T) against Change from Baseline in HbA1c (%) after 52 Weeks of Treatment - Scatter Plot - Extension Trial Set 1070
14.3.6.59: Specific Antibodies (% B/T) against Total Daily Insulin Dose (U/kg) after 52 Weeks of Treatment - Scatter Plot - Safety Analysis Set 1071
14.3.6.60: Specific Antibodies (% B/T) against Total Daily Insulin Dose (U/kg) after 52 Weeks of Treatment - Scatter Plot - Extension Trial Set 1072
14.3.6.61: Cross-reacting Antibodies to Human Insulin (% B/T) against Total Daily Insulin Dose (U/kg) after 52 Weeks of Treatment - Scatter Plot - Safety Analysis Set 1073
14.3.6.62: Cross-reacting Antibodies to Human Insulin (% B/T) against Total Daily Insulin Dose (U/kg) after 52 Weeks of Treatment - Scatter Plot - Extension trial Set ... 1074
14.3.6.63: Total Antibodies (% B/T) against Total Daily Insulin Dose (U/kg) after 52 Weeks of Treatment - Scatter Plot - Safety Analysis Set 1075
14.3.6.64: Total Antibodies (% B/T) against Total Daily Insulin Dose (U/kg) after 52 Weeks of Treatment - Scatter Plot - Extension Trial Set 1076
14.3.7 Other Safety Observations Lists 1077
14.3.7.1: Vital Signs - Safety Analysis Set 1077
14.3.7.2: ECG Findings - Safety Analysis Set 1130
14.3.7.3: Funduscopy/Fundusphotography Findings - Safety Analysis Set 1192
14.3.7.4: Antibodies - Safety Analysis Set 1310
14.3.7.5: Physical Examination Findings - Safety Analysis Set 1734
14.3.7.6: Body Measurements - Safety Analysis Set 2021
15 Reference List 2155

List of In-Text Figures

Page

Figure 9–1 Trial Design 38
Figure 9–2 Classification of hypoglycaemia according to ADA 68
Figure 11–1 HbA1c (%) by Treatment Week – Mean Plot – Full Analysis Set 106
Figure 11–2 Fasting Plasma Glucose (mmol/L) by Treatment Week – Mean Plot – Full Analysis Set 113
Figure 11–3 9-point Self Measured Plasma Glucose Profile (mmol/L) at Baseline (Left) and after 52 Weeks (Right) - LOCF - Mean Plot - Full Analysis Set 115
Figure 12–1 Total Daily Insulin Dose (Actual) in Units by Treatment Week - Mean Plot - Safety Analysis Set 125
Figure 12–2 Confirmed Hypoglycaemic Episodes – Treatment Emergent - Mean Cumulative Function - Safety Analysis Set 150
Figure 12–3 Nocturnal Confirmed Hypoglycaemic Episodes – Treatment Emergent - Mean Cumulative Function - Safety Analysis Set 153
Figure 12–4 Cross-reacting Antibodies to Human Insulin (% B/T) - Mean Plot - Safety Analysis Set 155
Figure 12–5 Body Weight - Mean Plot - Safety Analysis Set 162

List of In-Text Tables

Page

Table 6–1 Contract Research Organisations ... 30
Table 9–1 Trial Product ... 48
Table 9–2 Titration Algorithm for Insulin Degludec/Insulin Aspart or Insulin Glargine Doses ... 50
Table 9–3 Reduction of Insulin Degludec/Insulin Aspart or Insulin Glargine Doses ... 50
Table 9–4 Trial Flow Chart- Main Trial ... 52
Table 9–5 Trial Flow Chart- Extension Trial ... 55
Table 9–6 9-point profile (SMPG) (√) with additional 1-point profiles (SMPG) (X) ... 61
Table 9–7 Substantial Protocol Amendments ... 84
Table 10–1 Subject Disposition - Summary ... 88
Table 10–2 Important Protocol Deviations- Main Trial ... 91
Table 10–3 Summary of Important Protocol Deviations- Extension Trial ... 95
Table 11–1 Analysis Sets ... 96
Table 11–2 Demographics and Baseline Characteristics –Summary – Full Analysis Set ... 98
Table 11–3 Demographics and Baseline Characteristics – Summary – Extension Trial Set ... 99
Table 11–4 Baseline and Diabetes Characteristics – Descriptive Statistics – Full Analysis Set ... 100
Table 11–5 Baseline and Diabetes Characteristics – Descriptive Statistics – Extension Trial Set ... 101
Table 11–6 Antidiabetic Treatment Regimen at Screening – Full Analysis Set ... 103
Table 11–7 Diabetes Complications at Screening – Summary – Full Analysis Set ... 104
Table 11–8 HbA1c (%) after 52 Weeks of Treatment - Statistical Analysis - Full Analysis Set ... 107
Table 11–9 HbA1c (%) after 52 Weeks of Treatment - Statistical Analysis - Extension Trial Set ... 108
Table 11–10 HbA1c (%) after 52 Weeks of Treatment - Statistical Sensitivity Analysis - PP Analysis Set ... 108
Table 11–11 Responder for HbA1c by Treatment Week – Summary - Full Analysis Set ... 109
Table 11–12 Responder for HbA1c at End of Trial - Statistical Analysis - Full Analysis Set ... 110
Table 11–13 Responder for HbA1c at End of Trial Without Hypoglycaemia – Summary - Full Analysis Set ... 111
Table 11–14 Responder for HbA1c at End of Trial Without Hypoglycaemia - Statistical Analysis - Full Analysis Set ... 112
Table 11–15 Fasting Plasma Glucose (mmol/L) after 52 Weeks of Treatment - Statistical Analysis - Full Analysis Set ... 114
Table 11–16 Self Measured Plasma Glucose for Dose Adjustment (mmol/L) – Descriptive Statistics - Full Analysis Set ... 118
Table 11–17 Self Measured Plasma Glucose for Dose Adjustment after 52 Treatment Weeks - Statistical Analysis - Full Analysis Set ... 118
Table 12–1 Exposure - Descriptive Statistics - Safety Analysis Set ... 122
Table 12–2 IDegAsp/ IGlar Dose (Actual) in Units by Treatment Week - Descriptive Statistics - Safety Analysis Set ... 124
Table 12–3 IDegAsp/ IGlar Dose Ratio after 52 Weeks of Treatment - Summary - Safety Analysis Set ... 126
Table 12–4 Adverse Events - Treatment Emergent - Summary - Safety Analysis Set ... 128
Table 12–5 Adverse Events by System Organ Class and Preferred Term - Most Frequent [>=5%]- Treatment Emergent - Summary - Safety Analysis Set ... 129
Table 12–6 Allergic Reactions (Immunogenicity) - Safety Analysis Set ... 131

Table 12–7 Neoplasms Events Safety Analysis Set 132
Table 12–8 Adverse Events Possibly or Probably Related to Investigational Medicinal Product by System Organ Class and Preferred Term - Treatment Emergent - Safety Analysis Set 133
Table 12–9 Serious Adverse Events – Treatment Emergent - Summary - Safety Analysis Set 137
Table 12–10 Adjudicated Cardiovascular Events - Summary - Safety Analysis Set 138
Table 12–11 Summary of Major Adverse Cardiovascular Events 139
Table 12–12 Serious Adverse Events Possibly or Probably Related Investigational Medicinal Product by System Organ Class and Preferred Term - Treatment Emergent - Summary - Safety Analysis Set 143
Table 12–13 Narratives for SAEs Possibly/ Probably Related to Trial Drug 144
Table 12–14 Subjects with Adverse Events leading to Withdrawal 146
Table 12–15 Hypoglycaemic Episodes by Classification – Treatment Emergent – Summary - Safety Analysis Set 149
Table 12–16 Hypoglycaemic Episodes - Treatment Emergent - Statistical Analysis - Full Analysis Set 151
Table 12–17 Nocturnal Hypoglycaemic Episodes by Classification - Treatment Emergent - Summary - Safety Analysis Set 152
Table 12–18 Nocturnal Confirmed Hypoglycaemic Episodes – Treatment Emergent – Statistical Analysis - Full Analysis Set 154
Table 12–19 Cross-reacting Antibodies to Human Insulin (% B/T) by Treatment Week - Descriptive Statistics - Safety Analysis Set 156

List of Appendices

16.1 Study Information

16.1.1 Protocol and Protocol Amendments
16.1.2 Sample Case Report Forms
16.1.3 List of Independent Ethics Committees or Institutional Review Boards
16.1.4 List and Description of Investigators and Other Important Participants in the Study
16.1.5 Signatures of Principal or Coordinating Investigator(s)
16.1.6 Listing of Patients Receiving Test Drug(s)/Investigational Product(s) from Specific Batches
16.1.7 Randomisation Scheme and Codes
16.1.8 Audit Certificates
16.1.9 Documentation of Statistical Methods
16.1.10 Documentation of Inter-laboratory Standardisation Methods and Quality Assurance Procedures
16.1.11 Publications based on the Study
16.1.12 Important Publications Referenced in the Report

16.2 Patient Data Listings

16.2.1 Discontinued Patients
16.2.2 Protocol Deviations
16.2.3 Patients Excluded from the Efficacy Analysis
16.2.4 Demographic Data
16.2.5 Compliance and/or Drug Concentration Data
16.2.6 Individual Efficacy Response Data
16.2.7 Adverse Event Listings (Each Patient)
16.2.8 Listing of Individual Laboratory Measurements by Patient

16.3 Case Report Forms

16.3.1 CRFs for Deaths, other Serious Adverse Events, and Withdrawals for AE
16.3.2 Other CRFs submitted

16.4 Individual Patient Data Listings (US Archival Listings)

4 List of Abbreviations and Definition of Terms

%B/T	percentage bound/total
ADA	American Diabetes Association
AEs	adverse events
ALAT	alanine aminotransferase
ANOVA	analysis of variance
AP	alkaline phosphatase
ASAT	aspartate aminotransferase
AST	aspartate aminotransferase
BID	twice daily
BMI	body mass index
BP	blood pressure
CGM	continuous glucose monitoring
CI	confidence interval
CLAE	clinical laboratory adverse event
COCPIT	Novo Nordisk complaint handling system
CPMP	Committee for Proprietary Medicinal Products
CRF	case report form
CRO	contract research organisation
CTR	clinical trial report
CV	coefficient of variation
CVD	cardiovascular disease
DBL	Data Base Lock
DBP	diastolic blood pressure
DiabMedSat	Diabetes Medication Satisfaction Questionnaire
DPM	Diabetes Productivity Measure Questionnaire
DPP-4	Dipeptidyl peptidase-4
DUN	dispensing unit number
EAC	Event Adjudication Committee
ECG	electrocardiogram
eCRFs	electronic case report forms
EOT	End-of-text
ELISA	enzyme-linked immunosorbent assay
ETS	extension trial set
EU CE	European Union Conformité Européenne
FAS	full analysis set
FDA	Food and Drug Administration
FPG	fasting plasma glucose
FU	follow-up
GCP	Good Clinical Practice
GLP-1	glucagon-like peptide-1
HbA_{1c}	glycosylated haemoglobin

HDL	high density lipoproteins
Hg	mercury
HOMA	Homeostasis Model Assessment
hsCRP	high sensitive C-reactive protein
IAsp	insulin aspart
ICH	International Conference on Harmonisation
ID	identification
IDF	International Diabetes Federation
IDeg	insulin degludec
IDegAsp	insulin degludec and insulin aspart
IDegAsp (F)	The IDegAsp drug product consists of two drug substances (70 volume% IDeg + 30 volume% IAsp) 100 U/mL
IEC	independent ethics committee
IG	interstitial glucose
IgE	immunoglobulin E
IGlar	insulin glargine
IRB	institutional review board (United States ethics committee)
ITT	intention to treat
IV/WRS	interactive voice/web response system
Kg	kilogram
LDL	low density lipoproteins
lb	pounds
LDL	low density lipoproteins
LOCF	last observation carried forward
LSMeans	least square means
MACE	major adverse cardiovascular event
MAR	missing at random
MAO	monoamine oxidase
MedDRA	Medical Dictionary for Regulatory Activities
MESI	medical events of special interest
mL	millilitre
mm	millimetre
NT-Pro BNP	brain natriuretic peptide
NPH	Neutral Protamine Hagedorn
NYHA	New York Heart Association
OAD	oral antidiabetic drug
OD	once daily
PD	pharmacodynamic
PDS290	pre-filled insulin injection disposable pen device
PG	plasma glucose
PK	pharmacokinetic
PP	per protocol
PRO	patient reported outcome

PYE	patient year exposure
PT	Preferred Term
SAE	serious adverse event
SBP	systolic blood pressure
s.c.	subcutaneous
SD	standard deviation
SE	standard error of the mean
SF-36 v2	short-form 36 health survey version 2
SGOT	serum glutamic oxaloacetic transaminase
SGPT	serum glutamic pyruvic transaminase
SMPG	self-measured plasma glucose
SMQ	standard MedDRA query
SOC	System Organ Class
SPC	summary of product characteristics
SU	Sulphonylurea
TEAE	treatment emergent adverse event
T-T-T	treat-to-target
TRIM-D	Treatment Related Impact Measure – Diabetes
TZDs	thiazolidinediones
UK	United Kingdom
US	United States

Terms

I454: Insulin 454, Synonym previously used for insulin degludec drug substance

SIAC: Soluble Insulin Analogue Combination, NN5401. Synonym previously used for insulin degludec and insulin aspart.

5 Ethics

5.1 Independent Ethics Committee (IEC) or Institutional Review Board (IRB)

The protocol, the protocol amendments (see Appendix 16.1.1), the consent form, and the subject information sheet were reviewed and/or approved by appropriate authorities according to local regulations, and by the local IECs, an appropriately constituted review board (see Appendix 16.1.3), prior to trial initiation.

5.2 Ethical Conduct of the Trial

The trial was performed in accordance with the "Declaration of Helsinki"[1] and its amendments in force at the initiation of the trial. The subjects were informed of the risks and benefits of the trial. The trial also followed International Conference on Harmonisation (ICH) Good Clinical Practice (GCP).[2]

5.3 Subject Information and Consent

The subjects were informed verbally and in writing that they could withdraw from the trial at any time for any reason. Consent was obtained in writing prior to any trial-related activities and a copy of the informed consent form was given to the subject. The Investigators retained the original informed consent forms. The consent forms were available to Novo Nordisk for inspection. (The master subject information sheet and consent form are in Appendix 16.1.3).

Separated protocols, subject information and informed consent forms were prepared for both main trial and extension trial. Verbal and written information was provided to the subjects and the informed consent form was signed by the subject and the Investigator.

The subjects were covered by the sponsor's insurance according to local legal requirements.

6 Investigators and Trial Administrative Structure

Trial Sites

A total of 93 sites in 8 countries screened subjects in the main trial: Austria (7 sites), India (7 sites), Republic of Korea (5 sites), Poland (6 sites), Russia (10 sites), Spain (9 sites), Turkey (6 sites), and United States (43 sites).

Of these sites, 76 sites in 8 countries enrolled subjects during the extension trial: Austria (4 sites), India (7 sites), Republic of Korea (5 sites), Poland (6 sites), Russia (8 sites), Spain (9 sites), Turkey (5 sites), and United States (32 sites).

One principal investigator was appointed for each trial site. A list of the principal investigators, sub-investigators, and other important persons who were involved in the conduct of the trial, and their curricula vitae are provided in Appendix 16.1.4.

The following investigator was designated signatory investigator for the trial (see Appendix 16.1.5):
Dr. Ajay Kumar
Diabetes Care & Research Centre
GC1B, Near Over Bridge, Kankerbagh
Patna 800020
India

Sponsor

Novo Nordisk A/S, Denmark was responsible for the preparation of the protocol, electronic case report forms (eCRFs), supply of trial products and stated equipment, monitoring, data management, statistics, and the clinical trial report (CTR).

Novo Nordisk A/S has constituted an internal safety committee during the development program of insulin degludec (IDeg) and insulin degludec/insulin aspart (IDegAsp) to perform ongoing safety surveillance. The safety committee was blinded and could recommend unblinding of any data for further analysis. In case this possibility occurred, an independent ad hoc group was to be established to maintain the blinding.

An independent external Event Adjudication Committee (EAC) (see Appendix 16.1.4) was constituted for the trial to perform ongoing adjudication, standardisation and assessment of cardiovascular events in accordance with pre-defined classifications. The following events were to be evaluated and adjudicated by the EAC in an independent and blinded manner:

- Acute coronary syndrome (including myocardial infarction)
- Stroke
- Cardiovascular death

The titration of insulin doses was monitored by Quintiles and reviewed by an internal titration committee composed of members from Novo Nordisk, and any significant changes from the titration algorithm were addressed.

Contract Research Organisation

The following contract research organisations (CRO) were used; see Table 6–1.

Table 6–1 Contract Research Organisations

Name	Address	Responsibility
Quintiles Laboratories Europe<br>(Central laboratory)	Quintiles Laboratories Europe<br>The Alba Campus<br>Rose Bank<br>Livingston<br>West Lothian<br>EH54 7EG<br>Scotland, UK	All laboratory analyses for:<br>Austria, Poland, Russia, Spain, Turkey (with exception of insulin antibody analyses)
Quintiles Laboratories<br>(Central laboratory)	Quintiles Laboratories<br>Laboratory Division,<br>301-A-2, The Leela Business Park<br>Andheri-Kurla Road<br>Andheri (E)<br>Mumbai 400 059<br>India	All laboratory analyses for India (with exception of insulin antibody analyses)
Quintiles East Asia Pte. Ltd.<br>(Central laboratory)	Quintiles East Asia Pte. Ltd.<br>79 Science Park Drive<br>#04-08<br>Cintech IV<br>Singapore Science Park 1<br>Singapore 118264	All laboratory analyses for Republic of Korea (with exception of insulin antibody analyses)
Quintiles Laboratories Ltd.<br>(Central laboratory)	Quintiles Laboratories Limited<br>1600 Terrell Mill Road<br>Suit 100<br>Marietta<br>GA 30067<br>United States	All laboratory analyses for US (with exception of insulin antibody analyses)
**Titration Surveillance**		
Quintiles East Asia Pte. Ltd.	Quintiles East Asia Pte Ltd<br>79 Science Park Drive, #06-08<br>Cintech IV<br>Singapore Science Park I<br>Singapore 118264	Titration surveillance
Quintiles	Quintiles Research Triangle Park (QRTP, USA)<br>4820 Emperor Blvd.<br>Durham NC 27703<br>United States	Titration surveillance

Name	Address	Responsibility
Quintiles AG	Quintiles AG (QCHS, Switzerland)<br>Hochstrasse 50<br>CH-4053 Basel<br>Switzerland	Titration surveillance
**Insulin antibodies**		
Celerion Switzerland AG	Celerion Switzerland AG<br>Allmendstrasse 32<br>CH - Fehraltorf 8320<br>Switzerland	All laboratory insulin antibody analyses for : Austria, India, Poland, Russia, Republic of Korea, Spain, Turkey, US
**EDC**		
Phase Forward Incorporated	Phase Forward Incorporated<br>77 Fourth Avenue<br>Waltham, MA 02451<br>United States	eCRF
**IV/WRS**		
ClinPhone Plc[a]	ClinPhone Plc<br>Lady Bay House, Meadow Grove<br>Nottingham, NG2 3HF<br>United Kingdom	IV/WRS
Perceptive Informatics[a]	Perceptive Informatics<br>Lady Bay House, Meadow Grove<br>Nottingham, NG2 3HF<br>United Kingdom	IV/WRS
**Data management and CGM data handling**		
Quintiles Technologies	Quintiles Technologies (India)<br>Private Limited<br>3rd Floor, Brigade South Parade<br>10, M.G. Road<br>Bangalore 560 001<br>India	Data management and CGM data handling
**Management of Cardiovascular Event Adjudication**		
ICON Medical Imaging	ICON Medical Imaging<br>2800 Kelly Road<br>Suite 200<br>Warrington, PA 18976<br>USA	Management of cardiovascular event adjudication responsibility.

eCRF: electronic case report form; IV/WRS: interactive voice/web response system; CGM: continuous glucose monitoring

a: Same vendor. The company changed when the study was ongoing

All laboratories used standardised analysis methods and reference ranges, which were harmonised between laboratories; see Appendix 16.1.10. Laboratories were responsible for sending the data electronically to Novo Nordisk A/S.

7 Introduction

7.1 Therapeutic Area

Diabetes mellitus is characterised by chronic hyperglycaemia and encompasses various metabolic disorders. It is generally classified according to aetiological factors, where type 1 and type 2 diabetes mellitus constitute the vast majority of cases.

Type 2 diabetes mellitus is a progressive disorder characterised by a combination of insulin resistance at peripheral tissues and relative insulin secretion deficiency.

The current treatment cascade follows a stepwise approach comprising lifestyle changes in combination with pharmacological intervention. Metformin is recommended as initial pharmacological therapy, followed by combination therapy with other oral antidiabetic drugs (OADs), insulin or incretin based therapies (GLP-1 receptor agonists and DPP-4 inhibitors) as the disease progresses and in those with poorly controlled and long duration of diabetes.[3]

A number of landmark trials have demonstrated the importance of maintaining tight glycaemic control to reduce the risk of long-term complications associated with diabetes.[4-8] In general, it is recommended to aim for a glycosylated haemoglobin (HbA_{1c}) < 7.0%.[9] However, factors such as life expectancy, risk of hypoglycaemia and the presence of cardiovascular disease need to be considered for every patient before intensifying the therapeutic regimen.[9]

Insulin analogues have been developed that closely mimic endogenous insulin secretion as compared with human insulin preparations and are now an established part of diabetes management. Currently, insulin detemir (IDet) and insulin glargine (IGlar) cannot be mixed with any rapid-acting insulin analogues due to formulation issues.[10-13] This has prompted the development of IDeg, an ultra-long-acting basal insulin, as well as in a co-formulation with insulin aspart (IAsp).

7.2 Insulin Degludec/Insulin Aspart

Insulin degludec/insulin aspart (IDegAsp) is a combination of ultra-long-acting IDeg with rapid-acting IAsp, formulated to maintain the ability of IDeg to form multi-hexamers at the injection site without interfering with the rapid release of IAsp monomers into the circulation. IDegAsp is formulated such that IDeg exists as di-hexamers and IAsp as hexamers. After subcutaneous injection, IDeg di-hexamers assemble to form soluble and stable multi-hexamers, creating a depot of insulin in the subcutaneous tissue, while IAsp hexamers promptly separate into monomers that rapidly enter the circulation. IDeg monomers gradually separate from the multi-hexamers, resulting in a slow and continuous delivery of IDeg into the circulation.

IDegAsp provides main meal coverage as well as at least 24-hour basal coverage for people with diabetes mellitus when used once daily, and will therefore potentially offer distinct advantages (i.e., greater ease of use and compliance) over existing insulin therapies. As IDegAsp is soluble, the product can be injected immediately, without any mixing.

IDeg differs from human insulin in that the threonine in position B30 has been omitted, and a side chain consisting of a glutamic acid spacer with a fatty acid has been attached to position B29.

IAsp is homologous to human insulin, except that proline is substituted with aspartic acid at position B28. The rapid action of IAsp is related to a weakened tendency of the insulin molecules to self associate because of this modification, leading to faster absorption as compared with human insulin.

IDegAsp is designed to result in similar or improved glycaemic control compared to treatment with currently available insulin products. The basal component of IDegAsp, insulin degludec, is even longer acting than currently available basal insulin analogues. This is believed to result in an even more stable and predictable insulin profile with lower day to day variation in fasting plasma glucose (FPG) than those attained with the currently available insulin analogues. A significantly lower (four times) within-subject variability in glucose-lowering activity compared with IGlar has recently been demonstrated.[14]

7.3 Insulin Glargine

IGlar (Lantus®) is a long-acting insulin analogue, indicated for treatment of diabetes mellitus in combination with OADs and as part of a basal-bolus insulin regimen.

An amino-acid substitution at position A21 (compared with human insulin) causes precipitation of IGlar upon injection, forming a depot from which it is slowly released.[10] As compared with Neutral Protamine Hagedorn (NPH) insulin, this results in a prolonged action with no pronounced peak effect,[15;16] lower mean FPG levels and lower incidence of nocturnal hypoglycaemia[15-20] whereas within-subject variation in absorption is comparable to that observed with NPH insulin.[21] For further details, please refer to the Summary of Product Characteristics (SPC)[10] and package insert for IGlar and the U.S. Label Information.[11]

7.4 Rationale for the Trial

The main objective was to investigate the safety and efficacy of IDegAsp as add-on to OADs, when glucose levels are no longer controlled by OADs alone. Combined basal-bolus therapy in one injection makes IDegAsp an appropriate once daily (OD) insulin for initiation of insulin treatment in patients with type 2 diabetes mellitus inadequately controlled with OADs. IDegAsp offers an ultra long-acting basal coverage with additional prandial coverage of one meal.

This trial in subjects with type 2 diabetes mellitus will be used to document the efficacy and safety of combined treatment with IDegAsp OD and metformin.

8 Trial Objectives

As stated in the protocol (included in Appendix 16.1.1), the objectives of the trial were as follows:

Primary Objective:

The primary objective was to investigate the long-term safety and tolerability of IDegAsp. This was done by comparing IDegAsp to insulin glargine after 52 weeks of treatment (26 weeks of treatment in trial NN5401-3590 plus 26 weeks of treatment in this extension trial) in terms of the following safety assessments:

- Adverse events
- Hypoglycaemic episodes
- Clinical evaluations
- Central laboratory assessments incl. lipid profile, Cardiovascular (CV) risk markers and insulin antibodies
- Body weight
- Insulin dose

Secondary Objectives:

The secondary objective was to compare the efficacy between IDegAsp and insulin glargine after 52 weeks of treatment in terms of listed efficacy assessments:

- HbA_{1c} (glycosylated haemoglobin) (central laboratory)
- Fasting plasma glucose (FPG) (central laboratory)
- 9-point self-measured plasma glucose profile (9-point profile [SMPG])
- Self-measured plasma glucose (SMPG) for dose adjustments

9 Investigational Plan

9.1 Overall Trial Design and Plan: Description

The main trial (NN5401-3590) was a 26-week, 1:1 randomised, parallel-group trial comparing two active treatment groups in subjects with type 2 diabetes: IDegAsp OD + metformin with that of IGlar OD + metformin, and the present 26-week extension trial was with the same treatment regimen to ensure the most optimal coverage of both basal and bolus requirements. Subjects who consented to participate in the extension trial restarted treatment with IDegAsp OD + metformin or insulin glargine OD + metformin as previously randomly allocated in trial NN5401-3590 after a follow-up period of at least seven days after the main trial during which subjects were treated with NPH insulin to ensure that IDegAsp was washed out when measuring insulin antibodies. Randomisation at the beginning of the main trial was carried out in a 1:1 manner to IDegAsp OD (administered at morning meal) and IGlar OD (administered according to approved labelling).

The main trial included a screening visit and a randomisation visit. The subjects were required to attend further visits and phone contacts during the 26 weeks of treatment, followed by a follow-up period of one week. The extension trial included a screening visit to assess eligibility on the same day as the follow-up visit in the main trial. The subjects were required to attend a further 7 visits and 7 phone contacts during the 26 weeks of treatment, followed by a follow-up visit one week after discontinuing the trial treatment. The total duration of each subject’s participation in the extension trial was approximately 28 weeks (approximately 54 weeks in the main and extension trial combined) (Figure 9–1).

The trial population constituted a population with type 2 diabetes mellitus who would benefit from intensified therapy, applying a treat-to-target concept. The planned number of subjects to be screened and complete the main trial was 752 and 446 respectively. The planned number of subjects to be screened (335) and complete the current extension trial (285) was based on the sample size determination method outlined in Section 9.7.2. All subjects enrolled in the extension trial had previously been participating in the 26-week main trial (NN5401-3590) and received a maximum of 52 weeks of treatment in total.

Trial design – Main trial

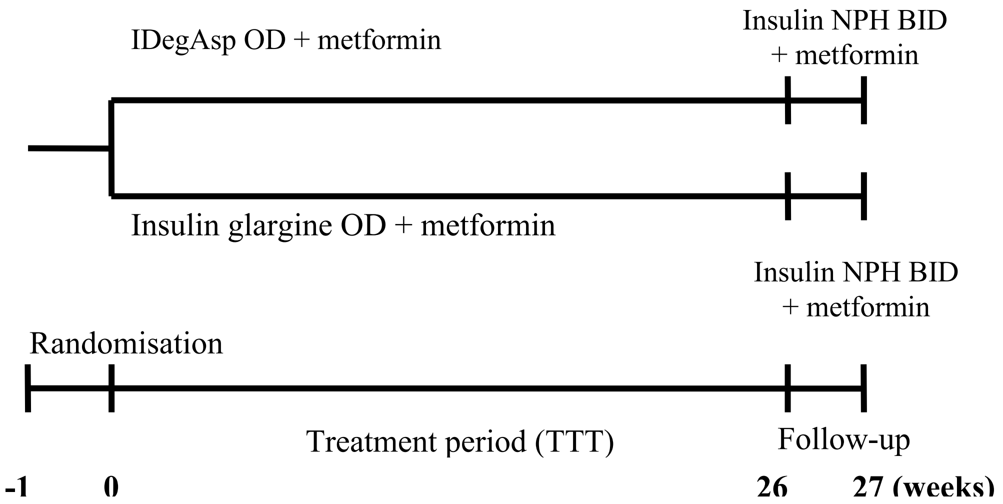

Trial design – Extension trial

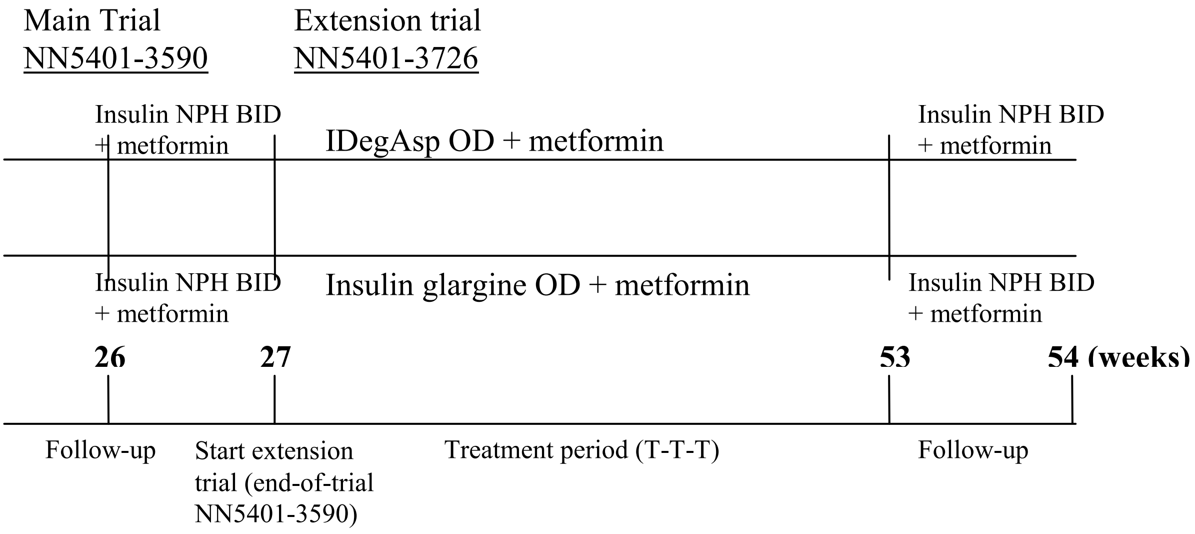

BID = twice daily; IDegAsp = insulin degludec/insulin aspart; NPH insulin = Neutral Protamine Hagedorn insulin; OD = once daily; T-T-T = treat-to-target

Figure 9–1 Trial Design

Please see Section 9.5.1.1 for a detailed flow chart summarising the frequency and timing of visits and the assessments performed at each visit.

No interim analyses were performed in this trial.

An internal Novo Nordisk A/S safety committee performed ongoing safety surveillance during the trial.

The titration of insulin doses was monitored by Quintiles and reviewed by an internal titration committee composed of members from Novo Nordisk A/S, and any significant changes from the titration algorithm were addressed.

9.2 Discussion of Trial Design, Including the Choice of Control Groups

Control Groups

IGlar was chosen as the comparator in this study as it is a commonly used once-daily basal insulin in type 2 diabetes mellitus and has a well known efficacy and safety profile.

Trial Design

This extension trial had the same trial design as in the main trial NN5401-3590, i.e., a parallel, open-labelled and multinational trial design.

An open trial design was chosen, as the comparator product IGlar could not be blinded in an acceptable way. There is no available placebo product for IGlar.

The multinational approach was to ensure that the results were applicable for subjects with different ethnic/racial characteristics.

The total treatment period of 52 weeks (including 26 weeks of treatment in the main trial NN5401-3590 and 26 weeks of treatment in the present extension trial) was expected to be sufficient to confirm the long-term safety of treatment with IDegAsp. Each treatment period of the main trial and the extension trial had a follow-up period of at least seven days to ensure that IDegAsp was washed out when measuring insulin antibodies.

The visit schedule with visits/phone contacts weekly in the main trial and every second week in the extension trial was chosen to ensure:

- Optimal treatment with continued dose adjustment (based on SMPG values) to achieve and maintain glycaemic targets
- Optimisation of compliance

Assignment and Blinding

An open trial design was chosen, as there is no available placebo product for comparator IGlar; therefore, the study could not be blinded in an acceptable way. The subjects were randomised in a 1:1 ratio using an IV/WRS at the beginning of the main trial and there was no further randomisation

in the extension trial period. After completion of the main trial, eligible subjects continued with the same treatment regimen as in the main trial (NN5401-3590).

9.3 Selection of Trial Population

9.3.1 Inclusion Criteria

Inclusion Criteria in the Main Trial

1. Informed consent was obtained before any trial-related activities. (Trial-related activities were defined as any procedure that would not have been performed during standard management of the subject).
2. Male or female ≥ 18 years of age.
3. Type 2 diabetes mellitus (diagnosed clinically) for ≥ 6 months.
4. HbA_{1c} 7.5-11.0% (both inclusive) by central laboratory analysis.
5. Body mass index (BMI) ≤ 40.0 kg/m^2.
6. Insulin-naïve subject (Allowed were: Previous short-term insulin treatment up to 14 days; Treatment during hospitalisation or during gestational diabetes was allowed for periods longer than 14 days).
7. Ongoing treatment with: Metformin and at least one other OAD for at least 3 months prior to randomisation with the minimum doses stated:
   - Metformin:(including fixed combination products) 1500 mg or maximum tolerated dose (at least 1000 mg daily)
   - Insulin secretagogue (sulfonylurea or glinide): Minimum half of the daily maximal dose according to approved labelling
   - DPP-4 inhibitor: Minimum 100 mg daily or according to approved labelling
   - Acarbose: minimum half of the daily maximal dose or maximum tolerated dose
8. Ability and willingness to adhere to the protocol including performance of SMPG profiles according to the protocol.
9. Subject is likely to comply with the investigator's instruction.

Rationale for Inclusion Criteria in the Main Trial

The bullet numbers correspond to the numbering of the inclusion criteria.

1. In accordance with the Helsinki Declaration[1] human subjects should always consent to participation in a clinical trial.
2. As adolescents are not the focus in the current stage of the development program, the inclusion of adolescents is not warranted in accordance with the Helsinki Declaration[1] and ICH guidelines.[2]
3. To include only subjects with an established diagnosis with type 2 diabetes mellitus.

4. An HbA_{1c} <7.0% is the identified goal for good glycaemic control by the ADA. Subjects optimized on current antidiabetic therapy with an $HbA_{1c} \geq 7.0\%$ are considered suitable for additional antidiabetic therapy.
5. Type 2 diabetes mellitus is frequently associated with obesity. In order to include as many subjects from the target population of IDegAsp, a relatively high BMI cut off was therefore selected. Subjects with even higher BMIs are highly insulin resistant, which could create a heterogeneous trial population.
6. To assure that subjects benefited from treatment with once daily insulin. Previous insulin treatment could have caused the development of antibodies against insulin.
7. Metformin is considered a cornerstone in the treatment of type 2 diabetes mellitus. This criterion will ensure that the subjects were getting the most out of the OADs before enrolling in the trial, but still required insulin to lower their HbA_{1c}.
8. Self-explanatory.
9. Self-explanatory.

Inclusion Criteria in the Extension Trial

1. Informed consent obtained before any trial-related activities. (Trial related activities were defined as any procedure that would not have been performed during normal management of the subject)
2. The subject must have completed the 26-week treatment period (Visit 28) in trial NN5401-3590

Rationale for Inclusion Criteria in the Extension Trial

The bullet numbers correspond to the numbering of the inclusion criteria.

1. In accordance with the Declaration of Helsinki, human subjects must consent to participate in a clinical trial.
2. To investigate the long-term safety and efficacy of IDegAsp

9.3.2 Exclusion Criteria

Exclusion Criteria in the Main Trial

1. Anticipated change in concomitant medication known to interfere with glucose metabolism, such as systemic corticosteroids, beta-blockers, monoamine oxidase MAO inhibitors.
2. Use within the last 3 months prior to Visit 1 of GLP-1 receptor agonists and/or thiazolidinediones (TZDs).
3. Anticipated significant lifestyle change during the trial, e.g. shift work (including permanent night/evening shift workers), as well as highly variable eating habits.
4. Cardiovascular disease, within the last 6 months prior to visit 1, defined as: stroke; decompensated heart failure New York Heart Association (NYHA)[22] Class III or IV; myocardial infarction; unstable angina pectoris; or coronary arterial bypass graft or angioplasty.

5. Uncontrolled treated/untreated severe hypertension (systolic BP ≥180 mmHg and/or diastolic BP ≥ 100 mmHg).
6. Impaired liver function, defined as alanine aminotransferase (ALAT) ≥ 2.5 times upper limit of normal (one retest analysed at the central laboratory within a week of receipt or the result was permitted with the result of the last sample being conclusive).
7. Impaired renal function defined as serum-creatinine ≥ 125 µmol/L or ≥ 1.4 mg/dL for males and ≥ 110 µmol/L or ≥ 1.3 mg/dL for females or glomerular filtration rate below 60 mL/minute, calculated by the Cockroft & Gault formula and according to local practise for metformin use (one retest analysed at the central laboratory within a week is permitted with the result of the last sample being conclusive).
8. Recurrent severe hypoglycaemia (more than 1 severe hypoglycaemic event during last 12 months) or hypoglycaemic unawareness as judged by the investigator or hospitalisation for diabetic ketoacidosis during the previous 6 months.
9. Proliferative retinopathy or maculopathy requiring treatment according to the investigator.
10. Pregnancy, breast-feeding, the intention of becoming pregnant or not using adequate contraceptive measures according to local requirements.
11. Cancer and medical history of cancer hereof (except basal cell skin cancer or squamous cell skin cancer).
12. Any clinically significant disease or disorder, except for conditions associated with type 2 diabetes mellitus, which in the investigator's opinion could interfere with the results of the trial.
13. Mental incapacity, psychiatric disorder, unwillingness or language barriers precluding adequate understanding or co-operation, including subjects not able to read or write.
14. Previous participation in this trial. Participation was defined as randomised. Re-screening of screening failures was allowed only once within the limits of the recruitment period.
15. Known or suspected allergy to any of the trial products or related products.
16. Receipt of any investigational drug within 1 month prior Visit 1.
17. Donation of blood or participation in other trials within 1 month prior to Visit 1.
18. Known or suspected abuse of alcohol, narcotics or illicit drugs.

Rationale for Exclusion Criteria in the Main Trial

The bullet numbers correspond to the numbering of the exclusion criteria.

1. Changes in dose of drugs interfering significantly with the insulin requirements were not allowed as they might influence HbA_{1c} (the primary endpoint).
2. GLP-1 receptor agonists and/or thiazolidinediones (TZDs) are either not labelled for concurrent use with insulin or there is evidence that the concurrent use of these drugs with insulin has a higher incidence of AEs. Therefore, a relatively long "wash-out" period of these drugs prior to trial entry was chosen to avoid any carry-over effect on the trial.

3. IDegAsp provides a fixed combination of a basal and a prandial insulin; this regimen was not optimal for this subpopulation and these subjects were therefore excluded. A more flexible regimen than IDegAsp was needed to optimally treat these subjects.
4. To avoid high-risk subjects in which an aggressive insulin titration regimen could be harmful. In addition including a high-risk population could confound the safety results of the trial.
5. Uncontrolled hypertension could confound the safety results of the trial.
6. Liver abnormalities could confound the safety results of the trial.
7. Renal abnormalities could confound the safety results of the trial. In addition, subjects were required to use metformin concomitantly, the use of which is restricted in subjects with renal dysfunction.
8. Patients with recurrent severe hypoglycaemia or hypoglycaemic unawareness are at higher risk of experiencing hypoglycaemia in this treat-to-target trial.
9. This condition could be worsened by an intensified insulin titration regimen.
10. Pregnant subjects were not to be exposed during this Phase 3a trial. Trials focused on this subpopulation might be conducted at a later time.
11. The potential recurrence of these cancers could confound the safety results of the trial. Subjects with basal cell skin cancer or squamous cell skin cancer were allowed as these cancers are not associated with high mortality risk.
12. Potential of confounding the safety results of the trial.
13. The subjects were required to follow trial procedures and needed to have the capacity to do so.
14. Standard exclusion criterion according to GCP.
15. It would not have been ethical to expose subjects with a potential allergy to the trial product.
16. Standard requirement for participation in clinical trials. Any confounding of the efficacy/safety conclusion of the trial was to be avoided.
17. Blood donation could affect HbA_{1c}, which was the key endpoint in the trial.
18. Any confounding of the efficacy/safety conclusions of the trial was to be avoided. Subjects abusing alcohol, narcotics or illicit drugs might not be able to adhere to trial treatment and procedures.

Exclusion Criteria in the Extension Trial

1. Anticipated change in concomitant medication known to interfere significantly with glucose metabolism, such as systemic corticosteroids, beta-blockers, MAO inhibitors.
2. Anticipated significant lifestyle changes during the trial, e.g., shift work (including permanent night/evening shift workers), as well as highly variable eating habits as judged by the investigator.
3. Pregnancy, breast-feeding, the intention of becoming pregnant or not using adequate contraceptive measures according to local requirements.

Subjects who were non-compliant with any of the eligibility criteria, but included in the trial, were to be excluded immediately. If extraordinary circumstances spoke in favour of maintaining the subject in the trial then this was only acceptable if justified and approved by the IEC/IRB, and if the regulatory authorities were notified according to local requirements.

Rationale for Exclusion Criteria in the Extension Trial

The bullet numbers correspond to the numbering of the exclusion criteria.

1. Changes in dose of drugs interfering significantly with the glucose levels were not allowed as they could have influenced HbA_{1c} (the secondary endpoint)
2. For safety reasons since IDegAsp has a prandial insulin component
3. Standard requirement for participation in clinical trials with new chemical or biological entities

9.3.3 Removal of Subjects from Therapy and Assessment

The subject could withdraw from the trial at will at any time.

The subject could be withdrawn from the trial at the discretion of the investigator due to safety concerns or if judged non-compliant with trial procedures.

Withdrawal Criteria in the Main Trial

A subject was to be withdrawn if the following applied:

1. Pregnancy or intention of becoming pregnant.
2. Hypoglycaemia during the treatment period posing a safety problem as judged by the investigator.
3. Major protocol deviation having influence on efficacy or safety data as judged by the investigator.
4. Initiation or significant change of any systemic treatment which in the investigator's opinion could have interfered with glucose metabolism (inhaled corticosteroids were allowed, pausing metformin treatment for a planned radiographic procedure including the use of iodine containing contrast material was allowed).
5. Donation of blood or participation in other trials throughout the trial.
6. Lack of effect: After Week 12, if the subject had not had reduction in HbA_{1c} and had a pre-breakfast SMPG reading > 13.3 mmol/L (> 240 mg/dL) on 3 consecutive days despite appropriate dose adjustments. The subject was to contact the investigator and come in for an unscheduled visit as soon as possible (within 2 weeks). The next scheduled visit was not to be awaited. An FPG was to be obtained and analysed by the central laboratory. If this FPG exceeded 13.3 mmol/L (> 240 mg/dL) and no treatable intercurrent cause for the hyperglycaemia was diagnosed, the subject was to be withdrawn.

Rationale for Withdrawal Criteria in the Main Trial

The bullet numbers correspond to the numbering of the withdrawal criteria.

1. Standard requirement for clinical trials with new chemical or biological entities.
2. Safety issue.
3. Important protocol deviations were to be considered by the investigator if they might have influence on efficacy and safety.
4. Changes in dose of drugs interfering significantly with glucose levels were not allowed as they could have influenced HbA_{1c} (the primary endpoint).
5. Donating blood may lower HbA_{1c} and thus influence key efficacy criteria. It is a standard requirement that participation in another trial was not allowed.
6. Subjects should not be exposed for a long time to ineffective treatment. Twelve (12) weeks reflected clinical practice to allow adequate titration and obtain a valid HbA_{1c} measurement.

Withdrawal Criteria in the Extension Trial

A subject was to be withdrawn in the extension trial if the following applied:
1. Pregnancy or intention of becoming pregnant
2. Hypoglycaemia during the treatment period posing a safety problem as judged by the investigator
3. Major protocol deviation having influence on efficacy or safety data as judged by the investigator
4. Initiation of or significant change to any systemic treatment which in the investigator's opinion could interfere with glucose metabolism (inhaled corticosteroids are allowed)
5. Donation of blood or participation in other trials throughout the trial
6. Lack of effect: not in acceptable glycaemic control when the maximum effects of the treatment regimens are seen, at the discretion of the investigator
7. Lack of effect after week 12 of the extension trial and at any timepoint thereafter, if the subject has not had reduction in HbA_{1c} and has a pre-breakfast SMPG reading > 13.3 mmol/L (240 mg/dL) on three consecutive days despite appropriate dose adjustments. The subject should contact the investigator and come in for an unscheduled visit as soon as possible (within one week). The next scheduled visit should not be awaited. An FPG should be obtained and analysed by the central laboratory. If this FPG exceeds 13.3 mmol/L (>240 mg/dL) and no treatable intercurrent cause for the hyperglycaemia has been diagnosed, the subject must be withdrawn.

Rationale for Withdrawal Criteria in the Extension Trial

The bullet numbers correspond to the numbering of the withdrawal criteria.

1. Standard requirement for clinical trials with new chemical or biological entities
2. Safety issue
3. Important protocol deviations had to be considered by the investigator if they had influence on efficacy and safety

4. Changes in dose of drugs interfering significantly with the glucose levels were not allowed as they could have influenced HbA_{1c}
5. Donating blood could have lowered HbA_{1c} and thus influenced key efficacy criteria. It is a standard requirement that participation in another trial was not allowed
6. Subjects should not be exposed for a long time to ineffective treatment. Twelve (12) weeks reflected clinical practice to allow adequate titration and obtain a valid HbA_{1c} measurement
7. Subjects should not be exposed for a long time to ineffective treatment. Twelve (12) weeks reflected clinical practice to allow adequate titration and obtain a valid HbA_{1c} measurement

If a subject was prematurely withdrawn from the trial the investigator had to ensure that the procedures for Visit 44 (end-of-treatment visit) and Visit 45 (follow-up visit) were undertaken, if possible. The same procedure also applied for subjects withdrawn during the main trial.

Withdrawn subjects were not replaced. Predicted withdrawal rate was 15%; see 9.7.2.

9.4 Treatments

9.4.1 Treatments Administered

During the main trial, all subjects randomised to IDegAsp were instructed to initiate treatment with IDegAsp 10 U OD with breakfast (morning meal).

During the main trial, all subjects randomised to IGlar were instructed to initiate treatment with IGlar 10 U OD according to approved labelling. Both IDegAsp and IGlar were injected subcutaneously (s.c.) either in the abdomen, upper arm (deltoid area) or thigh. Rotation of injection sites within a given region was recommended.

At Visit 28 (end of treatment) in the main trial, subjects were instructed to switch basal insulin treatment to the intermediate-acting NPH insulin injected BID (morning and evening) until the follow-up visit (Visit 29).

All subjects who completed the main trial NN5401-3590 (Visit 28) and were found eligible for the present extension trial (Visit 30) were encouraged to participate. The antidiabetic treatment (NPH insulin + metformin) that subjects received during the follow-up period of the main trial was discontinued when entering the extension trial and subjects were instructed to restart treatment with either IDegAsp OD + metformin or IGlar OD + metformin according to what they were randomised to in the main trial (NN5401-3590). Subjects were instructed to restart treatment with their respective trial insulin products preferably at the same dose levels as reported at end of treatment in the main trial. During the extension, a protocol amendment (substantial amendment 2) permitted IDegAsp to be taken either with breakfast or with the largest meal. However, most countries did not

obtain approval/agreement for this amendment until between September and November 2010 and one country (Russia) obtained approval/agreement only two months before the trial end, in March 2011. IGlar was again administered according to approved labelling. The dose of insulin was adjusted according to the current dose levels, the treatment response and most recent SMPG measurements obtained. The insulin dose adjustments were to follow the titration guidelines stated in the protocol. A central titration committee at Novo Nordisk A/S followed up on repeated deviation from the titration algorithm.

At Visit 44 (end of treatment), subjects were instructed to switch basal insulin treatment to the intermediate-acting NPH insulin injected BID + metformin during the follow-up period before measurement of antibodies. NPH insulin was also to be injected in the abdomen, upper arm (deltoid region) or thigh, with rotation of injection sites within a given region recommended.

9.4.2 Identity of Investigational Products

The trial products during the treatment were as follows:

- IDegAsp 100 U/mL, 3 mL PDS290 pen (The IDegAsp drug product consists of two drug substances (70 volume% insulin degludec + 30 volume% insulin aspart) 100 U/mL)
- IGlar (Lantus®, Sanofi-Aventis, U.S) 100 U/mL, 3 mL SoloStar™
- NPH (Insulatard®, Protaphane®, Novolin N™) insulin 100 IU/mL, 3 mL FlexPen®

Novo Nordisk A/S, Denmark, provided the trial products. For batch numbers for each subject, refer to Appendix 16.1.6.

Metformin was not considered a trial product and was not supplied by Novo Nordisk A/S.

Details of the preparations, including batch numbers, are shown in Table 9–1.

Table 9–1 Trial Product

Trial Product	Dose	Batch Number	Expiry Date
IDegAsp	100U/ml 3ml	YP50611	26-May-2011
Glargine	100IU/ml 3ml	40C764	31-May-2012
Glargine	100IU/ml 3ml	40C368	29-Feb-2012
Glargine	100IU/ml 3ml	40C408	31-Mar-2012
Glargine	100IU/ml 3ml	40C777	30-Jun-2012
Glargine	100IU/ml 3ml	OF166A	31-Dec-2012
NPH	100 CCH 3 ml	YP50831	12-Aug-2012

IDegAsp consists of two drug substances (70 volume% insulin degludec + 30 volume% insulin aspart)

IDegAsp was administered via the PDS290 insulin delivery device, NPH insulin was administered via the FlexPen® insulin delivery device, and insulin glargine was administered via the SoloStar™ insulin delivery device

Cross-reference: Appendix 16.1.6

All insulin products were stored between 2°C and 8°C and were not allowed to freeze. The investigator was to ensure the availability of proper storage conditions and keep a temperature log to document the correct temperature interval. The temperatures during storage were to be monitored by a calibrated, stationary and continuously recording system. In case of storage outside the temperature range the investigator was to contact the monitor. Trial products stored outside the temperature range were not to be used and were to be stored separately within allowed temperature ranges until after evaluation of the deviation by Novo Nordisk A/S. Fifteen (15) minutes outside the indicated range was considered negligible and allowed, and was not to be recorded as a deviation. Returned trial products (partly used or unused including empty packaging material) were to be stored separately from non-allocated trial products.

Once opened (i.e., during use), IGlar and NPH insulin were to be stored according to approved labelling. IDegAsp was to be kept at a temperature below 30°C and was not to be refrigerated. Insulin preparations (both not in-use and in-use) were not exposed to excessive heat or direct sunlight. The insulin products were only to be used if they appeared clear and colourless.

Trial products were packaged and labelled by Novo Nordisk A/S, Denmark. Labelling was in accordance with local law and trial requirements. Each investigational site was supplied with sufficient trial products for the trial on an ongoing basis controlled by the IV/WRS. Dispensing units were prepared and distributed to the sites according to enrolment and randomisation. Note that the trial product labelling was different from the labelling of the marketed or to-be-marketed products.

The subjects were provided with directions for the use of IDegAsp, IGlar, and NPH insulin. These were distributed separately by the investigator according to the flow charts (Table 9–4 and Table 9–5). The distribution was documented in the subject notes.

None of the insulin products were used after the expiry date.

Auxiliary Supply

The following were supplied by Novo Nordisk A/S:

- Needles (8 mm) for insulin pre-filled pen system
- Blood glucose meters including lancets, plasma-calibrated test strips and control solutions

9.4.3 Method of Assigning Subjects to Treatment Groups

During the main trial, the treatment was open-labelled. Randomisation was carried out in a 1:1 manner using IV/WRS to either IDegAsp or IGlar, both in combination with metformin.

The randomisation scheme can be found in Appendix 16.1.7.

There was no further randomisation in the extension trial. Subjects participating in this extension trial were instructed to restart the same treatment regimen to which they were allocated in the main trial.

9.4.4 Selection of Doses in the Trial

At randomisation (Visit 2), the investigator was instructed to initiate treatment with 10 U of IDegAsp or IGlar. All subjects started on a once daily insulin treatment regimen. IDegAsp was to be given with breakfast (even if this meal was very small). IGlar was to be given according to approved labelling.

In the extension trial, subjects were instructed to restart treatment with respective trial insulin products preferably at the same dose levels (same number of units) as reported at end of treatment (Visit 28) in trial NN5401-3590. There was possibility for dose adjustment at the discretion of the investigator taking into consideration the dose levels, treatment response and most recent SMPG measurements obtained during treatment with NPH insulin in the follow-up period.

9.4.5 Selection and Timing of Dose for Each Subject

In general, the same treatment algorithms as specified were applied to both the main trial and the extension trial regarding the titration of trial insulin products. Furthermore, subjects were allowed to adjust insulin doses in between visits according to individual requirements. No maximum dose of any insulin was specified.

Insulin Degludec/Insulin Aspart or Insulin Glargine

During the extension trial period, subjects who had been randomised to IDegAsp during the main trial period were to inject IDegAsp OD with breakfast (morning meal) or the largest meal and the subjects who had been randomised to IGlar were to inject IGlar OD according to approved labelling. An increase in the dose of IDegAsp or IGlar was done based on the mean pre-breakfast PG from the preceding 3 days using the titration algorithm Table 9–2. If a subject had only

measured PG values on 2 of the last 3 days prior to the contact, titration of the insulin dose was to be based on these values. If a measurement was only available for one day, this was used to assess the need for insulin dose adjustment at the discretion of the investigator.

Table 9–2 Titration Algorithm for Insulin Degludec/Insulin Aspart or Insulin Glargine Doses

Mean pre-breakfast Plasma Glucose		Adjustment of Insulin Degludec/Insulin Aspart or Insulin Glargine U
mmol/L	mg/dL	
< 5.0	< 90	No adjustment
< 7.0	< 126	+ 2
< 8.0	< 144	+ 4
< 9.0	< 162	+ 6
≥ 9.0	≥ 162	+ 8

If low PG values occurred without an obvious explanation, reduction of IDegAsp or IGlar dose was to be performed according to Table 9–3.

Table 9–3 Reduction of Insulin Degludec/Insulin Aspart or Insulin Glargine Doses

Pre-breakfast Plasma Glucose		Adjustment of Insulin Degludec/Insulin Aspart or Insulin Glargine U
mmol/L	mg/dL	
< 3.1 without obvious explanation	< 56 without obvious explanation	- 4 (for a dose of >45 U, suggest a dose reduction of 10%)
< 3.9 without obvious explanation	< 70 without obvious explanation	- 2 (for a dose of >45 U, suggest a dose reduction of 5%)

Neutral Protamine Hagedorn Insulin

From end of trial insulin treatment (Week 26) to the follow-up period before measurement of antibodies, the subjects discontinued all trial insulin and were switched to NPH insulin. Since insulin NPH insulin is intermediate-acting insulin, it was to be administered twice a day. The first dose of NPH insulin was to be given 24 h after the last dose of IDegAsp or IGlar. To determine the dose of NPH insulin to be taken during the follow-up period, the total daily basal dose at end of the treatment period was to be reduced by 20% and divided by 2 to be administered morning and evening. The NPH insulin dose was adjusted after a few days treatment in order to approach the total daily insulin dose administered at the end of the treatment period.

9.4.6 Blinding

This was an open-label trial and there was no blinding of investigators and subjects. An open trial design was chosen as the comparator product, IGlar, could not be blinded in an acceptable way.

The internal Novo Nordisk safety committee and staff and the independent EAC were blinded. The safety committee could recommend unblinding of any data for further analysis. In case this possibility occurred, an independent ad hoc group was to be established to maintain the blinding.

9.4.7 Prior and Concomitant Therapy

Concomitant illness

Concomitant illness was defined as any illness that was present at the start of the trial (i.e., at the first visit).

Concomitant medication

Concomitant medication was defined as any medication other than the trial product(s) that was taken during the trial, including the screening periods.

Details of all concomitant illnesses, medical history and concomitant medication in the main trial were to be recorded at trial entry (i.e., at Visit 1). Details of all concomitant illnesses and medication were recorded at extension trial entry (at Visit 30). Adverse events that were not yet recovered by the end of the main trial NN5401-3590 were not regarded as concomitant illnesses in the extension trial. Concomitant medication recorded during trial NN5401-3590 which was still taken at start of the extension trial was transcribed to a concomitant medication form for the extension trial by the investigator. Any changes in concomitant medication were recorded at each visit. If the change influenced the subject's eligibility to continue in the trial, the sponsor was informed. Concomitant illnesses and medication recorded at main trial screening visit were used as baseline.

Details of all medical history and allergy towards medication and or food, pollen or other types of allergies were recorded at trial entry.

9.4.8 Treatment Compliance

At each visit the investigator was to emphasise the necessity for the subject to adhere to trial procedures in order to encourage subject compliance. In addition, subject compliance was to be assessed by monitoring of drug accountability. The unused amount of trial product was to be assessed against the dispensed amount and, in case of discrepancies; the subject was to be asked.

Substantial failure to comply with the prescribed insulin dosage regimen could lead to withdrawal. In addition, the investigator assessed the compliance of the subject at each visit based on a review of glycaemic control, adherence of the visit schedule, and completion of the subject's diary including the SMPG profiles. If a subject was discovered to be non-compliant, the investigator was to inform the subject of the importance of taking trial products as directed.

9.5 Efficacy and Safety Variables

9.5.1 Efficacy and Safety Measurements Assessed and Flow Chart

9.5.1.1 Flow Chart

The main trial consisted of a screening visit (Visit 1), a randomisation visit (Visit 2), a 26-week treatment period (Visit 3 to Visit 28) after which a follow-up visit (Visit 29) was to be performed.

Procedures for the scheduled visits and phone contacts are described in the section below and in the flowchart Table 9–4.

Table 9–4 Trial Flow Chart- Main Trial

	Screen	Rand	0-26 weeks												FU
**Visit Number (V)**	**V1**	**V2**	**V3**	**V4**	**V6**	**V8**	**V10**	**V12**	**V14**	**V16**	**V18**	**V22**	**V26**	**V28**	**V29**
☎ Phone Contact number (P)[1] (For details see separate flowchart below)	P5 P7 P9 P11 P13 P15 P17 P19 P23 P27<br>P20 P24<br>P21 P25														
**Time of visit (weeks)**	**-1[2]**	**0**	**1**	**2**	**4**	**6**	**8**	**10**	**12**	**14**	**16**	**20**	**24**	**26**	**27[4]**
Visit window (days)			±3	±3	±3	±3	±3	±3	±3	±3	±3	±3	±3	−3	+5
**SUBJECT RELATED INFO/ASSESSMENTS**															
Informed consent	x														
Inclusion/Exclusion criteria	x	x													
Randomisation		x													
Withdrawal criteria			x	x	x	x	x	x	x	x	x	x	x	x	
Demography	x														
Diagnosis of diabetes	x														
Concomitant ill / medical history	x														
Concomitant medication	x	x	x	x	x	x	x	x	x	x	x	x	x	x	x
Height	x														
Body weight	x	x[3]							x[3]		x[3]			x[3]	
**EFFICACY**															
Fasting plasma glucose[3]		x							x		x			x	
HbA_{1c}	x	x							x		x			x	
1-point SMPG profile[5]		x	x	x	x	x	x	x	x	x	x	x	x	x	x
9-point SMPG profile[6]		x							x		x			x	
CGM profile -at selected trial sites[7]		x												x	
β-cell function[3]		x												x	
**SAFETY**															
Adverse events		x	x	x	x	x	x	x	x	x	x	x	x	x	x
Insulin antibodies[3]		x							x					x	x
NT-proBNP		x												x	
hsCRP[3]		x												x	
Biochemistry	x													x	

	Screen	Rand	0-26 weeks												FU
**Visit Number (V)**	**V1**	**V2**	**V3**	**V4**	**V6**	**V8**	**V10**	**V12**	**V14**	**V16**	**V18**	**V22**	**V26**	**V28**	**V29**
Phone Contact number (P)[1] (For details see separate flowchart below)	P5 P7 P9 P11 P13 P15 P17 P19 P20 P21 P23 P24 P25 P27														
**Time of visit (weeks)**	**-1[2]**	**0**	**1**	**2**	**4**	**6**	**8**	**10**	**12**	**14**	**16**	**20**	**24**	**26**	**27[4]**
Visit window (days)			±3	±3	±3	±3	±3	±3	±3	±3	±3	±3	±3	−3	+5
Haematology	x													x	
Lipids[3]		x												x	
Urinalysis	x													x	
Pregnancy test[8]	x													x	
Albumin/ creatinine ratio		x												x	
ECG – 12 lead	x[9]													x	
Eye examination (fundusc/-photo)	x[9]													x[10]	
Hypoglycaemic episodes		x	x	x	x	x	x	x	x	x	x	x	x	x	x
Hypo event questionnaire[11]		x	x	x	x	x	x	x	x	x	x	x	x	x	x
Physical examination	x													x	
Vital signs	x													x	
**OTHER ASSESSMENTS**															
PRO questionnaires[12]		x							x					x	
End of trial															x
**TRIAL MATERIAL**															
IV/WRS call	x	x			x		x				x			x	x
Drug accountability (IV/WRS)					x		x				x			x	x
Dispensing visit		x			x		x				x			x	
Dispense package insert		x			x		x				x			x	
First date and dose on trial insulin			x												
Date and dose of trial insulin, on 3 days before visit			x	x	x	x	x	x	x	x	x	x	x	x	
New dose of trial insulin		x	x	x	x	x	x	x	x	x	x	x	x		
Last date and dose on trial insulin														x	
Insulin NPH dose [13]															x
**REMINDERS**															
Attend visit fasting		x							x		x			x	x
Notes made if change in OAD dose or treatment			x	x	x	x	x	x	x	x	x	x	x	x	
Make appointment for Eye examination at the end of the trial										x					
Handling/training of product/device[14]		x	x											x	
Instruction//handout of glucose meter[14]	x														
Hand-out/instruction diary	x	x	x	x	x	x	x	x	x	x	x	x	x	x	
Sign off casebook															x

CGM = continuous glucose monitoring; ECG = electrocardiogram; EOT = end of trial; FU = follow-up; HbA_{1c} = glycosylated haemoglobin; hsCRP = high sensitive C-reactive protein; NT-proBNP = B-type natriuretic peptide; OAD = oral antidiabetic drug; SMPG =self-measured plasma glucose; IV/WRS = interactive voice/web response system; NPH = Neutral Protamine Hagedorn; PRO = patient reported outcome

[1] A phone contact could be converted to a visit if for instance further titration attention was needed.

[2] Screening took place approximately 1 week prior to randomisation.

[3] Fasting visits: The subjects attended Visits 2, 14, 18, 28 and 29 fasting, having consumed only water since midnight for measurement of hsCRP, lipids (Visits 2 and 28), fasting plasma glucose (Visits 2, 14, 18 and 28) and insulin antibodies (Visits 2, 14, 28, 29). No diabetes medication was allowed before these visits. If the subjects attended the site in a non-fasting condition the visit was to be re-scheduled within the next 2 working days.

[4] Follow-up visits were to take place at least 7 days after the actual date Visit 28 was performed.

[5] 1-point SMPG measurements were to be taken on the 3 consecutive days just before Visits 2-29. Measurements, in both treatments arms, were to be taken before breakfast.

[6] 9-point profiles (SMPG) were to be started in the morning 2 days before Visits 2, 14, 18 and 28. Measurements were to be performed before and after (90 minutes after the start of the meal) breakfast, lunch, main evening meal, before bedtime, at 4 am and before breakfast on the following day. Please note that the 9-point profile (SMPG) included the measurement before breakfast on Day 3.

[7] CGM: Was to be performed in subjects at selected trial sites.

[8] Pregnancy test: At Visits 1 and 28 a blood pregnancy test was to be performed in women of child bearing potential. During the trial a urine pregnancy test was to be performed if a menstrual period was missed or pregnancy was suspected. If at phone contacts, a subject reported missed menstrual period, the subject was to attend the site for a urine pregnancy test. [For Austria only: Urine pregnancy tests were to be performed once a month at Visits 6, 10, 14, 18, 22 and 26).

[9] ECG and fundoscopy/fundusphotography performed within 8 weeks before Visit 2 were acceptable if results were available at the visit.

[10] Fundoscopy/fundusphotography performed within a period of 3 weeks before Visit 28 was acceptable if results were available at the visit.

[11] A hypoglycaemia event questionnaire was to be used at all visits, but only if the subject reported to have experienced one or more hypoglycaemic events (symptomatic or asymptomatic).

[12] PRO questionnaires; Diabetes Medication Satisfaction Questionnaire (DiabMedSat), Diabetes Productivity Measure Questionnaire (DPM), Health Related Quality of Life Questionnaire (SF-36) and Treatment Related Impact Measure-Diabetes (TRIM-D). This battery of PRO questionnaires evaluated health-related quality of life, treatment satisfaction and preferences. The PRO questionnaires were to be filled in by the subject at the visits, preferably before any other trial-related procedures in a confidential manner (the answers were to be kept strictly confidential, even from the trial site personnel).

[13] NPH insulin therapy; dose of non-trial diabetic therapy during Days 1, 3, and 5 after Visit 28 and on the day just before Visit 29.

[14] Instruction was to be done at the start of the trial and if required.

The extension trial consisted of a screening visit (Visit 30), a 26-week treatment period (Visit 31 to Visit 44) after which a follow-up visit (Visit 45) was to be performed. The screening visit of the extension trial took place on the same day as the follow-up visit in the main trial NN5401-3590. Procedures for the scheduled visits and phone contacts in extension trial period were described in the section below and in the flow chart (see Table 9–5).

If a planned phone contact or in-person visit for some reason was not performed according to the visit schedule, the investigator was to ensure that the phone contact or in-person visit was performed within visit window of the originally planned contact.

Table 9–5 Trial Flow Chart- Extension Trial

**Trial NN5401-3726 Type 2**	**Screen**	**0-26 weeks**							**FU**
**Visit Number (V)**	**V30[0]**	**V33**	**V35**	**V37**	**V39**	**V41**	**V43**	**V44**	**V45**
Phone Contact number (P)[2] (For details see separate flowchart below)	P31 P32 P34 P36 P38 P40 P42								
**Time of visit[4] (weeks)**	**27[0]**	**31**	**35**	**39**	**43**	**47**	**51**	**53**	**54[3]**
Visit window (days)		±3	±6	±6	±6	±6	±6	-3	+7
**SUBJECT RELATED INFO/ASSESSMENTS**									
Informed consent[5]	x								
In/Exclusion criteria	x								
Withdrawal criteria		x	x	x	x	x	x	x	
Concomitant medication	x[6]	x	x	x	x	x	x	x	x
Body weight[7]	x			x				x	
**EFFICACY**									
Fasting plasma glucose[7]	x			x				x	
HbA_{1c}	x			x				x	
1-point profile[8]		x	x	x	x	x	x	x	x
9-point profile[9]				x				x	
**SAFETY**									
Adverse events[10]	x[11]	x	x	x	x	x	x	x	x
Insulin antibodies[7]				x				x	x
NT-proBNP								x	
hsCRP[7]								x	
Biochemistry								x	
Haematology								x	
Lipids[7]								x	
Urinalysis								x	
Pregnancy test[13]	x							x	
Urine Albumin/creatinine ratio								x	
ECG – 12 lead								x	
Eye examination (fundusc/-photo)	x[12]							x[14]	
Hypoglycaemic episodes		x	x	x	x	x	x	x	x
Physical examination								x	
Vital signs								x	
**OTHER ASSESSMENTS**									
End of trial									x[15]
**TRIAL MATERIAL**									
IV/WRS call	x			x				x	x
Drug accountability (IWRS)				x				x	x
Dispensing visit	x			x				x	
Dispense Directions for Use/local package insert	x			x				x	
First date and dose on trial insulin (insulin NPH)									x
Date and dose of trial insulin, on 3 days before visit		x	x	x	x	x	x	x	

Trial NN5401-3726 Type 2	Screen	0-26 weeks							FU
**Visit Number (V)**	**V30[0]**	**V33**	**V35**	**V37**	**V39**	**V41**	**V43**	**V44**	**V45**
Phone Contact number (P)[2] (For details see separate flowchart below)	P31 P32 P34 P36 P38 P40 P42								
**Time of visit[4] (weeks)**	**27[0]**	**31**	**35**	**39**	**43**	**47**	**51**	**53**	**54[3]**
Visit window (days)		±3	±6	±6	±6	±6	±6	-3	+7
New dose of trial insulin	x	x	x	x	x	x	x	x	
Last date and dose on trial insulin (SIAC, insulin glargine or insulin NPH)								x	x
Date and dose of insulin NPH [16]									x
**REMINDERS**									
Attend visit fasting	x[17]			x				x	x
Make appointment for Eye examination at EOT (V44)				x					
Handling/training of product/device[18]	x							x	
Instruction//handout of glucose meter[19]	x								
Hand-out/instruction diary	x	x	x	x	x	x	x	x	
Sign off case book									x

1. Start of extension trial NN5401-3726 screening visit (Visit 30) is the same date as trial NN5401-3590 follow-up visit (Visit 29)
2. A phone contact may be converted to a visit if, for instance, further titration attention is needed.
3. Follow-up visit (Visit 45) must not be performed earlier than seven days after end of treatment (Visit 44).
4. Time of visit is always calculated in relation to the actual date of the screening visit (Visit 30).
5. The informed consent process including obtaining informed consent may take place before the screening visit (Visit 30).
6. Concomitant medication recorded during trial NN5401-3590 which is still being taken at start of trial NN5401-3726 must be transcribed to a concomitant medication form for trial NN5401-3726.
7. Fasting visits: The subjects must attend Visits 30, 37, 44 and 45 fasting, having consumed only water since midnight for measurement of hsCRP and lipids (visit 44), weight and fasting plasma glucose (Visits 30, 37 and 44) and insulin antibodies (Visits 37, 44 and 45). No diabetes medication is allowed on the day of the visit before the blood sample has been drawn. If the subjects attend the site in a non-fasting condition the visit should be re-scheduled within the next two working days.
8. 1-point SMPG measurements should preferably be taken on the three consecutive days just before phone contacts 31-Visit 45. Measurements, in both treatments arms, are to be taken before breakfast.
9. 9-point profiles (SMPG) must be started in the morning 2 days before Visits 37 and 44. Measurements are to be performed before and after (90 min after the start of the meal) breakfast, lunch, main evening meal, before bedtime, at 4 a.m. and before breakfast on the following day. Please note that the 9-point profile is overlapping with the 1-point profile.
10. Adverse events should not be linked between trial NN5401-3590 and NN5401-3726. Adverse events that are not yet recovered by the end of trial NN5401-3590 should be followed up on NN5401-3590 AE follow-up forms.
11. Adverse events will be collected from trial NN5401-3726 screening visit (Visit 30) and not from time of informed consent.
12. At the screening visit (Visit 30) fundoscopy/fundusphotography should only be performed if no data is available from NN5401-3590 Visit 28.
13. Pregnancy test: at Visit 30 a urine pregnancy test is performed in women of child bearing potential. At Visit 44 a blood pregnancy test will be performed in women of child bearing potential. During the trial a urine pregnancy test will be performed if a menstrual period is missed or whenever pregnancy is suspected. If at phone contacts, subjects report missed menstrual period, the subject will have to attend the site for a urine pregnancy test. [For Austria only: urine pregnancy tests will be performed once a month at Visits 30, 33, 37 and 41].
14. Fundoscopy/fundusphotography performed within a period of three weeks before end-of-treatment visit (Visit 44) is acceptable if results are available at the visit.

15. If a subject is discontinued in trial NN5401-3726 due to an ongoing adverse event from trial NN5401-3590, the primary reason for discontinuation must be entered under 'Other' in the End of Trial form.
16. Total daily dose and date of insulin NPH during day 1, 3, and 5 after Visit 44 (end-of-treatment visit) and on the day just before Visit 45 (follow-up visit).
17. Subjects will be fasting according to trial NN5401-3590 follow-up visit (Visit 29) schedule.
18. Instruction to be done at the start of the trial and if required.
19. Should only be supplied if the blood glucose meter from NN5401-3590 can not be re-used.

Screening Visit in Main Trial (Visit 1)

Screening took place approximately 1 week prior to randomisation. Before screening took place, subjects were provided with written information about the trial and the procedures involved, in accordance with local requirements. Subjects were fully informed, orally and in writing, about their responsibilities and rights while participating in the trial, as well as of possible advantages/ disadvantages when being treated with the trial medication (IDegAsp or IGlar). Subjects who wished to participate in the trial signed and dated the informed consent form for the trial before any trial-related procedures. All subjects were provided with a copy of their own signed and dated Informed Consent Form(s).

At Visit 1, information on type and daily dose of current OADs was to be recorded at the concomitant medication form in the eCRF. In addition, OAD doses were to be recorded in the diary before each visit. Subjects were to continue on their current diabetes treatment until the randomisation visit (Visit 2), and were not supplied with any trial medication until then.

At screening, the subjects were assigned unique subject identification (ID) which remained the same throughout the trial. The subject ID consisted of 6 digits (the first 3 digits indicated trial site number and the last 3 digits indicated subject number).

The following were to be performed and/or recorded in the eCRF in addition to what was already described Table 9–4.

- Signed Informed Consent (s), date and time
- Demography:
  - Date of birth
  - Sex
  - Race
  - Ethnicity
- Diagnosis of diabetes mellitus:
  - Date of diagnosis of diabetes mellitus
- Diabetes treatment history (concomitant medication):
  - Current diabetes treatment (prior to the screening visit and until randomisation)
  - Dose of current diabetes treatment
  - Start date of current diabetes treatment

Screening Failures

If the subject was ineligible to participate in the trial the subject was considered a screening failure. Consequently a screen failure call was to be performed using the IV/WRS, a screening failure form was to be filled in and the affirmation statement form (case book) signed in the eCRF. Serious and non-serious AEs (SAEs) from screening failures were to be entered by the investigator into the eCRF and then transferred to the clinical database.

Re-screening of screening failures was allowed only once within the limits of the recruitment period. In case of re-screening, a new informed consent was to be obtained, a new subject ID was to be allocated and all samples and assessments were to be performed once more, except for fundoscopy/fundusphotography and electrocardiogram (ECG).

Randomisation Visit (Visit 2) to Main Trial Follow-up Visit

For randomisation visit (Visit 2) to follow-up visit (Visit 29), please see Table 9–4.

At randomisation, the subject's previous OAD diabetes treatment was discontinued except for metformin. Subjects treated with a fixed combination of metformin and dipeptidyl peptidase-4 (DPP-4) inhibitors discontinued their treatment with DPP-4 inhibitors and changed to treatment with metformin at a dose level as close to their previous regimen as possible.

Screening Visit in the Extension Trial (Visit 30)

Before screening took place, subjects were provided with written information about the trial and the procedures involved, in accordance with local requirements. Subjects were fully informed, orally and in writing, of their responsibilities and rights while participating in the trial, as well as of possible advantages/disadvantages when being treated with the trial medication (IDegAsp + metformin or IGlar + metformin). Subjects had the opportunity to ask questions, and had ample time to consider participation. Subjects who wished to participate in the trial then signed, dated, and recorded the clock time on the informed consent for the trial before any trial-related procedures took place. The informed consent process including obtaining informed consent may have preferably taken place during trial NN5401-3590 and at the latest at the screening visit (Visit 30) before any trial-related procedures took place.

All subjects were provided with a copy of their own signed and dated inform consent form.

If the investigator was not the subject's primary physician, the investigator was to preferably notify the primary physician about the subject's trial participation. If required, permission was given by the subject.

Subjects were identified by the same subject number given in trial NN5401-3590. At screening, the subjects were re-assigned the same unique subject identification (ID) which remained the same

throughout the trial. The subject ID consisted of six digits (the first three digits indicating site number and the last three digits indicating subject number).

Screening Visit (Visit 30) to Follow-up Visit (Visit 45) in Extension Trial

From extension trial screening visit (Visit 30) to follow-up visit (Visit 45), refer to Table 9–5 for further detailed procedure.

Concomitant medication recorded during trial NN5401-3590 which was still being taken at the start of trial NN5401-3726 was to be transcribed to a concomitant medication form for trial NN5401-3726.

In accordance with requirements for both follow-up visit (Visit 29) in trial NN5401-3590 and screening visit (Visit 30) in this trial, subjects were to attend the screening visit (Visit 30) fasting, meaning having consumed only water since midnight and without taking any insulin (insulin NPH) in the morning.

At the screening visit (Visit 30) fundoscopy/fundusphotography was only to be performed if no data was available from NN5401-3590 Visit 28.

At Phone Contact 31, information on first date and dose on trial product (insulin degludec/insulin aspart or insulin glargine) in trial NN5401-3726 was recorded.

At Visit 44 (end-of-treatment) information on last date and dose on trial products (insulin degludec/insulin aspart or insulin glargine) was recorded.

At Visit 44 (end-of-treatment) subjects were instructed to switch basal insulin treatment to the intermediate-acting insulin NPH until the follow-up visit (Visit 45).

This was done in order to provide basal insulin coverage while reducing the level of exogenous insulin present at antibody sampling and consequently to reduce the possibility for interference with antibody measurements. The first dose of insulin NPH was to be given 24 hours after last dose of IDegAsp/insulin glargine.

Visit 45 (follow-up visit) was not to be performed any earlier than 7 days after the end-of-treatment visit. All subjects were to attend the follow-up visit fasting and without having taken any insulin (insulin NPH) in the morning on the day of the follow-up visit.

At Visit 45 (follow-up visit) information on date of first and last injection of insulin NPH was recorded. In addition information on date and total daily dose of insulin NPH at day 1, 3, and 5 after Visit 44 (end-of-treatment visit) and on the day just before Visit 45 (follow-up visit) was recorded by the subject in the diary.

If a subject was prematurely withdrawn from the trial, the investigator was to ensure that the procedures for Visit 44 (end-of-treatment visit) and Visit 45 (follow-up visit) were undertaken. Subjects were switched to NPH insulin + metformin at the end-of-treatment visit until the follow-up visit (if these were done on different days).

9.5.1.2 Efficacy Assessments

The following efficacy variables were assessed in both the main trial and the extension trial:

- HbA_{1c}
- FPG
- SMPG
  - prebreakfast SMPG profile
  - 9-point SMPG profile with 1 additional measurement before breakfast

The following efficacy variables were only assessed in the main trial:

- PRO questionnaires
- CGM (at selected sites)
- Beta-cell function after 26 weeks of treatment
- Hypoglycaemic Episode – Interview Questionnaire

HbA_{1c}

Blood samples for HbA_{1c} were to be collected and analysed at Visits 1, 2, 14, 18, and 28 in the main trial and at Visits 30, 37, and 44 in extension trial. The assay method used was a National Glycohaemoglobin Standardization Program certified method, refer to Appendix 16.1.10.

FPG

FPG samples were collected at Visits 2, 14, 18 and 28 in the main trial and at Visits 30, 37 and 44 in the extension trial. The subjects had to attend these visits fasting.

Self-Measured Plasma Glucose

At Visit 1 in the main trial and Visit 30 in the extension trial, subjects were supplied with a glucose meter and instructions on use of the device including regular calibration according to the manufacturer's instructions (Appendix 16.1.1).

Subjects were instructed in how to record the results of the SMPG values in the diaries provided and only recorded the plasma glucose values based on glucose meter measurements. The record of each plasma glucose measurement included date and plasma glucose value.

1-point profiles (SMPG)

Subjects were asked to perform SMPG measurements before breakfast. Measurements were preferably to be performed on three consecutive days just before each scheduled visit or phone

contact using the glucose meter provided. SMPG measurements before breakfast were preferably performed after having consumed only water since midnight, and before insulin injection. These measurements were required for optimal insulin dose adjustment and maintenance as described in the Insulin Titration Guideline, Protocol Appendix C (Appendix 16.1.1).

It is important that SMPG measurements were recorded on the same days as the insulin doses were recorded.

9-point profile (SMPG) with additional 1-point profiles (SMPG)

Subjects were instructed to perform a 9-point profile (SMPG) with additional 1-point profile (SMPG) on the three days immediately before Visits 2, 14, 18 and 28 in the main trial and before Visits 37 and 44 in the extension trial.

The plasma glucose levels were measured and recorded in the diary at the following time points, always starting with the measurement before breakfast on Day 1 (three days before site visit). The 9-point profile (SMPG) included the measurement before breakfast on Day 3. Each plasma glucose measurement for the 9-point profile (SMPG) included date, time point, actual clock time and plasma glucose value.

The timing of SMPG measurements is summarised Table 9–6.

Table 9–6 9-point profile (SMPG) (√) with additional 1-point profiles (SMPG) (X)

Time point	Day 1	Day 2	Day 3
Before breakfast	X	√	√
90 min. after the start of breakfast		√	
Before lunch		√	
90 min. after the start of lunch		√	
Before main evening meal		√	
90 min. after the start of the evening meal		√	
Before bedtime		√	
At 04:00 am		√	

Patient Reported Outcome Questionnaires

Patient Reported Outcomes (PROs) were to be assessed by a PRO battery consisting of 4 questionnaires only during the main trial

- Diabetes Medication Satisfaction Questionnaire (DiabMedSat)
- Diabetes Productivity Measure (DPM)
- Treatment Related Impact Measure – Diabetes (TRIM-D)
- Short-Form 36 Health Survey, Version 2 (SF-36, v2)

Hypoglycaemia Questionnaire

A hypoglycaemia questionnaire was to be used at all visits, but only if a subject reported to have experienced symptoms of one or more hypoglycaemic episodes (symptomatic or asymptomatic) since last visit or phone contact. The investigator or a trial coordinator completed the questionnaire in an interview.

9.5.1.3 Safety Assessments

Adverse Events

Adverse Events (AEs) were recorded by the subjects in the diaries at each visit and were to be transcribed from the diaries to the eCRF. Any clinically significant worsening since baseline of a previous finding was reported as an AE.

Adverse Event Definitions:

An AE is defined as any unfavourable and unintended sign, symptom or disease temporally associated with the use of a medicinal product, whether or not considered related to the medicinal product. This included events from the first trial related activity after the subject had signed the IC.

The following were not recorded as AEs:

- Pre-planned procedures unless the condition for which the procedure was planned has worsened from the first trial related activity at Visit 30 after the subject has signed the informed consent.
- Pre-existing conditions found as a result of screening procedures. These were recorded as medical history/concomitant illness.

An AE can also be a clinical laboratory abnormality regarded as clinically significant i.e. an abnormality that suggests a disease and/or organ toxicity and was of a severity which requires active management (i.e. change of dose, discontinuation of trial product, more frequent follow-up or diagnostic investigation).

A worsening in any concomitant illness present at Visit 1 in trial NN5401-3590 was to be recorded as an AE. Pre-existing conditions already reported as AEs or concomitant illnesses in trial NN5401-3590 were not to be recorded as AEs in NN5401-3726.

Serious adverse event (SAE)

A SAE is an experience that at any dose results in any of the following:

- Death
- A life-threatening* experience
- In-subject hospitalisation** or prolongation of existing hospitalisation
- A persistent or significant disability/incapacity***
- A congenital anomaly/birth defect

- Important medical events**** that may not result in death, be life-threatening*, or require hospitalisation** may be considered an SAE when, based upon appropriate medical judgement, they may jeopardise the subject and may require medical or surgical intervention to prevent one of the outcomes listed in this definition.

  * The term "life-threatening" in the definition of SAE refers to an event in which the subject was at risk of death at the time of the event. It does not refer to an event which hypothetically might have caused death if it was more severe.

  ** The term "hospitalisation" describes a period of at least 24 hours. Over-night stay for observation, treatment at emergency room or treatment on an out-subject-basis does not constitute a hospitalisation. However, medical judgement must always be exercised and when in doubt the case should be considered serious.

  Hospitalisations for administrative, trial related and social purposes do not constitute hospitalisations as defined by the seriousness criteria for SAEs and should therefore not be reported as such. Likewise, hospital admissions for surgical procedures planned prior to trial inclusion are not considered adverse events.

  *** The term "disability/incapacity" means that following the event the subject or clinical investigation subject has significant, persistent or permanent change, impairment, damage or disruption in his body function or structure, physical activity and/or quality of life.

  **** The term "important medical events" means events which may jeopardise the subject or require intervention to prevent a seriousness criterion. It can be AEs which suggest a significant hazard or puts the subject or clinical investigation subject at risk, such as drug-interactions, contra-indications or precautions, occurrence of malignancies or development of drug dependency or drug abuse.

Non-serious adverse event

A non-serious AE is any AE which does not fulfil the definition of a SAE.

Medical Events of Special Interest

Medical events of special interest (MESI) were used as a tool to collect additional information on special adverse events. Events such as medication errors (e.g., wrong drug administration or wrong route of administration) and suspected transmission of an infectious agent via a trial product were always considered as MESI.

MESI were always to be reported to the department responsible for global product safety using both the AE form and the safety information form, irrespective of seriousness. MESI were to be reported within the same timelines as for SAEs. In the event that the investigator chose not to report the

above specified AEs as MESI, there could be discrepancies between the protocol-defined MESI and the actually reported MESI.

In addition to the above, the following were also considered to be medical events of special interest for this trial: severe hypoglycaemia (see below for definition), immunogenicity, neoplasms and cardiovascular events.

- Severe Hypoglycaemia
  Any episode of hypoglycaemia, which fulfils the American Diabetes Association (ADA) definition for severe hypoglycaemia, i.e. an episode requiring assistance of another person to actively administer carbohydrate, glucagons, or other resuscitative actions, was to be reported always to the department responsible for global product safety on an AE form and safety information form irrespective of seriousness.
- Cardiovascular Events
  Cardiovascular events which were suspected as related to Acute Coronary Syndrome (ACS), stroke or cardiovascular death were to be reported always to the department responsible for global product safety on an AE form and safety information form irrespective of seriousness. Please see Appendix D of Protocol (Appendix 16.1.1) for definitions of cardiovascular events.
- Neoplasms
  All types of neoplasm events, i.e. benign, malignant and unspecified (including cysts and polyps) were always reported to the department responsible for global product safety on an adverse event form and safety information form irrespective of seriousness. Medical history and important follow-up data included information about malignancy versus non-malignancy, preferably histologically verified.
- Immunogenicity
  Events which in the opinion of the investigator were causally related to immune mechanisms to trial product, for example acute immunoglobulin E (IgE) mediated reactions (clinical signs may include anaphylactic reactions, angiooedema and urticaria) and delayed type hypersensitivity (clinical signs may include various types of skin rashes), were always reported to the department responsible for global product safety on an adverse event form and safety information form irrespectively of seriousness.

For further information regarding MESIs, see Appendix D to the protocol (Appendix 16.1.1).

Severity:

- Mild – No or transient symptoms, no interference with the subject's daily activities.
- Moderate - Marked symptoms, moderate interference with the subject's daily activities.
- Severe - Considerable interference with the subject's daily activities, unacceptable.

Relation to trial product:

- Probable: good reasons and sufficient documentation to assume a causal relationship

- Possible: a causal relationship is conceivable and cannot be dismissed
- Unlikely: the event is most likely related to an aetiology other than the trial product

* IDegAsp, IGlar, and NPH insulin must be included in the relationship assessment.

Relation to device:

Any device related issues were to be reported by the investigator to Novo Nordisk A/S.

Technical complaints are reported as part Novo Nordisk's continuous aggregated surveillance of device complaints. The technical complaint data are entered into the Novo Nordisk complaint handling system (COCPIT), whereafter they are investigated further. The results of the investigations are reported to the Novo Nordisk department responsible for safety surveillance on an ongoing basis. All adverse events related to technical complaints are reported in the clinical trial report, Section 12.2.2.3.

Outcome:

- Recovered - Fully recovered, or by medical or surgical treatment the condition has returned to the level observed at the first trial related activity at Visit 30 after the subject signed the informed consent.
- Recovering - The condition is improving and the subject is expected to recover from the event. This term should only be used when the subject has completed the trial.
- Recovered with sequelae - As a result of the AE, the subject suffered persistent and significant disability/incapacity (e.g. became blind, deaf, paralysed). Any AE recovered with sequelae should be rated as an SAE.
- Not recovered.
- Death.
- Unknown - This term should only be used in cases where the subject is lost to follow-up.

Reporting of Adverse Events

All adverse events either observed by the investigator or reported spontaneously by the subjects were recorded by the investigator and evaluated.

At each contact with the trial site (visit or telephone, excluding safety visits, where the subject was not seeing the investigator or his staff), the subject was to be asked about AEs.

The subject was asked about AEs (mentioned as 'Medical problems' in the diary) in the following way: "Have you experienced any medical problems since the last contact?"

All AEs either observed by the investigator or reported spontaneously by the subjects were recorded by the investigator and evaluated. All AEs were to be recorded by the investigator on the AE form in the electronic data capture (EDC) application.

The investigator was required to report SAEs to Novo Nordisk A/S within 24h. The investigator was to complete the AE form in the EDC application and the safety information form on the paper CRFs and forward to Novo Nordisk A/S within five calendar days.

Coding of Adverse Events

All the serious adverse events were coded using the most recent version of Medical Dictionary for Regulatory Activities (MedDRA) coding (version 14.0). Non-serious adverse events were coded locally by Novo Nordisk A/S personnel or a CRO using the semi-automated Thesaurus Management System (TMS). Serious adverse events were coded by medically qualified staff at International Product Safety, Novo Nordisk A/S. All adverse events were presented based on System Organ Class and Preferred Terms.

Treatment and Follow-up of Adverse Events

Any adverse events that occurred during the trial were treated by established standards of care. All serious adverse events and any non-serious adverse events that were severe or probably or possibly related to the trial product were followed up until the subject recovered, stabilised, recovered with sequelae, or died. However, cases of chronic conditions were closed with an outcome of "recovering" or "not recovered".

All other non-serious AEs were followed until the outcome of the event is "recovering" (for chronic conditions), or "recovered" or until the end of the post-treatment follow-up stated in the protocol, whichever comes first, and until all queries related to these AEs were resolved.

The investigator ensured that the worst case severity and seriousness was kept consistent.

Queries or follow-up requests from Novo Nordisk A/S were responded to within 14 calendar days, unless otherwise specified. The investigator recorded follow-up information on non-serious adverse events by updating the adverse event form in the eCRF.

The investigator forwarded follow-up information on serious adverse events within 5 calendar days of obtaining the information. This was done by updating the adverse event form in the eCRF and/or completing the safety information form on paper CRF and forwarding these to Novo Nordisk A/S.

All SAEs were followed until the outcome of the event was recovered, recovered with sequelae or fatal and until all queries were resolved. For cases of chronic conditions and cancer or if the subject died from another event follow-up until the outcome categories were "recovered", "recovered with sequelae" or "fatal" was not required, as these cases were closed with an outcome of "recovering" or "not recovered".

After access to update the AE form in the eCRF was removed the investigator recorded any SAE follow-up information, if required, on the paper CRFs provided at study closure.

Hypoglycaemic Episodes

Hypoglycaemic episodes were defined and classified according to the following:

ADA Hypoglycaemic Episode Definition- Classification[23]

ADA definitions and classification of hypoglycaemia were used for the statistical analyses. According to the ADA, the definition of a hypoglycaemic episode (refer to Figure 9–2) is categorised as:

Severe hypoglycaemia: an episode requiring assistance of another person to actively administer carbohydrate, glucagons, or other resuscitative actions

Documented symptomatic hypoglycaemia: an episode during which typical symptoms of hypoglycaemia are accompanied by a measured plasma glucose concentration ≤ 3.9 mmol/l (70 mg/dl).

Asymptomatic hypoglycaemia: an episode not accompanied by typical symptoms of hypoglycaemia, but with a measured plasma glucose concentration ≤ 3.9 mmol/l (70 mg/dl).

Probable symptomatic hypoglycaemia: an episode during which symptoms of hypoglycaemia are not accompanied by a plasma glucose determination (but that was presumably caused by a plasma glucose concentration ≤ 3.9 mmol/l (70 mg/dl).

Relative hypoglycaemia: an episode during which the person with diabetes reports any of the typical symptoms of hypoglycaemia, and interprets those as indicative of hypoglycaemia, but with a measured plasma glucose concentration > 3.9 mmol/l (70 mg/dl).

Unclassifiable: In case a hypoglycaemic episode could not be allocated into any of the above groups it is listed as unclassifiable.

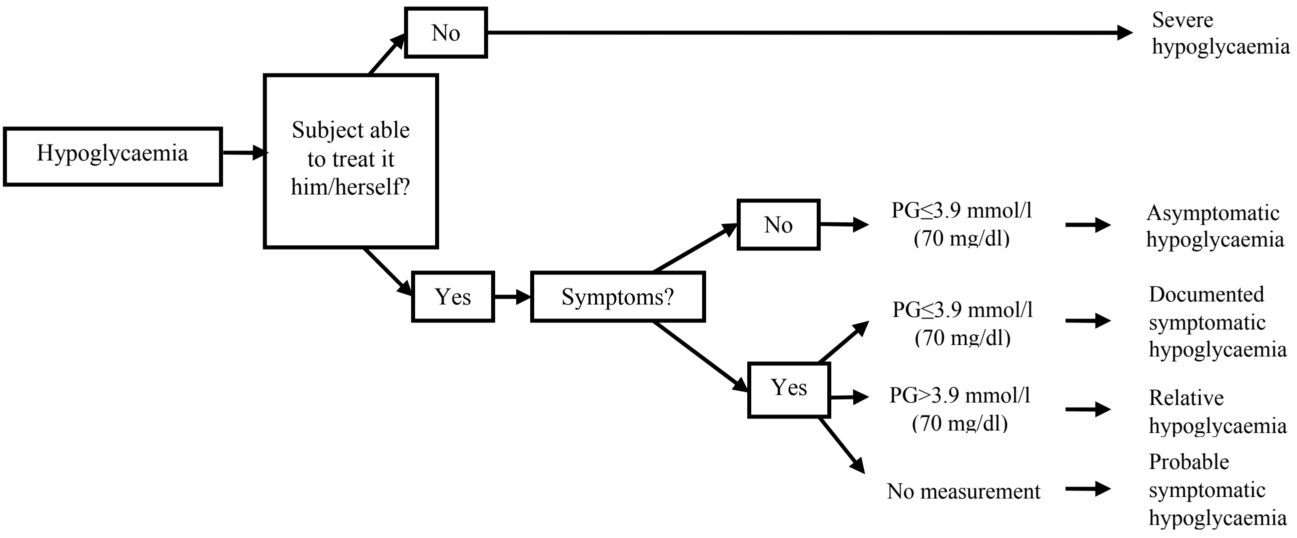

PG = plasma glucose

Figure 9–2 Classification of hypoglycaemia according to ADA

Additional Definition of Confirmed Hypoglycaemic Episodes

The pool of severe and minor hypoglycaemic episodes is referred to as confirmed hypoglycaemic episodes (See also 9.8.2). In normal physiology hypoglycaemia symptoms occur at a blood glucose level of approximately < 2.8 mmol/l (50 mg/dl) / plasma glucose level glucose < 3.1 mmol/l (56 mg/dl). Therefore Novo Nordisk A/S used this cut-off value to define minor hypoglycaemia. Minor hypoglycaemic episode is defined as:

- An episode with symptoms consistent with hypoglycaemia with confirmation by plasma glucose < 3.1 mmol/l (56 mg/dl) or full blood glucose < 2.8 mmol/l (50 mg/dl) and which is handled by the subject himself/herself
- Or any asymptomatic plasma glucose value < 3.1 mmol/l (56 mg/dl) or full blood glucose value < 2.8 mmol/l (50 mg/dl)

Hypoglycaemic episodes were only recorded as adverse events if they fulfilled the definition of an SAE or if reported as a MESI case of severe hypoglycaemia.

Antibodies

During both main and extension trial serum samples were mixed with radio-labelled IDeg, IAsp, IGlar or human insulin and incubated overnight. Antigen/antibody complexes were precipitated

using polyethyleneglycol and bound radioactivity in the precipitate was used to express antibody binding (% bound/total radioactivity: %B/T).

Insulin dose

During both main and extension trials (starting at the Visit 2 and Visit 30), subjects were instructed to report the date, time point and insulin dose in the diary on preferably three consecutive days before each visit. The days where the insulin doses were recorded were to be the same days as when the SMPGs were recorded. The recommended insulin doses were calculated automatically in the eCRF based on recommendations from the Insulin Titration Guideline.

Physical examination

Physical examination was carried out at Visits 1 and 28 in the main trial and at Visit 44 in the extension trial and included:

- Head, ears, eyes, nose, throat, neck
- Respiratory system
- Cardiovascular system
- Gastrointestinal system including mouth
- Musculoskeletal system
- Central and peripheral nervous system
- Skin

The physical examination results from trial NN5401-3590 end-of treatment visit (Visit 28) were transferred to extension trial screening visit (Visit 30) and the assessment was only performed at extension trial screening visit (Visit 30) if not done at trial NN5401-3590 end-of treatment visit (Visit 28).

If any clinically significant worsening from trial NN5401-3590 baseline (Visit 1) occurred, the investigator was to comment in the subject notes and, report this as an AE in trial NN5401-3590.

Any clinically significant worsening from baseline in the main and extension trial was reported as an AE.

Vital signs

Diastolic and systolic blood pressures and pulse were measured at Visits 1 and 28 in the main trial and at Visit 44 in the extension trial. Diastolic blood pressure, systolic blood pressure and pulse were assessed while the subject was sitting. Measurements were performed after resting for five minutes. Any clinically significant worsening from baseline was to be reported as an AE.

Body weight

Body weight was measured at Visits 1 and 28 in the main trial and at Visit 30, Visit 37, and Visit 44 in the extension trial. Body weight was measured in kilograms (kg) or pounds (lb) without coat and shoes and wearing only light clothing. Body weight was recorded to one decimal place. The subjects were to attend these visits fasting (i.e., only intake of water since midnight was allowed).

Eye examination

Eye examination was carried out at Visits 1 and 28 in the main trial and at Visit 30 and Visit 44 in the extension trial. Fundoscopy/fundusphotography was performed by the investigator, a local ophthalmologist or an optometrist according to local practice. Result of the fundoscopy/fundusphotography was interpreted locally by the investigator in relation to the trial. Fundoscopy/fundusphotography performed within 8 weeks before Visit 2 and 2 weeks before Visit 28 in the main trial or within three weeks before end-of-treatment visit (Visit 44) was acceptable. The results of the examination were to be signed and dated by the investigator and evaluated following the categories:

- “Normal”
- “Abnormal, not clinically significant”
- “Abnormal, clinically significant”

Any clinically significant worsening of the fundoscopy/fundusphotography result from baseline in both main and extension trial was to be reported as an AE.

Electrocardiogram (ECG) – 12 lead

A 12-lead ECG was performed at Visits 1 and 28 in the main trial and at Visit 44 in the extension trial. It was interpreted locally by the investigator in relation to the trial according to the following categories:

- “Normal”
- “Abnormal, not clinically significant”
- “Abnormal, clinically significant”

12-lead ECG was required both in accordance with trial NN5401-3590 end-of-treatment visit (Visit 28) and extension trial screening visit (Visit 30). The results from trial NN5401-3590 end-of-treatment visit (Visit 28) were transferred to extension trial screening visit (Visit 30) and the assessment was only performed at extension trial screening visit (Visit 30) if not done at trial NN5401-3590 end-of-treatment visit (Visit 28).

Any clinically significant worsening from trial NN5401-3590 baseline (Visit 1) was commented in the subject notes and reported as an AE by the investigator in trial NN5401-3590. Any clinically significant worsening of the ECG result from baseline (Visit 30) was to be reported as an AE.

Clinical Laboratory Tests

For laboratory analysis of safety and efficacy parameters a total of approximately 130 mL blood was drawn during the main trial and approximately 80 ml blood was drawn during the extension trial for each subject. The laboratory analyses were performed by a central laboratory unless otherwise specified. Descriptions of assay methods, laboratory supplies and procedures for obtaining samples, handling and storage of samples are provided in Appendix 16.1.10.

Laboratory samples (including HbA_{1c}, FPG and pregnancy test) could be drawn on a day other than the day of the actual visit as long as it was within the visit window (e.g., if the subject was not fasting). For measurement of insulin antibodies, serum samples were mixed with radio-labelled IDeg, IAsp, IGlar, or human insulin and incubated overnight. Antigen/Antibody complexes were precipitated using polyethyleneglycol and bound radioactivity in the precipitate was used to express antibody binding (% bound/ total radioactivity: % B/T).

The following clinical laboratory tests were performed during the trial:

- Haematology and biochemistry (erythrocytes, haematocrit, haemoglobin, leucocytes, thrombocytes, and differential count)
- Insulin antibodies (determination of IDeg-specific, IAsp-specific and IGlar-specific antibodies, and antibodies cross-reacting to human insulin)
- Biochemistry (creatinine, total protein, alanine amino transferase /serum glutamic pyruvic transaminase [ALAT/SGPT], aspartate amino transferase/serum glutamic oxaloacetic transaminase [ASAT/SGOT], alkaline phosphatase [AP], sodium, potassium, albumin, and total bilirubin)
- Lipid profile (total cholesterol, high density lipoproteins [HDL cholesterol], low density lipoproteins [LDL cholesterol], and triglycerides [TG])
- Cardiovascular risk markers (high sensitive C-reactive protein (hsCRP), and B-type natriuretic peptide (NT-proBNP)
- Urinary albumin-to-creatinine ratio assessed in spot urine
- Urine by sticks (tests for blood, protein, ketones)

For some of the samples drawn during the trial, it was required for the sensitivity of the analysis that the subject was fasting.

Samples were coded in order to keep subject identity anonymous.

Laboratory results were sent by the central laboratory to the investigator on an ongoing basis. Data from the analysis were included in the CTR and the clinical trial database. The antibody data were also included in an analytical report. All laboratory printouts were dated and signed by the investigator on the day of evaluation. If a result was outside the normal range, the investigator

judged whether the abnormality was clinically significant. Any clinically significant was reported as an AE.

All samples were destroyed on an ongoing basis after analysis except for insulin antibody samples which were to be stored for possible future analysis.

Pregnancy

Subjects were to notify the investigator immediately if they became pregnant. The investigator was to report any pregnancy reported during the trial to Novo Nordisk A/S, except for pregnancies occurring in the screening period. On enrolment, subjects were to give consent to the investigator reporting any pregnancy during the trial to Novo Nordisk A/S and to provide information about pregnancy, delivery and the health of the infant until age one month.

The investigator was to report information on pregnancy and follow-up within 14 calendar days of obtaining the information. If the pregnancy resulted in an abnormal outcome, such as congenital abnormalities, foetal death, spontaneous abortion, or serious AEs in the neonate, this information was to be forwarded to the department responsible for global product safety within 72 hours from receipt.

For pregnancies in partners of trial subjects, in case of an AE (with a causal relationship evaluated as possible or probable by the investigator) in the foetus, newborn infant(s) or infant(s)/toddler(s) of a trial subject's partner, who was potentially exposed to the trial product via the trial subject, the pregnancy and the AE was to be reported on the same forms as for a subject in the trial.

9.5.1.4 Assessments of Demographic and other Baseline Characteristics

The following baseline characteristics were to be recorded at Visit 1 in the main trial:

- Demography (date of birth, gender, race, ethnicity)
- Diabetes data (history, complications and current therapy)
- Medical history, concomitant illnesses (also recorded at Visit 30 in the extension trial)

Height

At screening visit (Visit 1), height (without shoes) was to be measured in inches or metres and recorded with two decimal places.

Body mass index

BMI was calculated using the eCRF. BMI calculation: BMI kg/m^2= Body weight (kg)/(Height (m) x Height (m)) (kg/m^2 = [lb/in^2 x 703]).

Body weight

Body weight was to be measured in kilogram (kg) or pounds (lb) without coat and shoes. Body weight was to be recorded to 1 decimal place. The subjects were to attend these visits fasting (i.e., only intake of water since midnight was allowed) except for screening visit (Visit 1).

Demography, diagnosis of diabetes, height and medical history were transferred from trial NN5401-3590 screening visit (Visit 1) to extension trial screening visit (Visit 30). Baseline was defined at the time of randomisation in the main trial.

Concomitant medication recorded during trial NN5401-3590 that was still taken at the start of the extension trial was transcribed by the investigator to a concomitant medication form for the extension trial at the screening visit.

9.5.2 Appropriateness of Measurements

Both efficacy and safety assessments were standard for this type of trial.

9.5.3 Primary Efficacy Variable(s)

Since the primary objective of the present extension trial was to investigate the long-term safety and tolerability of IDegAsp, safety variables including adverse events, hypoglycaemic episodes, clinical evaluations, central laboratory assessments, body weight and insulin dose were the primary endpoints. There were no primary efficacy variables in this extension trial.

9.6 Data Quality Assurance

The trial was conducted in accordance with Declaration of Helsinki[1] and ICH Good Clinical Practice.[2]

The trial was monitored by the sponsor by means of on-site visits, telephone calls, and regular inspection of the case report forms (CRFs) with sufficient frequency to verify the following: Subject enrolment; compliance with the protocol; the completeness and accuracy of data entered in the CRFs by verification against original source documents; compliance in the use of trial product; drug accountability; and recording of adverse events.

9.7 Statistical Methods and Determination of Sample Size

9.7.1 General Considerations

The statistical section described the planned statistical analyses as described in the trial protocol (Appendix 16.1.1). Any changes from the planned analyses were described in Section 9.8.2.

9.7.2 Endpoints in Relation to the Objectives and Hypotheses

The primary objective was to investigate the long-term safety and tolerability of IDegAsp. This was done by comparing IDegAsp to IGlar after 52 weeks of treatment in terms of safety assessments. The secondary objective was to compare efficacy between IDegAsp and IGlar after 52 weeks of treatment.

Baseline was defined as the time of randomisation in the main trial. Unless otherwise specified, missing values including intermittent missing values and values missing in both main trial and extension trial were imputed using the Last Observation Carried Forward (LOCF) method as recommended for its transparency in the FDA guidance.[24] In the following section "end of trial" means a subject's last end of trial visit excluding the follow-up visit in the extension trial.

All endpoints were summarised descriptively at each scheduled visit by treatment and in total using observed data. After 52 weeks of treatment, descriptive statistics were presented based on both observed and LOCF imputed data.

LOCF imputed data were used as the basis for plotting data if not otherwise specified. For survival endpoints, Kaplan-Meier plots were presented by treatment.

Only endpoints derived after 52 weeks of treatment were analysed statistically unless otherwise stated. Except for possible exploratory analyses, the statistical models were specified to always include antidiabetic therapy at screening, sex and region (Europe [Spain, Austria, Poland, Turkey, Russia], Asia [Republic of Korea, India], and North America [United States]) as fixed factors, and age and baseline value as covariates. Antidiabetic therapy at screening was a factor with two levels according to the categories in the main trial (metformin + sulfonylurea [SU] or glinides and metformin + DPP-4 inhibitor ± SU or glinides ± α-glucosidase-inhibitors [acarbose]).

Endpoints that were analysed untransformed and endpoints that were not formally analysed were summarised by the arithmetic mean, standard deviation (SD), median, and minimum and maximum values. Endpoints that were analysed log-transformed were summarised by the geometric mean, CV, median, minimum and maximum values.

Presentation of results from statistical analyses included the estimated mean treatment effects (LSMeans) for absolute values and change from baseline if applicable. Estimated mean treatment differences (or ratios) were presented together with two-sided 95% confidence intervals for all endpoints analysed statistically. No p-values were presented as all analyses were considered exploratory.

Safety endpoints were summarised and analysed using the Safety Analysis Set. Efficacy endpoints were summarised and analysed using the Full Analysis Set (FAS). The analysis of secondary

endpoint, HbA_{1c}, was repeated on the per protocol (PP) analysis set in accordance with the Committee for Proprietary Medicinal Products (CPMP) Points to Consider (CPMP/EWP/482/99).[25]

Analyses were repeated for serious treatment emergent AEs, number of severe and minor treatment emergent hypoglycaemic episodes, antibodies, central laboratory parameters (ALAT/SGPT, ASAT/SGOT) and HbA_{1c} using the Extension Trial Set (ETS) to assess the stability of key results.

The impact of protocol deviations and outliers were to be investigated further in sensitivity analyses if deemed relevant.

9.7.3 Definition of Analysis Sets

The following analysis sets were defined in accordance with the ICH-E9 guidance as stated in the main trial (NN5401-3590) protocol.[26]

- Full analysis set (FAS): included all randomised subjects. In exceptional cases subjects from the FAS could be eliminated. In such cases the elimination was to be justified and documented. The statistical evaluation of the FAS was to follow the intention-to-treat (ITT) principle and subjects were to contribute to the evaluation “as randomised”.

- Per Protocol (PP) analysis set: included subjects without any major protocol violations that may have affected the primary endpoint. Moreover, subjects must have been exposed to the investigational product or its comparator for more than 12 weeks and must have had a valid assessment necessary for deriving the primary endpoint. Subjects in the PP set were to contribute to the evaluation “as treated”.

- Safety Analysis Set (SAS): included all subjects receiving at least one dose of the investigational product or its comparator. Subjects in the safety set were to contribute to the evaluation “as treated”.

- Extension Trial Set (ETS): included subjects receiving at least one dose of the investigational product or its comparator in the extension trial.

The analysis sets (FAS, PP and SAS) were defined in the main trial and the assignment of subjects to these analysis sets in the extension trial was following the definition in the main trial.

Randomised subjects who were lost to follow-up and where no exposure information of the investigational product or its comparator was available after randomisation were to be handled as unexposed.

Before data were released for statistical analysis, a blinded review of all data was to take place to identify protocol deviations that may potentially have affected the results. This review was to be performed without revealing the trial product to which the subjects were assigned. The blinding of

the trial products was to be maintained for everyone involved in defining the analysis sets until data were released for statistical analysis. Furthermore, extreme values and outliers were to be identified by the statistician during programming and data review according to ICH-E9, using a fake randomisation.

The decision to exclude any subject or observation from the statistical analysis was the joint responsibility of the study group. The subjects or observations to be excluded and the reason for their exclusion were to be documented and signed by all parties, prior to release of the clinical trial database. The documentation was to be stored together with the remaining trial documentation.

9.7.4 Statistical Analyses

9.7.4.1 Primary Endpoints

The following primary safety endpoints were assessed:

- Adverse events
  - Number of events by medical event of special interest (MESI), System Organ Class and Preferred Term, seriousness, severity, relation to trial drug and device, and withdrawal due to AEs and outcome
- Hypoglycaemic episodes
  - Number of episodes by severity (classified according to the American Diabetes Association (ADA) classification and the additional definition for minor episodes)
  - Number of nocturnal episodes by severity (classified according to the ADA classification and the additional definition for minor episodes)
- Clinical evaluations
  - Physical examination
  - Fundoscopy/fundusphotography
  - 12-lead electrocardiogram (ECG)
  - Vital signs
- Central laboratory
  - Haematology (haemoglobin, leucocytes, thrombocytes, haematocrit, differential counts and erythrocytes)
  - Biochemistry (creatinine, total protein, alanine aminotransferase (ALAT), aspartate aminotransferase (ASAT), alkaline phosphatase (AP), sodium, potassium, albumin, and total bilirubin)
  - Lipid profile (low density lipoproteins (LDL), high density lipoproteins (HDL), triglyceride (TG) and total cholesterol)
  - Cardiovascular risk markers (high sensitive C-reactive protein (hsCRP), and B-type natriuretic peptide (NT-proBNP)
  - Urinary albumin-to-creatinine ratio assessed in spot urine
  - Urine by sticks (central tests for blood, protein and ketones)

  - Insulin antibodies (IDeg-, IGlar- and IAsp- specific antibodies in the corresponding treatment arms, human insulin cross-reacting antibodies in both arms)
- Body weight
- Insulin dose and time of administration

The timing of each endpoint was defined in the trial flow chart in both the main trial and this extension trial protocol.

Adverse events

Adverse Events were to be coded using the most recent version of Medical Dictionary for Regulatory Activities (MedDRA) coding. All adverse events were presented based on System Organ Class and Preferred Terms.

When reporting the trial results the EAC evaluation was employed. All discrepancies between EAC and the investigators' classification of the cardiovascular events were listed by event.

A Treatment Emergent Adverse Event (TEAE) was defined as an event that had onset date on or after the first day of exposure to randomised treatment in the main trial and no later than seven days after the last day of randomised treatment. All AEs occurring in the follow-up period of the main trial for subjects continuing in the extension trial were considered treatment emergent independently of the length of the follow-up period.

TEAEs were summarised descriptively, whereas AEs not defined as treatment emergent were presented in listings. The summaries of TEAEs were made displaying the number of subjects with at least one event, the percentage of subjects with at least one event, the number of events and the event rate per 100 years. These summaries were done by seriousness, severity, relation to insulin treatment, relation to device, withdrawal due to AEs and outcome.

Furthermore summary tables based on System Organ Class and Preferred Terms were made for:

- All TEAEs
- Serious TEAEs
- Possibly or probably related TEAEs
- Severe TEAEs
- TEAEs with Preferred Term that are experienced by at least 5% of the subjects in any treatment arm or by at least 5% of all subjects.

Hypoglycaemic episodes

Hypoglycaemic episodes were recorded by subjects in their trial diaries throughout the trial. The information collected was used to classify an episode according to both the ADA definition (severe, documented symptomatic, asymptomatic, probable symptomatic and relative) and the additional classification for minor hypoglycaemic episodes.

A hypoglycaemic episode was defined as treatment emergent if the onset of the episode was on or after the first day of exposure to randomised treatment and no later than seven days after the last day of randomised treatment. All episodes occurring in the follow-up period of the main trial for subjects continuing in the extension trial were considered treatment emergent independent of the length of the follow-up period.

The summaries of treatment emergent hypoglycaemic episodes were the following:

- the number of subjects with at least one episode
- the percentage of subjects with at least one episode
- the number of episode and the episode rate per 100 years
- separate summaries by severity:
  - all episodes
  - nocturnal episodes using both the ADA and the additional minor category (refer to Section 9.5.1.3)

The nocturnal period was considered as the period between 00:01 and 05:59 a.m. (both included). A hypoglycaemic episode that had time of onset during this period was to be considered nocturnal.

The number of hypoglycaemic episodes was analysed using a negative binomial regression model with a log-link function and the logarithm of the time period in which a hypoglycaemic episode was considered treatment emergent as offset. The model included treatment, antidiabetic therapy at screening, sex and region as fixed factors, and age as covariate. To the extent where data allow, separate analyses were performed for severe episodes, and severe or documented symptomatic episodes considering all episodes and nocturnal episodes separately.

Physical examination

Physical examination included:

- Head, ears, eyes, nose, throat, neck
- Respiratory system
- Cardiovascular system
- Gastrointestinal system including mouth
- Musculoskeletal system
- Central and peripheral nervous system
- Skin

The physical examination measurements and their change from baseline were summarised descriptively.

Vital Signs

Vital signs included diastolic blood pressure, systolic blood pressure and pulse. The measurements and their change from baseline were summarised descriptively.

Laboratory safety parameters

The following laboratory assessments were performed:

- Haematology (haemoglobin, leucocytes, thrombocytes, erythrocytes, haematocrit, differential counts)
- Biochemistry (creatinine, total protein, ALAT/SGPT, ASAT/SGOT, AP, sodium, potassium,
- albumin, total bilirubin)
- Lipid profile (LDL, HDL, TG and total cholesterol)
- Cardiovascular risk markers (high sensitive C-reactive protein (hsCRP), and B-type natriuretic peptide (NT-proBNP)
- Urinary albumin-to-creatinine ratio assessed in spot urine
- Urine by sticks (tests for blood, protein and ketones)
- Insulin antibodies

Individual laboratory values were compared to their relevant reference range (when existing) and flagged as being below or above the range. Change from baseline was summarised descriptively.

Change from baseline (baseline in the main trial) in hsCRP, NT-proBNP, and lipid endpoints was analysed separately using an Analysis of Variance (ANOVA) method with treatment, antidiabetic therapy at screening, sex and region as fixed factors, and age and baseline value as covariates.

IDeg specific antibodies, IGlar specific antibodies, IAsp specific antibodies and cross-reacting antibodies and the correlation to other relevant variables such as, for example, insulin dose and HbA_{1c} were illustrated using descriptive statistics and graphs.

Fundoscopy / fundusphotography

Fundoscopy and fundusphotography findings were summarised descriptively including summaries of the change from baseline.

ECG

ECG 12-lead findings were summarised descriptively including summaries of the change from baseline.

Body weight

Body weight was assessed at the trial site. Change from baseline in body weight was analysed using an ANOVA method with treatment, antidiabetic therapy at screening, sex and region as fixed factors, and age and baseline body weight as covariates.

Insulin dose and time of administration

Prescribed and actual insulin dose were recorded together with time of administration. The insulin dose was summarised descriptively according to regimen as dose in units, units/kg and units/kg per week. The summaries also included information on time of insulin administration in order to document the time of the day patients inject their insulin and the degree to which patients shift injection times during the trial.

9.7.4.2 Secondary Endpoints

Efficacy was addressed in terms of the following list of assessments from which endpoints were calculated, analysed and presented:

- HbA_{1c}
- FPG
- 9-point profile (SMPG)
- SMPG for dose adjustments

HbA_{1c}

The endpoints from the HbA_{1c} assessments were:

- Change from baseline in HbA_{1c} after 52 weeks of treatment
- Responder for HbA_{1c} (< 7.0% and ≤ 6.5%)
- Responder for HbA_{1c} at end of trial (< 7.0% and ≤ 6.5%) without hypoglycaemic episodes

Change from baseline in HbA_{1c} after 52 weeks of treatments

This endpoint was analysed using an ANOVA method with treatment, antidiabetic therapy at screening, sex and region as fixed factors, and age and baseline HbA_{1c} as covariates.

As sensitivity analyses, all observed HbA_{1c} measurements available post randomisation at scheduled measurement times were also analysed in a linear mixed model using an unstructured residual covariance matrix (if possible) as done in the main trial. Any marked difference concerning treatment differences between the different analyses methods was commented upon in the clinical trial report.

Change in HbA_{1c} from baseline was also analysed using a model with only treatment as fixed factor and baseline HbA_{1c} as covariate to assess the sensitivity of the results to inclusion/exclusion of fixed factors and covariates.

Responder endpoints

Two dichotomous endpoints (responder/non-responder) were defined based on whether a subject had meet the American Diabetes Association (ADA) HbA_{1c} target (HbA_{1c} < 7%) and the International Diabetes Federation (IDF) HbA_{1c} target (HbA_{1c} ≤ 6.5%).

Additional dichotomous endpoints were defined based on whether those treatment targets at end of trial were achieved without hypoglycaemic episodes in the last 12 weeks of treatment considering severe episodes only, and severe and minor episodes together. The endpoints were only defined for subjects that have been exposed for at least 12 weeks in the main trial.

The responder endpoints were analysed separately based on a logistic regression model using treatment, antidiabetic therapy at screening, sex and region as fixed factors, and age and baseline HbA_{1c} as covariates.

Fasting plasma glucose

Change from baseline in FPG after 52 weeks of treatment was analysed using an ANOVA method with treatment, antidiabetic therapy at screening, sex and region as fixed factors, and age and baseline FPG as covariates.

Self measured plasma glucose

Self-measured plasma glucose was measured in terms of the 9-point profile (SMPG) and glucose measurements used for insulin dose adjustments.

9-point profile (SMPG)

A 9-point profile (SMPG) included measurements before and 90 minutes after start of breakfast, lunch and main evening meal, measurements prior to bedtime and at 4 a.m., and one before breakfast the following day.

The endpoints from a 9-point profile (SMPG) were:

- Mean of the 9-point profile (SMPG)
- Fluctuation in 9-point profile (SMPG)
- Prandial PG increment
- Changes in nocturnal SMPG measurements

The mean of 9-point profile (SMPG) was defined as the area under the profile divided by the measurement time and was calculated using the trapezoidal method. The fluctuation in the 9-point profile (SMPG) was defined as

$$\frac{1}{T}\int_0^T \left| PG(t) - \overline{PG} \right| dt$$

where T, $PG(t)$ and, $\overline{PG}$ denotes the length of the profile, the PG value at time t and the mean of the profile, respectively.

Prandial PG increment for each meal was derived from the 9-point profile (SMPG) as the difference between PG values available 90 minutes after meal and before meal. Mean prandial PG increment over all meals was derived as the mean of all available meal increments.

Change in nocturnal PG was assessed by considering the differences between PG values available prior to bedtime, at 4 a.m. and the before breakfast value the following day: (4 a.m. PG value minus prior bedtime PG value), (before breakfast PG value minus prior bedtime PG value) and (before breakfast PG value minus 4 a.m. PG value).

A mixed effect model was fitted to the 9-point profile (SMPG) data. The model included treatment, time, interaction between treatment and time, antidiabetic therapy at screening, sex and region as fixed factors, age as covariate and subject as random effect. From the model the mean profile by treatment and relevant treatment differences were estimated and explored.

Mean and fluctuation in the 9-point profile (SMPG), prandial PG increment and nocturnal PG endpoints were analysed separately using an ANOVA method with treatment, antidiabetic therapy at screening, sex and region as fixed factors, and age and the relevant baseline value as covariates. Fluctuation in the 9-point profile (SMPG) was logarithm-transformed before analysed.

SMPG values used for dose adjustment

The SMPG values used for dose adjustment were collected and recorded by the subjects for three consecutive days prior to visits and included measurements before main meals (2-4 times daily). Dose adjustment of basal insulin was based on the subject’s before breakfast value, and this value was always obtained even if a subject did not eat breakfast. The titration algorithm, including information on adjustment of bolus insulin, was detailed in the Insulin Titration Guideline, Appendix C.

The endpoints from SMPG measurements obtained throughout the trial for dose adjustment were:

- Mean PG before meal
- Responder for PG titration target
- Time from randomisation (measured in weeks) to achieve titration target
- Within-subject variability as measured by CV%

The mean PG before meal was calculated at each visit using the available data and separately for each meal where data was available.

The mean of before breakfast PG values was analysed using an ANOVA method with treatment, antidiabetic therapy at screening, sex and region as fixed factors, and age and the corresponding mean PG at baseline as covariates.

From the mean before breakfast PG value a dichotomous endpoint (responder/non-responder) was derived showing if a subject has achieved the titration target at each visit.

Two survival endpoints were derived as the time from randomisation to the date a subject met the titration target for the first time, and the time to when a subject met the titration target and stayed on the target for the remaining treatment period.

The survival endpoints were analysed separately in a Cox proportional hazards model including treatment, antidiabetic therapy at screening, sex and region as fixed factors and age as covariate. Subjects that were lost for follow-up without meeting the target and subjects that never met the target during treatment were censored at the last day of treatment.

The logarithm transformed SMPG values available before breakfast were analysed as repeated measures in a linear mixed model with treatment, antidiabetic therapy at screening, sex and region as fixed factors and age as covariate and subject as random factor. The model assumed independent within- and between-subject errors with variances depending on treatment. Within-subject variability as measured by CV% for a treatment was calculated from the corresponding residual variance σ^2 as $CV\% = 100\sqrt{\left(\exp\left(\sigma^2\right) - 1\right)}$. The confidence interval for the CV ratio between treatments was calculated using the delta method.

9.7.5 Interim Analyses

No interim analysis was planned for this trial.

9.7.6 Sequential Safety Analysis/Safety Monitoring

Not applicable.

9.7.7 Exploratory Statistical Analyses for Pharmacogenetics and Biomarkers

Not applicable.

9.7.8 PK and PD Modelling

Not applicable.

9.7.9 Health Economics and/or Patient Reported Outcomes

Not applicable.

9.7.10 Determination of Sample Size

Sample size in this extension trial was determined by the number of subjects continuing from the main trial. At the first visit in the extension trial (Visit 30) subjects discontinued the regimen of NPH insulin received in the follow-up period of the main trial and restarted the same treatment

regimen as in the main trial (NN5401-3590) with either IDegAsp OD + metformin or IGlar OD + metformin.

It was assumed that 75% of the completers in the main trial would continue in the extension trial from which at least 85% were expected to complete the extension trial.

9.8 Changes in the Conduct of the Study or Planned Analyses

9.8.1 Protocol Amendments and General Procedural Deviations

Protocol Amendments

There were 5 substantial amendments to the protocol (see Table 9–7). None of the substantial amendments were considered to have any serious implications for the subjects involved in this trial, nor on the overall results of this trial. Substantial amendment 1 was implemented before trial initiation (see Table 9–7 for details). Substantial amendments 4 and 5 were implemented after trial initiation. Refer to the protocol amendments (Appendix 16.1.1), for further details.

Table 9–7 Substantial Protocol Amendments

Amendment Type	Amendment No.	Date	Countries affected	Content change
Substantial	1_US	09-Jul-2010	US	The wording was removed that described the requirement to distribute the package insert for trial products to subjects at dispensing visits.
Substantial	2	14-Jul-2010	Global	The time point of IDegAsp dosing was changed from dosing with breakfast to the possibility of dosing with breakfast or with the largest meal. However, some countries obtained approval of this amendment late in the trial.
Substantial	3_IN	09-Aug-2010	India	The wording was removed that described the requirement to distribute the package insert for trial products to subjects at dispensing visits.
Substantial	4	09-Feb-2011	Global	The wording describing the laboratory safety assessments was updated to include what laboratory safety assessments would be reported to the site.
Substantial	5_TR	11-Mar-2011	Turkey	New country-level local trial manager (LTM) assigned; coordinator site changed name and location and assigned new principal investigator (PI).

General Procedural Deviations

There were no important protocol deviations at country level and trial level (see Appendix 16.2.2). See Section 10.2 for important protocol deviations on trial site level and on subject level.

9.8.2 Changes to the Statistical Analysis Planned in the Protocol

No statistical analysis plan was created for this trial as the statistical analyses were specified in sufficient detail in the protocol. The text below refers to changes to the protocol. All changes were made before Data Base Lock.

All formal statistical analyses (including Statistical analysis on hypoglycaemic episodes, body weight and lipids) were based on the FAS. This was done to facilitate interpretability and ensure consistency within and between trials in the clinical development programme. Demographic and baseline characteristics of subjects who participated in the extension trial were analysed using the extension trial set.

The model fitted to the 9-point SMPG profiles also included the values from the 9-point SMPG profile at baseline as covariate as it was considered relevant to adjust for the values at baseline.

With regards to the statistical analyses of hypoglycaemic episodes, the text in the protocol stated that “to the extent where data allow, separate analyses will be performed for severe episodes, severe or documented symptomatic episodes considering all episodes and nocturnal episodes separately.” As documented in the minutes from the database lock, no analysis of severe episodes was carried out due to few numbers of events.

The term “confirmed hypoglycaemic episodes” was used in place of “severe and minor hypoglycaemic episodes”.

Only one survival endpoint was derived (time to meet the target for the first time) as the endpoint describing time until reaching and maintaining target was not considered to be clinically meaningful.

As stated in the protocol (Appendix 16.1.1) and Section 9.5.1.3, the further classification of pre-specified adverse events as medical events of special interest (MESI) was used as a tool to collect additional information on special adverse events. Summaries based on standard MedDRA query (SMQ) searches will be presented for injection site reactions, immunogenicity, neoplasms and medication errors. These summaries will then form the basis for the presentation of these events in Section 12.2.2 and Section 12.3.3.2. The Preferred Terms and/or SOCs included in the SMQ searches are listed in Appendix 16.2.7, Listing 16.2.7.4.

10 Trial Subjects

10.1 Disposition of Subjects

A total of 813 subjects were screened and 530 subjects were randomised in the main trial. All of the randomised subjects except for four were exposed to trial product; these four were therefore excluded from the safety analysis set. The 530 subjects were randomised 1:1 to IDegAsp or IGlar. In total, 79 subjects (14.9% of those randomised) were withdrawn from the trial after randomisation.

Out of the 451 subjects who completed the main trial, 38 subjects did not continue into the screening visit for the extension trial. Of these 38 subjects, 27 subjects were treated with IDegAsp and 11 were treated with IGlar. A total of 413 subjects were screened and enrolled in the extension trial (192 subjects in the IDegAsp group and 221 subjects in the IGlar group) and none of them were screening failures.

In total, 388 subjects completed the extension trial; 179 (67.3%) subjects in the IDegAsp group and 209 (79.2%) subjects in the IGlar group. In total 25 subjects (4.7% of all randomised subjects) were withdrawn from the extension trial. Similar numbers of exposed subjects withdrew during the extension trial in the IDegAsp (13 subjects) and IGlar (12 subjects) treatment groups. The overall withdrawal pattern (reasons for withdrawal, time of withdrawal) was comparable between the two treatment groups and a similar proportion of subjects completed the trial in the two treatment arms; see Table 10–1. In general, the subject withdrawals occurred throughout the trial period, with no apparent clustering of withdrawals at any specific time point during the trial; see EOT Figures 14.1.3 and 14.1.4.

The primary reason for withdrawal in both groups was in the category of ‘other’ reasons, i.e. withdrawal of consent (3 subjects), lost to follow-up (2 subjects), refusal of continuation of the trial due to personal reason (1 subject), unable to continue visits (1 subject), subject-initiated discontinuation (1 subject), uncontrolled diet due to depressive syndrome which caused frequent hypoglycaemia (1 subject), and 1 subject was withdrawn due to concomitant illness during the trial. Subject 812010 was withdrawn due to death (myocardial infarction with fatal outcome that occurred in the IDegAsp treatment group; Investigator/ Sponsor causality assessment: Unlikely). See Appendix 16.2.1, Listing 16.2.1.3 for details. A listing of all deaths is included in EOT Listing 14.3.2. Narratives for deaths are included in EOT Listing 14.3.3.

The number of withdrawals due to non-compliance was 5 (2 in the IDegAsp group and 3 in the IGlar group).

Six (6) subjects (3 in the IDegAsp group and 3 in the IGlar group) were withdrawn due to adverse events in the extension trial. See Appendix 16.2.1, Listing 16.2.1.3 for details.

In total, 3 subjects were withdrawn because they met one withdrawal criterion (2 in the IDegAsp group and 1 in the IGlar group). Of these, 1 subject (in the IDegAsp group) was withdrawn due to a major protocol deviation having influence on efficacy or safety data as judged by the investigator and 2 subjects (1 in the IDegAsp group and 1 in the IGlar group) withdrew due to lack of treatment effect according to the criteria specified in the protocol.

Subject disposition by country is presented in EOT Table 14.1.2. Information about individual subject disposition is shown in Appendix 16.2.4, Listing 16.2.4.1 and subject disposition by week is presented in EOT Table 14.1.5. Reasons for withdrawal are summarised in EOT Table 14.1.6. Information about withdrawals due to protocol deviations is presented in Section 10.2. The number of subjects allocated to each analysis set is shown in Section 11.1.
